# The Brain Atlas Concordance Problem: Quantitative Comparison of Anatomical Parcellations

**DOI:** 10.1371/journal.pone.0007200

**Published:** 2009-09-29

**Authors:** Jason W. Bohland, Hemant Bokil, Cara B. Allen, Partha P. Mitra

**Affiliations:** Cold Spring Harbor Laboratory, Cold Spring Harbor, New York, United States of America; Indiana University, United States of America

## Abstract

Many neuroscientific reports reference discrete macro-anatomical regions of the brain which were delineated according to a brain atlas or parcellation protocol. Currently, however, no widely accepted standards exist for partitioning the cortex and subcortical structures, or for assigning labels to the resulting regions, and many procedures are being actively used. Previous attempts to reconcile neuroanatomical nomenclatures have been largely qualitative, focusing on the development of thesauri or simple semantic mappings between terms. Here we take a fundamentally different approach, discounting the names of regions and instead comparing their definitions as spatial entities in an effort to provide more precise quantitative mappings between anatomical entities as defined by different atlases. We develop an analytical framework for studying this brain atlas concordance problem, and apply these methods in a comparison of eight diverse labeling methods used by the neuroimaging community. These analyses result in conditional probabilities that enable mapping between regions across atlases, which also form the input to graph-based methods for extracting higher-order relationships between sets of regions and to procedures for assessing the global similarity between different parcellations of the same brain. At a global scale, the overall results demonstrate a considerable lack of concordance between available parcellation schemes, falling within chance levels for some atlas pairs. At a finer level, this study reveals spatial relationships between sets of defined regions that are not obviously apparent; these are of high potential interest to researchers faced with the challenge of comparing results that were based on these different anatomical models, particularly when coordinate-based data are not available. The complexity of the spatial overlap patterns revealed points to problems for attempts to reconcile anatomical parcellations and nomenclatures using strictly qualitative and/or categorical methods. Detailed results from this study are made available via an interactive web site at http://obart.info.

## Introduction

**Figure 1:**
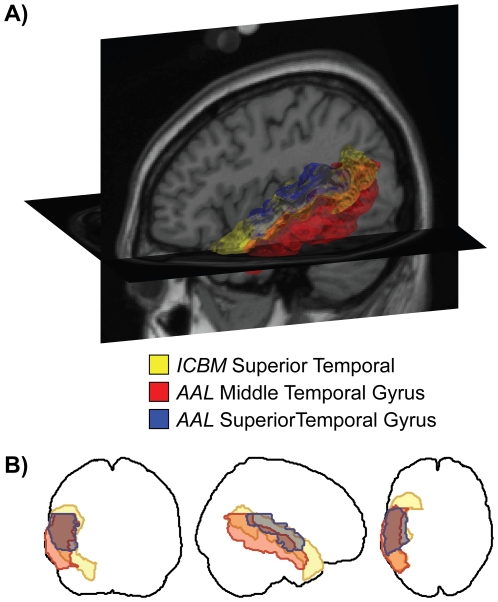
Illustration of the brain atlas concordance problem. A: Rendering of three anatomical regions in the left temporal lobe as delineated by two different brain atlases. The largest region, *Superior Temporal* from the ICBM atlas (see Materials and Methods for atlas descriptions), shown in yellow, overlaps both the *Superior Temporal Gyrus* (blue) and the *Middle Temporal Gyrus* (red) regions in the AAL atlas to differing degrees. B: The same region boundaries drawn as projections in the three cardinal directions. An examination of the patterns of overlap in just 3 regions points to the complexity of the concordance problem.

In this paper we examine the *brain atlas concordance problem*, an issue that stems from difficulties and differences in the description of brain structures, and that presents certain obstacles for the neuroscience research community. The basic premise of this problem, illustrated briefly in Figure 1, is that multiple different methods exist for partitioning a brain into a discrete set of anatomical regions (i.e. *parcellating*), yet we currently lack a thorough understanding of the relationships between different schemes or the potential challenges that discordant parcellations pose, particularly for meta-analyses and other information integration efforts. In Figure 1 we show three anatomical regions rendered in a single reference brain as delineated by two different atlases (the International Consortium for Brain Mapping anatomical template (ICBM), and the Automated Anatomical Labeling atlas (AAL [1]); see Materials and Methods) available to the neuroimaging community. The region in yellow labeled *Superior Temporal*, in the ICBM atlas, overlaps multiple regions in the AAL atlas, just two of which are shown in blue (*Superior Temporal Gyrus*) and in red (*Middle Temporal Gyrus*). The pattern of overlap is complex: for example, approximately 33% of the yellow *Superior Temporal* region's volume is contained in the blue *Superior Temporal Gyrus* region, with another 36% contained in the red *Middle Temporal Gyrus*. However, some 71% of the blue region's volume is contained within the larger yellow parcel, while only 35% is contained within the red. While the details of such overlap calculations will be described below, it is immediately and intuitively clear that there is no simple mapping between these regions as defined by the two example atlases.

This brain atlas concordance problem has traditionally been seen as a neuroanatomical *nomenclature problem*, and neuroscientists have struggled with the terminological heterogeneity in the field for over a century [2]. The issue has generally been viewed as encompassing two key elements: i) multiple distinct terms are sometimes used to refer to the same anatomical or functional brain region, and ii) the same term is sometimes used to refer to different regions. Thus the problem is often cast as terminological in nature and has been addressed primarily through the compilation of large lists of neuroanatomical region labels [3], attempts to build thesauri for relating these terms [4], and recently by developing machine-readable controlled vocabularies and ontologies [5], [6], [7], [8], [9]. In the example given above, on the basis of name alone, it might be expected that the yellow ICBM *Superior Temporal* region should roughly coincide with the blue AAL *Superior Temporal Gyrus* region, but this is not the case. It is thus evident that reconciling published results that reference regions from one anatomical atlas with those that reference another requires more than matching region names, but also developing a precise, quantitative understanding of the correspondence between the different underlying anatomical partitions.

While the situation we consider is not exclusive to human brain imaging, it is here that it is particularly pronounced while also most amenable to analysis. Magnetic resonance imaging (MRI) today is the most common method for visualizing human neuroanatomy, but under usual conditions the details used to define classical region boundaries (e.g. cytoarchitecture) are not observable. The parcellation of MR images, therefore, involves either inferring such boundaries from prior data or using observable landmarks such as sulci as the basis for delineating regions. Several difficulties, including a dearth of consistently identifiable landmarks, inter-subject variability, and imprecise or indeterminate structural-functional relationships, have led to the community's inability to adopt a standard parcellation protocol. Still a variety of different methods are commonly used to parcellate MR images, serving a number of practical purposes. These include reducing the total volume of data, establishing anatomical correspondences between individual subjects, and providing a discrete framework in which to communicate results. Explicit *a priori* parcellation of macro-anatomical regions from individual MR volumes is sometimes used, for example in region-of-interest (ROI) based functional analysis or morphometric analysis [10], [11], [12], [13]. More commonly however, parcellation is implicit and occurs *post hoc* when researchers endeavor to label the voxels that show statistical effects of interest in their experiments. In either case, reference is often made to one or more digital atlases that provide the model by which brains are partitioned and the individual regions named. Thus, while most analyses of brain imaging data do not directly depend on anatomical parcellations, the way in which results are reported, interpreted, and compared with previous studies can be heavily influenced by the choice of anatomical reference atlas.

A growing number of anatomical atlases have appeared in the neuroimaging community, some of which have been integrated within popular software tools for statistical data analysis. It is important to distinguish between stereotactic reference frames, which define a coordinate space in which anatomical volumes may be registered, and anatomical atlases or parcellations which may be defined within such a space, but which serve to partition the volume into a discrete set of labeled regions. Whereas some degree of standardization has been achieved in terms of coordinate systems, anatomical labeling methods are considerably more variable. While the Talairach Atlas [14], [15] established a large early “market share” in positron emission tomography (PET) and functional MRI studies, it is now challenged by a variety of other anatomical models as the community becomes increasingly aware of its limitations [16], [17]. Moreover, various groups have developed protocols for expert manual parcellation of MR volumes [13], [18], [19], [20], [21], and new tools are being developed to perform automatic parcellation of a given MR volume based on a set of manually labeled training exemplars [e.g. 22], [23], [24]. Thus the number of parcellation methods available to researchers is increasing, in turn amplifying the need for informatics procedures that capture the relationships between different protocols and enable mapping between them. Significant progress has been made to enable registration and visualization of different data sets and partitioning schemes across atlases, individuals, and species in the Surface Management System Database (SumsDB; http://sumsdb.wustl.edu/sums) [25], [26]. The ability to “overlay” different partitions upon one another, as enabled by SumsDB and other tools, is critical for making quantitative cross-comparisons. Recently, spatially registered surface-based parcellations of the macaque cortex from this resource have been studied quantitatively [27] in a spirit similar to our current presentation for volumetric human atlases.

For the present investigation, it is important to note that the wide variety of labeling methods in use in neuroimaging today each have been designed for a particular purpose with particular anatomical bases, and with varying degrees of, sometimes space-variant, granularity in their delineations. Further, different methods rely upon, and potentially suffer ambiguity from, the variable ways in which individual brains can be *registered* to a common template. It is thus difficult, in general, to fully separate the consequences of registration variability and error from those of differences in the underlying anatomical partitions for different methods. In the present study we did not seek to specifically disentangle these different aspects of brain labeling, but rather sought to compare the *net consequences* of using a variety of different common procedures to label the same brain, among them including manual parcellation of the test brain itself, application of probabilistic atlases in a common template space, and mapping to an individual anatomical template. Because our primary goal was to provide a method to begin to understand the state of extant results that have been variably reported using these different anatomical frameworks, and not specifically to decipher the underlying intent of the developers of the atlases, incorporating such heterogeneous methods in our study is obligatory. Based on the different underlying references for the various methods, however, we could hypothesize that similar methods (e.g. two manual parcellations of the same brain) would give highest overall concordance, whereas methods that relied on different references and/or registration schemes would likely have lower degrees of similarity.

The difficult task of integrating research results across studies that employ varied methods is of increasing consequence as the volume of published work continues to grow rapidly in the neuroimaging field and in neuroscience more generally. Text-mining tools are being developed to automatically or semi-automatically extract terms and concepts from research articles to populate knowledge bases [28], [29], [30] that should ultimately be accessible in a common anatomical framework. We posit that the proper integration of knowledge about specific anatomical regions requires an appropriate theoretical framework for mapping between different atlases, which will lead to a *quantitative* understanding of the correspondences between parcellations, and which will provide the necessary evidence for best reconciling heterogeneously reported results.

In the present investigation we develop and apply this framework, casting the brain atlas concordance problem as an analysis of the spatial relationships between different partitions of the same “space”–or underlying anatomy. From this perspective, we seek to discover the quantitative spatial relationships between all pairs of regions across a set of anatomical parcellations. Pair-wise relationships can be expressed using simple conditional probabilities, providing answers to straight-forward questions of the form: *what is the probability that a voxel is in Region X according to Method A if it is in Region Y according to Method B?* In this framework the potential impact of the use of multiple discordant parcellation schemes in the published literature becomes clear. If regions from one atlas can not be mapped to regions from another atlas with high probability, then reconciling results based on these two anatomical references using region labels alone will lack certainty.

The quantitative procedures developed here are used to compare eight different parcellations of the left-hemisphere gray matter regions in a high-resolution MR volume (see Table 1 and Materials and Methods for details). Beyond the computation of region-to-region conditional probabilities, we also establish methods for visualizing the large resulting dataset, introduce procedures to uncover higher-order relationships between sets of regions, and develop the idea of *global similarity* between two whole-brain parcellations. The concept of “chance” similarity is additionally derived from a series of *random parcellations* of the test brain and used to establish significance measures for comparing parcellations. The overall results reveal a set of complex correspondences between different atlas structures, information that is valuable in a variety of contexts, and that has not been previously described. For some atlas pairs, we find a surprising lack of concordance, and we discuss the reasons and implications for such findings. The detailed results of this study are also made available at http://www.obart.info, which we hope will assist researchers attempting to interpret the existing literature, choosing atlasing methods, or developing new analytical procedures for their own studies.

**Table 1:** Summary of parcellation methods compared in this study.

Method	# LH regions	% GM labeled	Reference space	Brief Description
AAL	62	93.2%	Colin27	Manual parcellation of Colin27 atlas
CYTO	29	21.6%	MNI average	Maximum likelihood cytoarchitectonic atlas in MNI space
H-O	56	86.7%	MNI average	Maximum likelihood atlas from manually labeled scans
ICBM	49	92.5%	Colin27	Individual parcellation of Colin27 atlas
LPBA40	29	97.0%	MNI average	Maximum likelihood from manually labeled scans
T&G	65	81.1%	Colin27†	Freesurfer-classified individual atlas, tweaked by human expert
TALc	68	26.5%	Talairach brain	Brodmann's area labels mapped to MNI space with icbm2spm
TALg	49	76.7%	Talairach brain	Gyrus-level Talairach atlas mapped to MNI as above

† The T&G method as used here also relies on the Freesurfer average subject atlas.

## Results

### Region-level concordance analysis

**Figure 2:**
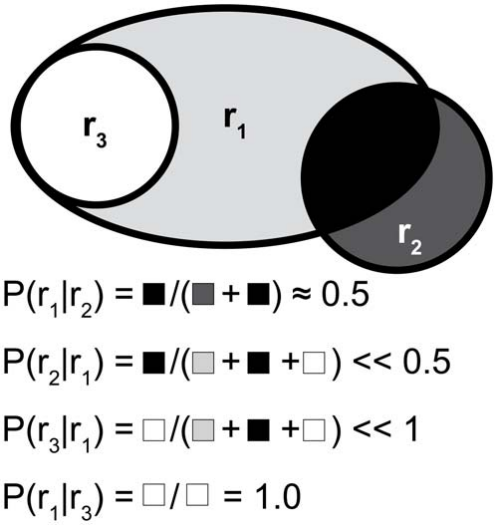
Venn diagram illustrating the formulation of conditional probability measures *P_ij_*. Three different hypothetical regions *r_1_*, *r_2_*, and *r_3_* are shown in two dimensions in different spatial arrangements. At bottom, the calculation of each conditional probability based on the areas (volumes in 3-D) of the shaded regions is shown.

We analyzed the pair-wise spatial correspondences between anatomical regions defined in eight distinct parcellations of a single test brain, the ICBM single-subject template. For any pair of regions, two conditional probability values were calculated based on the spatial overlap between the parcels (see Equation 3 and Figure 2). For simplicity, we write the probability that a voxel *x* is in region *i* in one parcellation given that it is in region *j* in another parcellation as 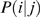. Because the regions within a given parcellation *R* are spatially disjoint, 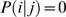 if regions *i* and *j* are both drawn from *R*. The Venn diagram in Figure 2 illustrates the lack of symmetry in these spatial relations. If region *j* is wholly contained in region *i*, then 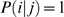, while 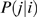 will also equal 1 only when the two regions are identical.

**Figure 3:**
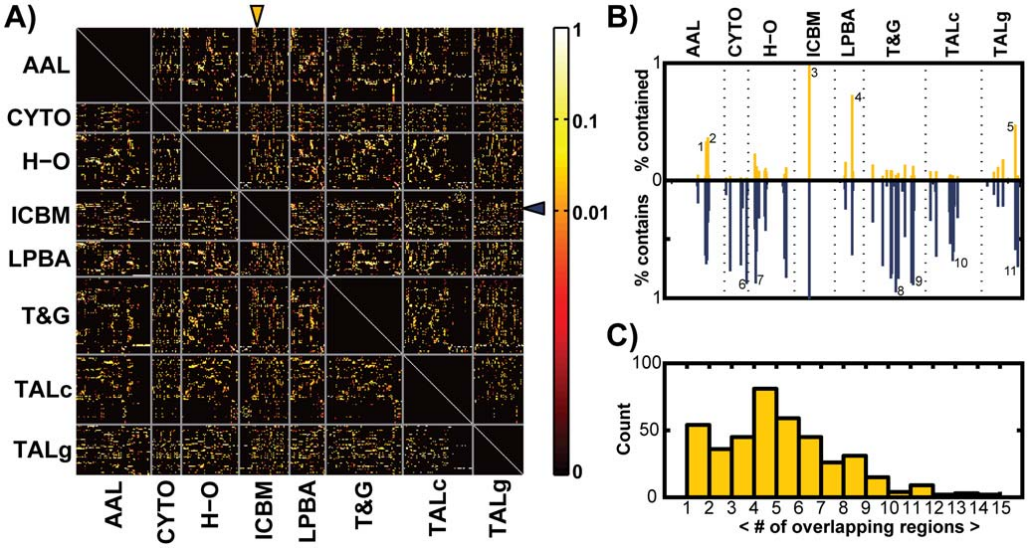
Region-level concordance analysis across eight anatomical parcellations. A: Overall non-symmetric concordance matrix *P*. Entry *P_ij_* gives *P*(*i|j*), the probability that a voxel is in region *i* given that it is in region *j* in another parcellation scheme. Each row and column corresponds to a particular anatomical region, and regions are grouped by parcellation method (separated by the gray horizontal and vertical lines). B: The column (top) and row (bottom) from the matrix *P* corresponding to the *Superior Temporal* region as delineated by the ICBM atlas (see arrows in *A*) were extracted and the corresponding conditional probability values rendered as the heights of bars. The orange bars give the fraction of the *ICBM Superior Temporal* region contained in other regions, and the blue bars (below) give the fraction of other regions contained in the ICBM region. The names of the example overlapping regions corresponding to the annotated bars are as follows: 1. AAL superior temporal gyrus; 2. AAL middle temporal gyrus; 3. ICBM superior temporal; 4. LPBA40 superior temporal gyrus; 5. TALg superior temporal gyrus; 6. CYTO TE1.2; 7. H-O superior temporal gyrus, anterior division; 8. T&G anterior superior temporal gyrus; 9. T&G posterior dorsal superior temporal sulcus; 10. TALc Brodmann Area 41; 11. TALg transverse temporal gyrus. C: Histogram of the mean number of regions from any parcellation *R′* that overlap a single region drawn from a different parcellation *R*.

Figure 3(A) shows the overall results of this region-level analysis across the eight parcellations. The matrix of conditional probabilities *P* is depicted as an image, with each pixel's color indicating the value of that matrix entry (on a logarithmic scale). Each row and column corresponds to one particular anatomical region in a parcellation, and regions are grouped by parcellation method (the rows or columns between sets of grey lines). Non-zero (non-black) entries indicate that two regions exhibit some degree of spatial overlap, and it is apparent that overlap is often partial between region pairs. While the results contained in matrix *P* are too numerous to describe individually here, an annotated software tool is available online (http://www.obart.info) that allows the interested reader to view the findings interactively and in full detail. Presently, we provide further explication for a single illustrative brain region, continuing to expand on the example from Figure 1.

Figure 3(B) shows the values from the column and row of *P* corresponding to the *Superior Temporal* region as defined by the ICBM atlas (see *Materials and Methods* for details of each parcellation scheme) rendered as the heights of bars. A single column in *P* contains the probabilities that a voxel is in each of the other anatomical regions given that it is in the ICBM *Superior Temporal* region (Figure 3B, top bar plot). The corresponding row in *P* contains the probabilities that a voxel is in this ICBM region given that it is in each of the other parcels (Figure 3B, bottom bar plot). It is apparent that the set of voxels labeled *Superior Temporal* by the ICBM atlas is assigned multiple labels by other atlases, including but not limited to “superior temporal gyrus” and its derivatives. Still we observe no equivalent or nearly equivalent unit in any of the other atlases, and the conditional probabilities rendered in Figure 3B are largely asymmetric. The utility of the conditional overlap measure is apparent as it observed, for instance, that multiple regions from the *T&G* parcellation are contained to a large degree within this ICBM region, but not vice versa; thus these regions are (largely) subsets of the ICBM *Superior Temporal* region, information which could not be “read off” as such if a different measure of overlap (e.g. Jaccard index) had been used.

From the conditional matrix *P*, a symmetric overlap matrix *O* was also computed (Equation 4). From *O* (see Figure S1 for illustration), some simple statistical properties were calculated that characterize the problem of mapping between different parcellations, e.g. the number of regions *K* in any parcellation *R′* that overlap a single region drawn from parcellation *R*. This number offers some insight into the overall ambiguity in the mapping problem between atlases, with larger *K* indicating increasing uncertainty. For each region, we calculated the average number of partially overlapping areas in the other 7 parcellations. Figure 3(C) shows a histogram of these values. The mean and median of this distribution are 4.95 and 4.71, respectively.

For each pairing of atlases, we ranked region pairs in order of decreasing symmetric overlap value in order to reveal the most similar region definitions directly. The top 5 region-region overlaps for each atlas pair are shown in Table 2. The full overlap matrix for any atlas pair can also be downloaded from http://obart.info. Notably, the most similar regions tended to be large subcortical structures (e.g. thalamus, putamen), whose boundaries (and nomenclature) are more well-determined than in the cerebral cortex, where delineations are more arbitrary, although there were certain exceptions. Even the highest region-level agreement between areas from the CYTO cytoarchitectonic parcellation and those in other atlases tended to be relatively low, which was anticipated because no other methods compared used direct analysis of cytoarchitecture as the basis for region definitions. Determining how the voxels most likely to be classified as part of a particular cytoarchitectonic area overlap with voxels classified by other methods (based largely on sulcal/gyral landmarks) is potentially of high interest to researchers now using the CYTO atlas in their work, but who are faced with much previous literature referencing these other schemes. Some of the pairs in highest agreement (e.g. Area-44 with the inferior frontal gyrus *pars opercularis* in the T&G parcellation and Area-45 with the T&G inferior frontal gyrus *pars triangularis*) were as traditionally expected, but these results provide quantification of such assumed relationships between terminologies. The TALc parcellation also uses cytoarchitectonic *nomenclature* in the form of Brodmann's areas (though the locations of these areas in the Talairach atlas were only approximate), but TALc regions did not strongly match CYTO regions, or correspond particularly well with regions from any other parcellation.

**Table 2:** Top 5 best matching region definitions for each atlas pair, scored using symmetric overlap matrix *O*.

	O_ij_	CYTO	O_ij_	H-O	O_ij_	ICBM	O_ij_	LPBA	O_ij_	T&G	O_ij_	TALc	O_ij_	TALg
**AAL**	0.62	Calcarine	0.84	Thalamus L	0.91	Putamen L	0.81	Caudate L	0.86	Thalamus L	0.66	Putamen L	0.74	Thalamus L
		Area 17		Thalamus		Putamen		L caudate		left-Tha		Putamen		Thalamus
	0.60	Frontal Inf Tri	0.81	Putamen L	0.89	Caudate L	0.81	Putamen L	0.83	Putamen L	0.58	Caudate L	0.68	Caudate L
		Area-45		Putamen		Caudate Nucleus		L putamen		left-Put		Caudate Body		Caudate
	0.57	Frontal Inf Oper	0.71	Caudate L	0.80	Pallidum L	0.72	Occipital Mid L	0.76	Hippocampus L	0.44	Thalamus L	0.63	Putamen L
		Area-44		Caudate		Globus pallidus externa		L middle occipital gyrus		left-Hipp		Medial Dorsal Nucleus		Lentiform nucleus
	0.51	Hippocampus	0.66	Postcentral L	0.79	Frontal Mid L	0.70	Frontal Mid L	0.74	Pallidum L	0.41	Thalamus L	0.59	Temporal Mid L
		Hipp-CA		Postcentral gyrus		Middle frontal gyrus		L middle frontal gyrus		left-Pal		Pulvinar		Middle temporal gyrus
	0.50	Supp Motor Area	0.66	Precuneus L	0.79	Fusiform L	0.66	Hippocampus L	0.69	Caudate L	0.39	Temporal Pole Mid L	0.55	Postcentral L
		Area-6		Precuneous cortex		Fusiform gyrus		L hippocampus		left-Caud		Brodmann area 38		Postcentral gyrus
**CYTO**			0.57	Amyg-SF	0.66	Hipp-SUB	0.57	Area-17	0.55	Area-44	0.60	Amyg-LB	0.56	Area-17
				Amygdala		Parahippocampal gyrus		L lingual gyrus		left-iFo		Amygdala		Lingual gyrus
			0.55	Area-17	0.65	Hipp-CA	0.51	Hipp-SUB	0.54	Area-45	0.40	Hipp-CA	0.50	Hipp-EC
				Intracalcarine cortex		Hippocampus		L parahippocampal gyrus		left-iFt		Hippocampus		Uncus
			0.53	Area-45/	0.54	Area-6	0.45	Hipp-CA	0.43	Hipp-CA	0.35	OP-4	0.41	Area-4a
				Inferior frontal gyrus triangularis		Precentral gyrus		L hippocampus		left-Hipp		Brodmann area 43		Paracentral lobule
			0.52	Area-44	0.54	Area-17	0.42	Area-18	0.43	Amyg-SF	0.31	Amyg-CM	0.40	Area-1
				Inferior frontal gyrus opercularis		Lingual gyrus		L cuneus		left-Amyg		Lateral globus pallidus		Postcentral gyrus
			0.52	Hipp-CA	0.45	Area-44	0.42	Area-6	0.41	OP-1	0.29	Hipp-EC	0.35	Hipp-CA
				hippocampus		Inferior frontal gyrus		L precentral gyrus		left-PO		Brodmann area 28		Parahippocampal gyrus
**H-O**					0.82	Putamen	0.86	Putamen	0.90	Thalamus	0.61	Caudate	0.70	Thalamus
						Putamen		L putamen		left-Tha		Caudate body		Thalamus
					0.70	Precuneous_Cortex	0.84	Insular cortex	0.84	Putamen	0.59	Putamen	0.62	Postcentral gyrus
						Precuneus		L insular cortex		left-Put		Putamen		Postcentral gyrus
					0.70	Precentral_Gyrus	0.84	Precentral gyrus	0.81	Frontal_Pole	0.55	Accumbens	0.61	Putamen
						Precentral gyrus		L precentral gyrus		left-FP		Caudate head		Lentiform nucleus
					0.69	Hippocampus	0.78	Postcentral gyrus	0.73	Pallidum	0.41	Temporal pole	0.58	Precentral gyrus
						Hippocampus		L postcentral gyrus		left-Pal		Brodmann area 38		Precentral gyrus
					0.69	Caudate	0.74	Precuneous cortex	0.72	Caudate	0.41	Cingulate gyrus anterior	0.57	Caudate
						Caudate nucleus		L precuneus		left-Caud		Brodmann area 24		Caudate
**ICBM**							0.95	Cerebellum	0.91	Cerebellum	0.70	Putamen	0.76	Caudate nucleus
								cerebellum		left-CBctx		Putamen		Caudate
							0.83	Caudate nucleus	0.83	Putamen	0.63	Caudate nucleus	0.66	Putamen
								L caudate		left-Put		Caudate Body		Lentiform nucleus
							0.83	Putamen	0.68	Globus pallidus externa	0.43	Caudate Nucleus	0.60	Supramarginal gyrus
								L putamen		left-Pal		Caudate Head		Inferior parietal lobule
							0.76	Lingual gyrus	0.68	Hippocampus	0.40	Supramarginal gyrus	0.58	Middle frontal gyrus
								L lingual gyrus		left-Hipp		Brodmann area 40		Middle frontal gyrus
							0.72	Inferior frontal gyrus	0.68	Precuneus	0.40	Superior parietal gyrus	0.57	cingulate gyrus
								Inferior frontal gyrus		left-PCN		Brodmann area 7		Cingulate gyrus
**LPBA**									0.89	cerebellum	0.67	L putamen	0.71	L putamen
										left-CBctx		Putamen		Lentiform nucleus
									0.81	L putamen	0.57	L caudate	0.66	L caudate
										left-Put		Caudate body		Caudate
									0.72	brainstem	0.41	L supramarginal gyrus	0.65	L superior temporal gyrus
										right-BrSt		Brodmann area 40		Superior temporal gyrus
									0.64	L caudate	0.35	L superior parietal gyrus	0.63	L postcentral gyrus
										left-Caud		Brodmann area 7		Postcentral gyrus
									0.62	L hippocampus	0.35	L caudate	0.62	L middle temporal gyrus
										left-Hipp		Caudate head		Middle temporal gyrus
**T&G**											0.61	left-Put	0.74	left-Tha
												Putamen		Thalamus
											0.47	left-Acc	0.61	left-Put
												Caudate head		Lentiform nucleus
											0.42	left-Caud	0.45	left-PT
												Caudate body		Transverse temporal gyrus
											0.42	left-Tha	0.45	left-PCN
												Pulvinar		Precuneus
											0.39	left-Tha	0.43	left-pSMg
												Medial dorsal nucleus		Inferior parietal lobule
**TALc**													0.90	Putamen
														Lentiform nucleus
													0.76	Caudate body
														Caudate
													0.63	Caudate head
														Caudate
													0.57	Brodmann area 13
														Insula
													0.54	Pulvinar
														Thalamus

In each cell, the first region is from the atlas indicated by the row header, and the second from the atlas indicated using the column header. Decimal values indicate overlap according to *O*.

**Figure 4:**
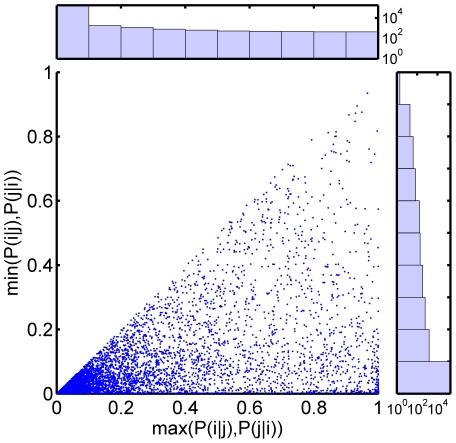
Scatter plot of computed spatial conditional probability values for all region pairs. For each two regions (*r_i_*, *r_j_*), max(*P*(*i*|*j*), *P*(*j*|*i*)) is plotted vs. min(*P*(*i*|*j*), *P*(*j*|*i*)) (see also [27]). Histograms shown adjacent to either axis are log scale counts of these measures taken across all region pairs.

Following Bezgin et al. [27], we also plotted all of the computed pairs of spatial conditional overlap values against one another, with the larger conditional probability for each region pair always plotted as the x-coordinate in the scatter plot (Figure 4). In this depiction, some approximate relationships can be deduced based on the position of the point representing a single region pair in the plane. Those region pairs represented by the many points near (0,0) are nearly disjoint, while those near (1,1) are closely matching. Points clustered along or near the line *x = *1 correspond to approximate subset relationships (one region is contained within the other larger region). Still, based on the scatter plot density (depicted also as log histograms along either axis), it is apparent that many region-region relationships are in the interior coordinate plane–with relatively low, but non-zero values for both conditionals. Such “overlap” relations [31], which vary asymmetrically, are problematic for terminological ontologies that rely on simple categorical mappings and therefore lose information relative to the pair of conditional probability values we compute here.

### Visualization of region-level concordance results

**Figure 5:**
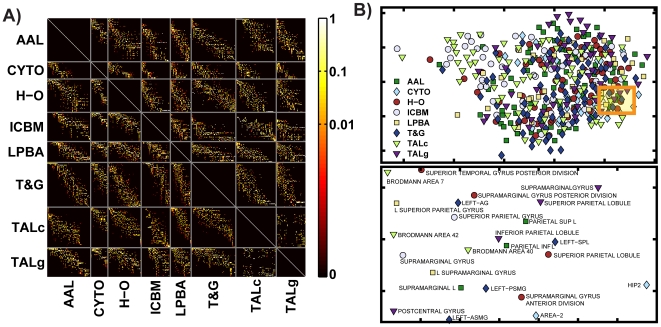
Visualization of region-level concordance results. A: *P_ij_* matrix after permuting the indices (region labels) independently within each block in order to reduce matrix bandwidth. The blocks can not be completely diagonalized because of the lack of one-to-one correspondence between regions in pairs of parcellations. B: Visualization of region labels using multi-dimensional scaling. *Top:* 2-D landscape of computed coordinates for each anatomical region, with the parcellation from which each region is drawn indicated by the marker type. *Bottom*: magnified portion of the 2-D landscape above revealing the anatomical regions and their labels that occupy this segment of the space (the highlighted rectangular area in *top*).

The ordering of regions in each parcellation as depicted in Figure 3(A) is somewhat arbitrary, and thus visually determining the degree of correspondence between two parcellations is difficult. By rearranging the rows and columns (the ordering of regions), in the matrix, the interpretation of results is made easier. A heuristic based on the singular value decomposition (SVD; see Materials and Methods) was applied to permute the rows and columns of each rectangular block in *C* independently in order to minimize the matrix bandwidth (the maximum distance of non-zero entries from the diagonal) for that block. The result of applying this reordering algorithm is shown in Figure 5(A). It can be seen that, although non-zero conditional probability values have been moved toward the diagonal, there remains considerable bandwidth for each block, which is due to the general lack of one-to-one correspondences between regions. Still, this procedure provides a one-dimensional embedding for region labels; i.e. regions with similar spatial definitions appear nearby in this space. This is useful to impose a meaningful order on the sets of region labels when comparing two parcellations.

The set of anatomical region labels from the different parcellations considered were also plotted in two-dimensional space using multi-dimensional scaling (MDS). In this visualization method, similarly defined (e.g. overlapping) anatomical entities are assigned to nearby points in the 2D space while spatially disparate entities appear more distant. Figure 5(B) shows the results of applying MDS using a dissimilarity matrix derived from the symmetric overlap matrix *O*. The result provides the observer with a means to visually determine which anatomical regions drawn from multiple parcellation schemes are most similar to one another prior to delving deeper into, for example, the precise conditional probability values for the many available region pairs.

### Higher-order spatial relationships

**Figure 6:**
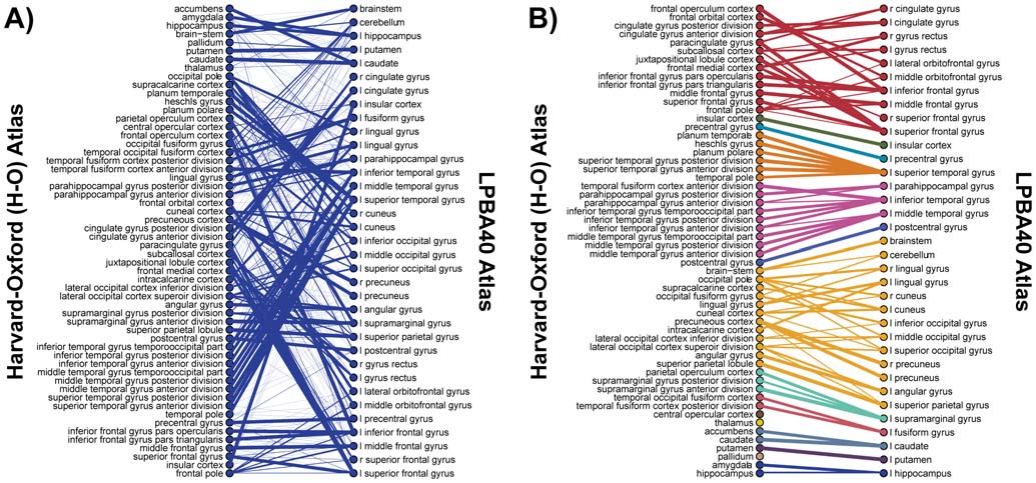
Extraction of approximate higher order spatial relationships using bipartite graphs. A: Initial bipartite graph constructed by connecting vertices on the left (corresponding to the *H-O* parcellation) with vertices on the right (corresponding to the *LPBA40* parcellation) when the corresponding parcels overlap. The undirected edge weights were determined by the maximum conditional probability value for each region pair. The graph is fully connected. B: Final bipartite graph representation of the same parcellations after pruning all edges with weight less than 0.25. The graph is partitioned into 9 connected components (rendered in different colors); for each component, the union of regions on the left is approximately equivalent to the union of regions on the right.

The matrix formulations described above provide measures of the pair-wise correspondences between anatomical parcels, but they do not directly capture what we refer to as *higher-order* spatial relationships. For example, it may be the case that the union of *m* regions from one atlas is approximately spatially equivalent to the union of *n* regions from another. We applied a simple graph theoretical algorithm that uses the conditional probability values from matrix *P* to extract such relationships between region definitions in any pair of atlases. *Bipartite graphs*, with regions from each parcellation represented as distinct sets of vertices, were constructed, with edges drawn between two vertices when the two corresponding regions overlap. Figure 6(A) shows the initial bipartite representation of the correspondence between two parcellations based on probabilistic atlases of similar scope, the Harvard-Oxford (H-O) and the LPBA40 atlases. The graph is *connected* (i.e. a path can be drawn from any vertex to any other vertex), and no significant region groupings can be easily identified.

In Figure 6(B), the graph is rendered after pruning all edges with weights *E_ij_<0.25*, partitioning the graph into multiple individually connected components. Each resulting component is rendered in a different color, and the vertices (regions) have been reordered for improved visualization. While this procedure uncovered certain approximate higher-order correspondences that were not obvious in Figure 6(A), it is evident that such congruence between these partitions occurs primarily at a rather large spatial scale (e.g. the first connected component corresponds approximately to the entire frontal lobe). In most cases, such correspondences emerged only after the removal of many edges that represent substantial degrees of overlap between regions. Still, from Figure 6(B) (and from visualizations with other intermediate thresholds not shown here but available online), it is possible to note interesting relationships between regions, a few of which are:

1. The LPBA40 *superior temporal gyrus* largely contains 6 regions in the H-O atlas, including parcels labeled the *anterior and posterior divisions of the superior temporal gyrus*, but also *Heschl's gyrus, Planum Polare, Planum Temporale*, and the *Temporal pole*.
2. What is called the *caudate* by LPBA40 is subdivided into *caudate* and *accumbens* in H-O, and what is called *putamen* in LPBA40 largely contains both the *putamen* and *pallidum* in H-O.
3. The supramarginal gyrus as defined by LPBA40 largely contains two subdivisions of a parcel with the same name in the H-O atlas, but also contains almost 80% of a region called the *parietal operculum cortex*, a term not used in the LPBA40 atlas. Notably, a search of NeuroNames [5] does not reveal any correspondence between these labels.

These graph-based depictions as shown in Figure 6(B) provide a simple, straight-forward means for understanding the major spatial relationships between two parcellations. We thus provide bipartite graph depictions for each atlas pair, with edge thresholds of 0.10 and 0.25, in Text S1. The accompanying web tool (http://obart.info) additionally allows the user to select custom edge thresholds for the graph depicting any atlas pair, providing an interactive resource to enable a better understanding of the complex relations, which are far too numerous to describe in full here. A simple use case, for example, would be to look up the regions from the 8 methods studied that most overlap a particular area of interest in the atlas with which a researcher is most familiar.

### Global atlas concordance

While the above results revealed both simple and highly complex correspondences between region definitions owing to multiple atlasing methods, we additionally sought to provide a single scalar-valued index of *global concordance* between two parcellations. We calculated the *Adjusted Rand Index* (ARI [32]) as well as a novel *S index* (SI), which was developed for this specific application, for each pair of brainwide neuroanatomical parcellations (excluding the *CYTO* parcellation, which only provides a partial labeling of the brain).

**Figure 7:**
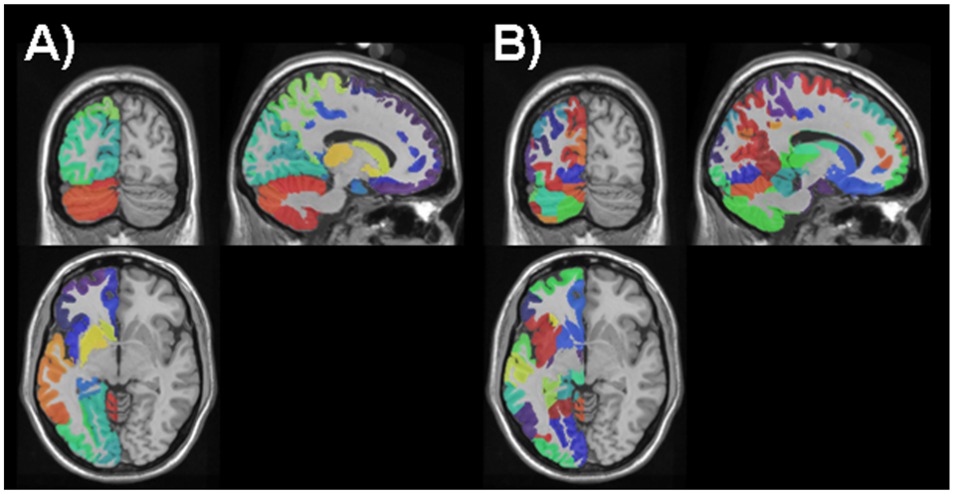
Random parcellations. A: Sections through the AAL parcellation of the test brain with different colors indicating different parcels. B: a random parcellation of the same test brain.

The values of such scalar indices are typically difficult to interpret in the absence of known distributions of the values expected by chance. To compute such chance concordance distributions, we compared *random parcellations* of the test brain. We used a simple algorithm (see Materials and Methods) to create random space-filling partitions of the gray matter voxels consisting of *N* contiguous regions. Fifty random parcellations were generated for each of the atlases examined, with *N* matched to the number of regions comprising each atlas. Figure 7 shows several sections through an actual parcellation (*AAL*) as well as a size-matched random parcellation. For each pair of atlases, the similarity indices for 1000 pairs of size-matched random parcellations were calculated, yielding an estimate of the chance distribution specific to each pair-wise comparison.

**Figure 8:**
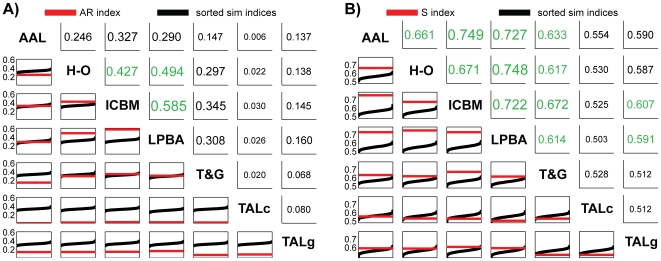
Global concordance between parcellations. A: Results using Adjusted Rand Index (ARI). B: Results using the S index. In each subfigure the values in the upper diagonal entries are the concordance indices for particular pairs of atlases, with above chance values in green. The lower diagonal entries show the sorted distribution of chance concordance values obtained by comparison of 1000 random size-matched parcellations (curved line) and the actual value obtained for this atlas pair as a horizontal line.

**Figure 9:**
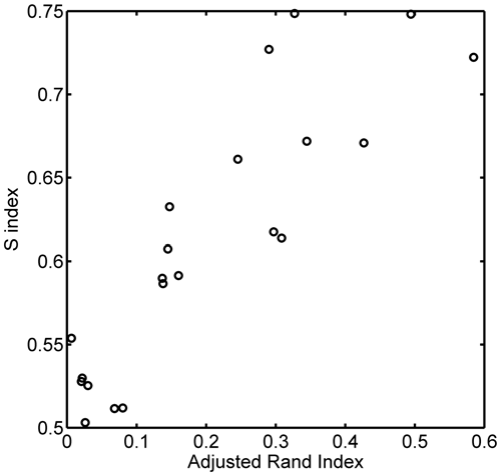
Comparison of Adjusted Rand Index and S index. The values of both computed indices of global concordance plotted against each other for each atlas pair.

Figure 8(A) shows the global atlas concordance results as calculated using the Adjusted Rand Index. The computed ARI for each pair of atlases is shown in the above diagonal entries, with those values appearing in green exceeding the 95^th^ percentile index in the corresponding chance similarity distribution. Shown in the corresponding sub-diagonal entries are the 1000 sorted chance similarity values obtained by simulation (black curve), as well as a red horizontal line indicating the ARI for the true atlas pair. Global atlas concordance as assessed by the ARI was surprisingly poor, with many parcellation pairs judged to be concordant at or below chance levels. Because the ARI penalizes anatomical region refinement (subset or hierarchical relationships), however, we designed and utilized a second similarity index (SI; see Equation 7) intended to better capture the notion of global concordance for atlases that may contain brain parcels subdivided with different levels of granularity. The global concordance results computed using SI are shown in Figure 8(B). Here, most of the concordance values for pairs of parcellations rise above chance levels, as would be expected for parcellations of the same brain that are, in most cases, based on sulcal/gyral landmarks. As hypothesized, the two parcellations that used the test brain directly as a reference (AAL and ICBM) had the highest overall concordance, and the two probabilistic atlases of similar scope (H-O and LPBA40) had the next highest similarity. Still it is worth noting that no concordance values observed approached the maximum theoretical value of 1, and several pairs, particularly those involving the Talairach-based parcellations, remain at or below the concordance values expected by chance according to simulations. Figure 9 illustrates the relationship between the ARI and the new S-index. The indices show considerable correlation (*r* = 0.87) but are not so tightly coupled as to be completely redundant.

The global concordance values for the TALc atlas were particularly low in both the ARI and S-index calculations, and this was owing largely to the fact that a considerably lower fraction of the gray matter voxels from the test brain were assigned labels by this method than by the other labeling schemes compared (see Table 1 and further discussion below). To examine this parcellation method further, we created a second “neighborhood” version of the TALc atlas, called TALc^NH^, in which unlabeled GM voxels were assigned labels based on the labels of nearby (neighborhood) voxels (see Materials and Methods). An additional concordance analysis of the TALc^NH^ parcellation is included as Text S2. In brief, this method resulted in ∼79% of GM being labeled (note that much of the cerebellum is not assigned a “cell-level” label in the Talairach atlas) as opposed to just ∼26% in TALc, and boosted global similarity values significantly. We also include additional bipartite graph representations for comparing TALc^NH^ with the other parcellations within Text S2.

Additionally, Table S1 provides the values of the two global concordance measures evaluated separately for voxels judged to be in the cerebral cortex and for voxels judged to be elsewhere (subcortical nuclei, brainstem, cerebellum). While these results are highly variable across atlas pairs, this analysis allows one to observe which partitioning methods agree more or less along these broadest of subdivisions. It is clear, for example, that concordance between TALc and the other methods was always substantially higher subcortically than cortically, again pointing to cortical registration considerations.

## Discussion

In this study we have undertaken the first systematic, quantitative analysis of the relationships between different anatomical parcellation schemes used within the brain imaging community. The *brain atlas concordance problem* occurs not because of disagreements in terminology (cf. descriptions of the neuroanatomical *nomenclature problem*), but because the underlying reference partitions of brain anatomy (e.g. atlases) are, at times, dissimilar. Taking this perspective, we computed conditional probability matrices that relate any brain region in any of the parcellations examined to all others, independent of linguistic label. Thus we see our approach as one based on direct *evidence*; that is, by applying the different anatomical labeling procedures available in the neuroimaging community to a common individual brain, we can refer to specific spatial definitions for each region rather than relying on subjective interpretation about the meaning of particular terms. It should be made clear that the goal of this investigation was not to determine which parcellation is *best* or to advocate one method or another. Indeed, it is highly unlikely that the neuroscience community will, or even should, adopt a single scheme for partitioning the brain or for labeling its pieces. Further, it is clear that the motivations underlying the construction of one atlas can be different from another (e.g. cytoarchitectonic vs. landmark-based, or of different granularity in particular areas). Moreover, multi-dimensional descriptions of brain areas based on multiple atlases, each of different modality (e.g. cytoarchitecture, folding patterns, connectivity, gene expression patterns), are ultimately likely to provide the best windows into brain organization. Nevertheless, in order to make sense of the vast body of imaging studies that make reference to the multiple available parcellation schemes, it is constructive to attain an understanding of how these schemes relate to one another.

### Concordance of atlases used in neuroimaging

By using 8 different methods to parcellate the gray matter within the same individual MRI volume, we were able to directly compute the relative spatial overlaps of all available region pairs (Figures 3 and 4). It is apparent that the voxels within a typical region in one parcellation most often map to multiple anatomical regions in another, with one-to-one or nearly one-to-one relationships being the exception. On average, a single region overlaps more than 4 regions defined in any other parcellation, and sometimes 15 or more (though, note that these numbers can vary with region size). While any particular anatomical region is likely to overlap several regions in different parcellations, the expected value of this number *K* is limited by the spatially contiguous nature of anatomical partitions. This practical upper limit is reflected in the overall sparsity of the matrix *P* (Figure 3A).

The alignment of two parcellations that would, theoretically, enable mapping between the constituent regions with the highest degree of certainty occurs when every region from one parcellation corresponds directly and exclusively to one region from the other. In this scenario, the blocks in matrix *P*, each of which corresponds to the comparison of two parcellation schemes, could be diagonalized by finding a proper permutation of columns and rows. In other words, by rearranging the order of the anatomical regions, the non-zero probabilities could be lined up along the matrix diagonal, providing a useful visualization as well as a meaningful sorting of region labels. Although our SVD-based approach (shown in Figure 5A) does not necessarily yield the optimal solution, in general, lower resulting bandwidth is indicative of better correspondence between the two parcellations. An additional visualization procedure explored here was to map region labels into a two-dimensional space using MDS (Figure 5B), such that similarly defined (overlapping) regions appear in closer proximity with one another than non-overlapping regions. This type of simple intuitive graphical representation stands in contrast to anatomical ontologies, which are often difficult to visualize and frequently fail to capture analog similarity between entities. In Figure 5B, for example, a magnified view of the MDS plot shows a region depicting region labels from each of the 8 parcellations, which are, for the most part, located in and around the junction of the parietal and temporal lobes; the layout, based on spatial analysis, thus gives rise to an understanding of terminological relations (e.g. Brodmann Area 40 is located in the inferior parietal lobe). Labels for the inferior parietal lobule, as defined by TALg and by AAL, are in particularly close proximity near the center of the landscape, reflecting their relatively high overlap value (O_ij_≈0.5).

Building on the conditional region-level concordance measures, we have developed a graph-based method for examining potentially “higher-order” spatial relationships between pairs of parcellations (Figure 6 and Text S1). By removing all edges with weights *E_ij_* less than some threshold 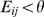 from a bipartite graph representing the atlas pair, it may be *partitioned* into multiple connected components in a process that is analogous to noise reduction. For each resulting component, the set of regions in *V_1_* is approximately equivalent to the set of regions in *V_2_*. Examination of the bipartite graphs, at different edge thresholds, provides particularly useful insight into correspondences between two atlases. From Figure 6, we note that, after removing a large number of edges representing overlap of up to 25%, various relationships were revealed (in this case between the H-O and LPBA40 probabilistic atlases). For example, simple correspondences were observed for regions defined in each atlas as the insular cortex, precentral gyrus, or postcentral gyrus. Hierarchical relations were also observed, for example, between the LPBA40 left superior temporal gyrus and six subdivisions of that gyrus provided in the H-O atlas (orange-red component in Figure 6B). Finally, this procedure also revealed significantly more complex relationships consisting of multiply overlapping sets of regions at large spatial scales, e.g. in the frontal regions (red component) and in the posterior portions of the brain (orange component). To the best of our knowledge, no previous methods have been introduced to directly find and visualize such spatially corresponding *region sets*.

As a global measure of the concordance between two parcellations, we used the Adjusted Rand Index (ARI) and a new similarity index developed for this application. To compare these summary indices with “chance” in the given context, we created a series of random parcellations of the test brain matched to each atlas by number of regions, and computed each index repeatedly for pairs of random parcellations. Results for the ARI indicated that only 3 atlas pairs had similarity greater than expected by chance (H-O/ICBM; H-O/LPBA40; ICBM/LPBA40). While the ARI has expected value of zero under the generalized hypergeometric distribution, its computed value was consistently positive in the current context even for random parcellations, indicating a shortcoming in its application to spatially constrained comparisons such as these and indicating the need for understanding empirical chance distributions. The ARI works by comparing the fraction of, in this case, voxel pairs that are either assigned the same label in both parcellations or different labels in both parcellations relative to the total number of voxel pairs. This index does not allow for *refinement* of a single region in one atlas into multiple regions in another without penalty. This may account for some of the low similarity values observed in these comparisons as this type of refinement is observed in numerous places across the different atlases in, for example, the cerebellum (e.g. ICBM vs. H-O), thalamus (e.g. T&G vs. ICBM), and cerebral cortex (e.g. the cingulate cortex in LPBA40 vs. AAL), to name only a few.

The S-index was designed to capture global similarity while allowing for region refinement in one atlas relative to another. It is similar to the local consistency error measure defined by Martin et al. [28] for comparing object segmentations in complex 2D images. The S-index computes a sum of “penalties” for each pair of overlapping regions in the two parcellations, weighted by the relative volume of the smaller region. No penalty is assigned when one region is a pure subset of another (when 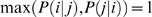; see Figure 2 for illustration), and the largest penalty is assigned when the maximal overlap is 50% (reflecting maximal ambiguity in mapping between the region pair). For regions that overlap only slightly relative to each of their overall volumes, the penalty is accordingly small. Using this index, most atlas pairs were found to be more similar than chance, with the notable exception of *any* atlas compared to *TALc* and most compared to *TALg*. A primary cause of this observed discordance is due to misregistration of the Talairach volume to the MNI-space template brain that can be large relative to the sizes of individual regions in each atlas. The *TALc* parcellation is particularly problematic because it attempts to delineate cytoarchitectonic regions, while other atlases (excepting *CYTO* which is not compared globally) are based on sulcal and/or gyral patterning. Further, because the “cell level” Talairach atlas only delineates a relatively thin cortical strip, which is not well-registered with the test brain cortical surface even after application of a coordinate transform , many of the cortical GM voxels under investigation go unlabeled in TALc. Thus, a comparison of similarity should be *expected* to reveal significant differences given these inconsistencies. Recognizing the problems that misregistration poses for the TALc atlas, and recognizing the ability of the widely-used Talairach Daemon [14] to provide its user with the *nearest* anatomical label for any specified coordinate, we also performed a supplemental analysis on a new parcellation deemed TALc^NH^, which essentially dilates the TALc parcellation in order to provide non-trivial labels to most cortical voxels, as might be performed in assessing activations in an fMRI study; this analysis is provided in Text S2.

### General discussion

We have outlined a key practical problem that impacts the neuroimaging community, and that is illustrative of similar problems throughout neuroscience disciplines. Previous efforts to reconcile different neuroanatomical parcellations and nomenclatures have been mostly limited to the qualitative inference of terms judged by experts to refer to approximately the same segment of brain. Bowden and colleagues have developed a well-known structured nomenclature system for neuroanatomy [5], [6], which consists of a set of hierarchically-related primary and super-structures, a table of *synonymous* terms, and a table of terms for ancillary structures. With the relatively recent rise of the neuroinformatics discipline, several groups have begun to assemble formal machine-readable *ontologies* that encode semantic relationships between neuroanatomical terms [7], [8], [9] in an effort to automate knowledge extraction and facilitate data representation. While these resources are generally useful as controlled vocabularies, they often neglect the fact that the terms are representative of spatially defined entities, and that the most useful mappings between terms will be based on their definitions as such. Additionally, these terminological approaches typically assume that the relationships between anatomical entities can be captured with a small set of possible relationships such as synonymy and parent-child hierarchy. Our results suggest that the mapping problem is considerably more complex, and that incorporating quantitative spatial relations, in the form of conditional probability values, into ongoing ontological efforts, could prove a very fruitful way forward.

One advanced approach to the nomenclature/brain atlas concordance problem was provided by Stephan et al. [31] who developed the objective relational transformation (ORT) method to map between different parcellation schemes in a coordinate-independent manner. This method, used within the *CoCoMac* database (http://www.cocomac.org), relies on defining the *logical relations* between brain areas from different parcellations. In ORT these relations reduce the continuous patterns of spatial overlap between region pairs to a discrete set of possibilities: identity, subset, superset, and partially overlapping. A set of rules is then provided to translate region-level information from parcellation to parcellation. This technique is rigorously developed and is of great interest when only coarse information is available about the relationships between region pairs in different parcellations. Bezgin et al. [27] expanded this framework in a manner similar to our approach in order to *deduce* these logical relationships from spatial partitions. Specifically, they calculated conditional overlap values between regions defined within multiple macaque cortical parcellation schemes represented as surface-based overlays in the Surface Management database system (SumsDB) [26]. Such relationships were extracted by first calculating the conditional overlap values (e.g. *P*(*i|j*), *P*(*j|i*)) for all region pairs, then by classifying each region pair as belonging to one of the possible logical relationships based on the overlap pair. These authors introduced several procedures for performing classification, including a machine learning approach based on previously classified relationships across brain maps. They also made use of a weighting scheme, which allows nodes (cf. voxels) near the centers of regions to have greater influence than those near the boundaries, thereby reducing the impact of potentially imprecise registration of the parcellation schemes. Overall the proposed SORT (Spatial Objective Relational Transformation) approach appears very promising for inferring the necessarily approximate categorical relationships used in the *CoCoMac* system. Nevertheless, the reduction of the computed conditional overlap values to categorical relations is, in some sense, counter-intuitive. By retaining these values as conditional probabilities, indeed it should be possible to replace the complex algebra of ORT with the familiar mathematics of basic probability theory.

It may appear that the problem of translating between multiple parcellation schemes might be simply avoided by referring to the brain geographically with reference to a particular coordinate space, and this is of course done frequently in human neuroimaging studies. Often publications incorporate tables that include lists of coordinates at which particular effects of interest were observed, with reference to one of a small number of commonly-used stereotactic coordinate spaces. Mapping between atlas spaces based on standard coordinate-based data affords superior resolution to the techniques we present here, but coordinate-based approaches are not always feasible, particularly in mining data from the literature. In particular, neither the presentation of coordinate-based results nor the preferred coordinate space is universally agreed upon, and some analyses are performed and reported at the region-level [e.g. 11], [12]. Further, coordinate data almost always provide only a partial view of the results that is invariably supplemented with textual description and anatomical labeling of some form. Related research by Nielsen and Hansen [33] sought to model the relationships between particular spatial coordinates of activations reported in the *BrainMap* functional imaging database [34] and the anatomical labels assigned to these activations by the authors. While their method was designed to detect outliers or errors in database values, it also provides probability density estimates across Talairach space for the “region” corresponding to a given neuroanatomical term. These results can be informative in that they reveal what parts of brain space *have been assigned* a particular label in the database, but they do not take into account the use of different atlases with different definitions of region boundaries.

In general, quantitative treatments of the anatomical parcellation and associated nomenclature problem have been largely absent for several reasons, including the general lack of appropriate methods and of directly comparable digital atlases. In human brain imaging, digital atlases are widely available and easily subjected to mathematical analysis, thus making the present study possible. The high-resolution ICBM template brain was a natural choice as a test brain because it is used in several common software packages and has been examined in some detail, resulting in the availability of the AAL and ICBM anatomical parcellations. The other methods studies, while not specific parcellations of the test brain, could be applied in a manner consistent with common practices in the field. While the results presented here and made available in detail as a web tool should provide valuable information to neuroimaging researchers, the analysis will need to be extended to multiple different brains in order to produce accurate *meta-atlases* that probabilistically map between different parcellations with known measures of uncertainty across individuals. It should be noted that additional efforts are currently under way to provide unified surface and volume-based representations of multiple atlases and anatomical parcellations for human and for macaque [25]. Such efforts will prove very useful in comparing results across studies and in comparing results from neuroimaging with those from “classical” neuroscientific investigations.

To further our overall understanding of the different anatomical labeling conventions currently in use, it would be valuable to establish a common set of MR scans that could be labeled manually by anatomists or otherwise using different parcellation schemes. Manual parcellations are time-consuming and require extensive training to perform but could provide a valuable resource to the community and could additionally be used to improve automated parcellation tools [e.g. 22], [23], [24]. The Open Access Series of Imaging Studies (OASIS) project has made hundreds of structural MR scans freely available to the community [35]; a subset of these scans, for example, could be made available as a parcellation test bed to enable such a project. Additionally these methods are suitable for the comparison of atlases in other species, where researchers also must confront the brain atlas concordance problem. In the rodent, for example, at least two major atlases [36], [37] offer fine parcellations of the rat brain whose boundaries can be quite different over large regions [38], but which have not yet been systematically compared.

### Summary

In this study we have attempted to precisely quantify the spatial relationships between different parcellations of the same human brain anatomy. Our analyses indicate that mapping results from one reference atlas to another is a complex challenge that must be addressed in order to fully comprehend the growing body of literature in functional brain imaging and other neuroscientific disciplines. The problem is particularly crucial for the future success of neuroinformatics initiatives based on automated or semi-automated text analysis. Furthermore, simple ontological efforts based on, for example, synonymy and parent-child relationships, appear to be incapable of capturing the rich landscape of spatial relations observed in this analysis of human brain atlases. Future efforts should thus include a focus on quantitative spatial comparisons of different atlases and parcellations, as presented here (and also in [27], [39]). The framework we have provided appears suitable for the future development of a well-defined meta-atlas to allow probabilistic mapping between labeled regions defined using the myriad protocols available to the neuroscience community today.

## Materials and Methods

### The single-subject ICBM template brain

The high-resolution single subject anatomical template (“Colin27”; [40]) from the International Consortium for Brain Mapping (ICBM) served as the test data for this study. This low-noise template is an intensity average of 27 coregistered T1-weighted gradient-echo MR scans (TR = 18 ms, TE = 10 ms, flip angle = 30°) obtained from the same human subject. The volume has dimensions 181×217×181, with 1 mm isotropic voxels, and covers the entire brain. This single-subject template is provided spatially registered (following application of a 9-parameter global affine transformation) to the commonly used Montreal Neurological Institute (MNI-305) stereotactic coordinate space. The high signal to noise ratio allows one to visually resolve anatomical details not readily seen in a single typical MR scan. This volume is widely available and is distributed with multiple functional imaging software tools.

Voxels from this template brain were assigned probabilities of belonging to one of three tissue types–grey matter (GM), white matter (WM), or cerebrospinal fluid (CSF)–using the Statistical Parametric Mapping (SPM5) software package [41]. Voxels in the *left hemisphere* (both cortical and subcortical) for which the GM probability was greater than 0.25 were included in a binary mask, indicating areas to be labeled using the systems described below. Only the left hemisphere was used because some atlases differentiate between the left and right hemisphere instances of a particular region while others do not.

### Parcellations and atlases

Several different procedures were used to create a set of distinct anatomical parcellations of the test brain. While other atlases and/or protocols for manual parcellation have been described in the literature, some procedures required resources such as expert anatomists trained with a particular protocol that were not available for the present investigation. Table 1 summarizes the 8 procedures used and some of their attributes.

#### Talairach

Two separate parcellations were created based on the Talairach anatomical atlas [15]. The first, *TALg*, is a parcellation into gyri and other macroscopic subdivisions, while the second, *TALc*, is a parcellation into subcortical nuclei (with considerable detail in the thalamus) and architectonic regions, specifically Brodmann's areas [42]. The original Talairach atlas was published in print and labeled sections from a single hemisphere of the cadaver of a 60-year old woman. This atlas became the *de facto* standard in early imaging research because it established a common (although unrepresentative) coordinate space and template. Although the atlas contained Brodmann area labels, it is important to note that no histology was performed, that these labels were determined based simply on visual comparison with Brodmann's illustrations, and that no precise area boundaries were drawn. The atlas was digitized and manually traced to create the Talairach Daemon [14], an online tool that allows researchers to query for labels at five different “levels” at any given point in Talairach space.

Because the single subject test brain is provided aligned to the MNI stereotactic space, which has measurable differences from the Talairach space, a coordinate transformation was used to apply labels from the Talairach atlas to the MNI-space brain. Such mapping between these two template spaces is common in neuroimaging research, and several methods have been prescribed to make the transformation [25], [43], [44], [45]. Here we used the method described by Lancaster et al. [45], specifically the *icbm_spm2tal* transform (http://brainmap.org/icbm2tal/index.html), to map each point in the target brain to a corresponding point in the Talairach atlas, where the labels were simply “read off.” The gyrus-level labels were used to provide *TALg*, and the cell-level labels were used to provide *TALc*. It should be noted that the *TALg* parcellation specifies larger regions than the *TALc* parcellation. This is because regions from *TALg* include portions of the white matter from the Talairach brain, whereas regions from *TALc* do not; see Lancaster et al. (2000) for further details. This problem is partially alleviated here because only voxels likely to constitute GM are considered in the analyses, thus discarding many WM voxels assigned labels by the Talairach Daemon. However, from Table 1 it is clear that misregistration of the thin cortical contours defined in *TALc* with the test brain GM resulted in a low fraction of GM voxels receiving non-trivial labels. In using the Talairach Daemon, many researchers invoke functionality that allows the user to find the *nearest* label to a given input coordinate. We leveraged this idea to create an additional parcellation, called *TALc^NH^*, which is examined in Text S2. In *TALc^NH^*, each unlabled GM voxel in *TALc* is assigned the nearest non-trivial label, assuming one is present within 5 mm of the target voxel.

#### AAL

Because of inaccuracies in using the Talairach atlas to label brains registered to MNI-space, Tzourio-Mazoyer et al. [1] created a new MNI-space anatomical atlas. The single-subject template brain (the test data in the present study) was manually parcellated according to a set of rules based primarily on identifying macro-anatomical landmarks (e.g. prominent sulci), and often with reference to previous delineations. A detailed parcellation of the cerebellum [46] was incorporated, while other subcortical structures (e.g. thalamus) were largely defined as large macro-regions. Cortical regions were drawn not to strictly follow the GM in the subject brain, but also to account for some expected inter-subject variability, by extending into the WM. The Automated Anatomical Labeling (AAL) toolbox is an extension for SPM that makes this atlas available to users, for example, to provide anatomical labels corresponding to locations of activation foci in functional imaging studies. The basic procedure is to simply register MR scans to the MNI-space and “read off” the label from the single subject atlas at one or more voxels of interest. Here the brain being labeled *is* the atlas brain, so no additional steps were necessary. We refer to this parcellation here as *AAL*.

#### ICBM

An additional macro-anatomical parcellation specific to the single-subject test brain is available from the International Consortium for Brain Mapping (http://www.loni.ucla.edu/ICBM/Downloads/Downloads_ICBMtemplate.shtml). Cortical gyri, subcortical structures, and the cerebellum (as a singular entity) are given unique labels. Thalamic and brainstem structures are delineated in considerable detail. We refer to this parcellation as *ICBM*.

#### Tourville and Guenther using FreeSurfer

Tourville and Guenther [47] have developed a landmark-based protocol for parcellation that builds upon a system used at the Center for Morphometric Analysis (CMA) [18] and that is focused in particular on cortical areas involved in speech processing. This system, therefore, incorporates a large number of auditory, motor, and premotor cortical areas. The FreeSurfer software program (http://surfer.nmr.mgh.harvard.edu) was trained to perform automatic cortical parcellation [22] based on a training set of manually labeled scans using this protocol. The test brain was then parcellated using FreeSurfer, and manually edited by an expert anatomist to correct any apparent gross mislabelings. Freesurfer's default subcortical parcellation [48] was also performed, and the sum results of both cortical and subcortical labeling were projected back into the original volume-space, resulting in a parcellation (*T&G*) that could be readily compared to those described above.

#### Probabilistic atlases

Three probabilistic brain atlases were also used to parcellate the test brain. Such atlases give an estimate of the probability that a given voxel in a standard space belongs to a particular region. Probability estimates are based on the proportion of voxels at a given location in a set of individual manually labeled brains registered to the template space that have been assigned any given label. For each probabilistic atlas used here, deterministic parcellations were created by assigning the most probable region label to each selected voxel. The *CYTO* parcellation was created using the probabilistic cytoarchitectonic maps from Zilles and colleagues, published as the *Anatomy Toolbox* [49]. These maps, which are derived from post-mortem histological analysis in multiple subjects, then registered to the MNI-space using a high-dimensional non-linear registration algorithm, do not cover the entire brain; for this reason they are excluded from certain comparisons, including global atlas similarity described below. Notably, CYTO is the only labeling method examined that is based on direct histological investigations. The second probabilistic atlas, the Harvard-Oxford (*H-O*) atlas (distributed with the FSL software package; http://fsl.fmrib.ox.ac.uk/fsl/), was created by affine-registering 37 individual scans that were each manually parcellated according to the CMA protocols [18] to MNI-space using the *FLIRT* tool in FSL. For the present study, the cortical and subcortical atlases distributed with FSL were combined into a single volume. The *H-O* parcellation is again the maximum likelihood labeling at each voxel. Lastly, the LONI probabilistic brain atlas (*LPBA40*) [50] consists of 40 manually labeled brains according to a set of protocols developed at UCLA's Laboratory of Neuroimaging. The atlas has several variants based on the choice of methods used to register the individually labeled brains. Here we used a maximum likelihood parcellation created from a version of the atlas that used the SPM5 default registration methods to align each scan to the template space.

Each of the 8 parcellations above assumed the final form of a 181×217×181 voxel volume. Each left hemisphere GM voxel was labeled in each parcellation as belonging to a particular area or to “none.” Formally, this resulted in each of the *M* relevant voxels *x_i_*, 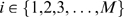 in the image being mapped to a non-negative integer label corresponding to a particular region, i.e. 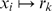. Unlabeled voxels were assigned the value 0. A subset of the voxels in these volumes was subjected to analysis. These voxels were selected by i) finding the union of all voxels assigned a non-zero label in *any* of the atlases considered, and ii) intersecting that voxel set with the left hemisphere GM mask described above.

### Region-level concordance matrices

The parcellations of the test brain can be mathematically formalized as sets or sets of sets. Specifically, a single parcellation *R* is a set of *N* regions,
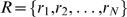
(1)and each region comprises the set of indices of the voxels that map to the same anatomical label:
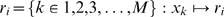
(2)


We define a non-symmetric measure of spatial overlap between a region *i* from parcellation *R* and region *j* from parcellation *R′* as:
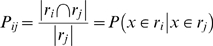
(3)where |*r_i_*| indicates set cardinality, or the number of voxels in the region or intersection of regions. *P_ij_* thus indicates the proportion of region *r_j_* that is contained within the bounds of region *r_i_*. Its values are limited to the interval [0,1], and thus *P_ij_* has a straight-forward interpretation as the conditional probability that a voxel is contained in region *i* given that it is contained in region *j*, averaged across all voxels in *r_j_*. For simplicity, we write these conditionals as 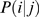 or simply *P_ij_*, omitting the reference to voxel *x*.

This conditional measure is to be contrasted with more commonly employed symmetric overlap measures, such as the Dice coefficient [51] or the Jaccard similarity index [52], which can only take its maximum value of 1 when the regions are *identically* defined. *P_ij_* is instead 1 when there is a pure subset relationship, even when *P_ji_<*1. We also employed a symmetric index of overlap, but one that is readily computed from the *P_ij_* values and whose denominator is the geometric mean rather than size of the union of the two regions:
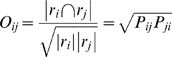
(4)


This index, which is equivalent to the *cosine coefficient* for binary vectors as commonly used in information retrieval [53] , again takes values on [0,1], but is only equal to 1 when the two regions are identically defined. Here *O_ij_ = O_ji_*.

The above measures are each defined with respect to two parcellations. For convenience we concatenated the sets of regions in all atlases into a single list, such that each region is indexed with a unique integer. This resulted in matrices *P* and *O* being square *block matrices* where the individual blocks indicated the spatial relationships between the regions from two individual atlases. For non-symmetric matrix *P*, two blocks–one in the lower triangular portion and one in the upper triangular portion–are necessary to capture the conditional probabilities related to the comparison of any two atlases. From the *P* and *O* matrices one can readily calculate various statistical properties across parcellations (e.g. the number of other regions that any particular region overlaps), and these can form the basis of more advanced analyses and visualization procedures.

#### Visualization of region-level results

The order of the regions encompassed in each parcellation was initially arbitrary. The indices may be reordered algorithmically in order to improve visualization and interpretability of global results. A method was developed to permute the rows and columns of *each block* in the block matrix *P* independently in order to reduce the matrix bandwidth (defined as 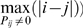) for each block. This has the effect of reordering the regions in pairs of parcellations to reflect similar patterns across brain space. Because bandwidth minimization is an NP-complete problem [54], we used a heuristic that proceeds as follows: i) the singular value decomposition (SVD) is computed for each block of the matrix *P*; ii) the first left and right singular vectors are sorted, and the sort indices are used as permutations of the rows and columns within the block. This results in the non-zero entries in each block being permuted toward the diagonal, yielding a more suitable visualization than the arbitrarily ordered *P*.

Non-metric multi-dimensional scaling (MDS) was also used to visualize region labels from all atlases in two-dimensional space. The input to MDS was a symmetric dissimilarity matrix 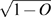 calculated from the symmetric overlap matrix. The *mdscale* routine from the MATLAB Statistics Toolbox was used, which uses Kruskal's normalized STRESS1 criterion [55] for optimization; this method returns *x-* and *y-* coordinates for each region such that the distances between points is approximately monotonically related to the dissimilarity values.

#### Extracting hierarchical relationships through graph partitioning

The spatial relationships between regions in two different atlases can be complex. Inferring higher-order relationships is difficult due to the vast combinatorial possibilities. We addressed this problem using a graph theoretical approach. For any pair of parcellations, we define a weighted *bipartite graph B = *(*V_1_+V_2_*, *E*) where edges *E* are weighted as:
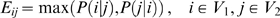
(5)


Here *V_1_* and *V_2_* are distinct vertex sets representing the sets of regions in each of the two parcellations. For the present parcellations, this graph is typically connected (there is a path from any node to any other). Various graph partitioning methods can be employed to cut the graph into multiple components. Here we employed a very simple algorithm, which iteratively removes the edge with smallest non-zero weight until a threshold for the maximum number of graph components or maximum pruned edge weight is reached. We then deduced that, for each resulting connected component, the union of regions represented in *V_1_* in that component has a spatial correspondence with the union of regions represented in *V_2_*, up to some level of “noise” determined by the stopping criterion. Intuitively, this indicates that some set of regions in one parcellation has high overlap, in potentially complex patterns, with some set of regions in another parcellation. For visualization of the bipartite graph, the vertices in *V_1_* and *V_2_* were reordered using techniques from spectral graph theory [56]. Nodes were re-indexed by sorting the Fiedler vector (the eigenvector corresponding to the second smallest eigenvalue of the graph Laplacian) [57]; this re-indexing brought the connected components in closer proximity within the graph layout and minimized the visualization problem of crossing edges.

#### Global similarity of parcellations

To address the global similarity or concordance of two parcellations we first applied the Adjusted Rand Index (ARI; [32]). The ARI computes the fraction of all possible *pairs* of voxels that are either i) in the same region in both parcellations or ii) in different regions in both parcellations, and is normalized such that its expected value is 0 under the generalized hypergeometric model of randomness. The ARI formulated here for comparing parcellations is:
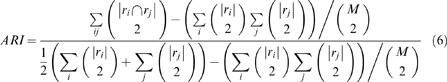
(6)where *r_i_* and *r_j_* are the sets of voxels labeled as region *i* in the first parcellation and region *j* in the second, respectively, and *M* is the total number of labeled voxels. The notation 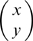 denotes “x choose y” or the number of ways *y* items can be chosen from a set of *x*.

Additionally, we developed a new index, *S*, which is calculated from the *P_ij_* values for a pair of parcellations. The index “penalizes” region-to-region relationships that are overlapping, but that are not pure subset relationships. The maximum penalty for a pair of regions occurs when one region overlaps another by exactly 50% of its volume. These pair-wise “penalties” are weighted by the size of the regions involved. The scalar-valued similarity index *S* for two parcellations, which takes values between 0 and 1, was computed as follows. First the maximum of the two conditional probabilities corresponding to each region pair was calculated,
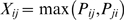
along with “weights” for each non-zero *X_ij_* corresponding to the relative volume of the smaller region:
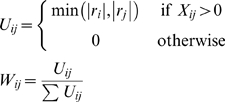



Then the weighted maximum conditional probabilities were combined and subtracted from 1 as penalty terms in order to arrive at the final expression for global concordance:
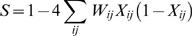
(7)


#### Random parcellations

The pair-wise global similarity values for different parcellations have little meaning without an understanding of their expected values in the given context. To this end, we developed the notion of a *random parcellation* of the template scan. Space-filling random parcellations of the scan into *N* contiguous regions were created as follows:

1. *N* “seed points” were chosen randomly from voxels 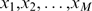 and assigned region labels 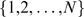.
2. For each labeled voxel *x_i_*, its 6-neighborhood in three dimensions was calculated, and all *unlabeled* neighboring voxels were assigned label *L*(*x_i_*).
3. Step 2 was repeated until all voxels 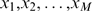 were labeled.

A matched set of random parcellations was created for each atlas; that is, the number of regions in the random parcellations was set equal to the number of regions in a given atlas. Global similarity was assessed between pairings of random parcellations in order to establish the distributions of “chance” concordance when comparing arbitrary partitions of brain space.

## Supporting Information

Figure S1 Symmetric concordance matrix. Region-level concordance results across eight parcellations using the symmetric measure Oij = sqrt(Pij*Pji). Each row and column corresponds to a particular anatomical region, and regions are grouped by parcellation method (separated by gray horizontal and vertical lines). Non-zero (non-black) entries indicate some degree of overlap between the region pair. Only the upper diagonal elements are shown because of symmetry.
(0.45 MB TIF)

Text S1 Bipartite graph comparisons of anatomical parcellations. Bipartite graph comparisons of anatomical parcellations. Each pair of parcellations is compared using the bipartite graph formulation described in our paper. The graphs are shown for theta = 0.10 and for theta = 0.25.
(0.71 MB PDF)

Text S2 Supplemental analysis based on TALcNH parcellation.
(0.29 MB PDF)

Table S1 Global concordance measures for the comparison of parcellations computed using voxels in i) a cerebral cortex only mask , and ii) a subcortical only mask.
(0.03 MB PDF)

## References

[1] Tzourio-Mazoyer N, Landeau B, Papathanassiou D, Crivello F, Etard O (2002). Automated anatomical labeling of activations in SPM using a macroscopic anatomical parcellation of the MNI MRI single-subject brain.. Neuroimage.

[2] Wilder BG (1896). Neural terms, international and national.. Journal of Comparative Neurology.

[3] Whitmore I (1998). Terminologia anatomica: international anatomical terminology: Thieme Publishing Group.

[4] Anthoney TR (1994). Neuroanatomy and the Neurologic Exam: A Thesaurus of Synonyms, Similar-Sounding Non-Synonyms, and Terms of Variable Meaning: CRC Press.

[5] Bowden DM, Dubach MF (2003). NeuroNames 2002.. Neuroinformatics.

[6] Bowden DM, Martin RF (1995). NeuroNames Brain Hierarchy.. Neuroimage.

[7] Lindberg DA, Humphreys BL, McCray AT (1993). The Unified Medical Language System.. Methods Inf Med.

[8] Rosse C, Mejino JL (2003). A reference ontology for biomedical informatics: the Foundational Model of Anatomy.. J Biomed Inform.

[9] Rubin DL, Lewis SE, Mungall CJ, Misra S, Westerfield M (2006). National Center for Biomedical Ontology: advancing biomedicine through structured organization of scientific knowledge.. Omics.

[10] Kennedy DN, Lange N, Makris N, Bates J, Meyer J (1998). Gyri of the human neocortex: an MRI-based analysis of volume and variance.. Cereb Cortex.

[11] Nieto-Castanon A, Ghosh SS, Tourville JA, Guenther FH (2003). Region of interest based analysis of functional imaging data.. Neuroimage.

[12] Poldrack RA (2007). Region of interest analysis for fMRI.. Soc Cogn Affect Neurosci.

[13] Rademacher J, Galaburda AM, Kennedy DN, Filipek PA, Caviness VS (1992). Human cerebral cortex: localization, parcellation, and morphometry with magnetic resonance imaging.. Journal of cognitive neuroscience.

[14] Lancaster JL, Woldorff MG, Parsons LM, Liotti M, Freitas CS (2000). Automated Talairach atlas labels for functional brain mapping.. Hum Brain Mapp.

[15] Talairach J, Tournoux P (1988). Co-planar stereotaxic atlas of the human brain..

[16] Brett M, Johnsrude IS, Owen AM (2002). The problem of functional localization in the human brain.. Nat Rev Neurosci.

[17] Devlin JT, Poldrack RA (2007). In praise of tedious anatomy.. Neuroimage.

[18] Caviness VS, Meyer J, Makris N, Kennedy DN (1996). MRI-based topographic parcellation of human neocortex: an anatomically specified method with estimate of reliability.. Journal of Cognitive Neuroscience.

[19] Crespo-Facorro B, Kim JJ, Andreasen NC, O'Leary DS, Wiser AK (1999). Human frontal cortex: an MRI-based parcellation method.. Neuroimage.

[20] Hammers A, Allom R, Koepp MJ, Free SL, Myers R (2003). Three-dimensional maximum probability atlas of the human brain, with particular reference to the temporal lobe.. Hum Brain Mapp.

[21] Kim JJ, Crespo-Facorro B, Andreasen NC, O'Leary DS, Zhang B (2000). An MRI-based parcellation method for the temporal lobe.. Neuroimage.

[22] Fischl B, van der Kouwe A, Destrieux C, Halgren E, Segonne F (2004). Automatically Parcellating the Human Cerebral Cortex.. Cerebral Cortex.

[23] Klein A, Hirsch J (2005). Mindboggle: a scatterbrained approach to automate brain labeling.. Neuroimage.

[24] Pohl KM, Bouix S, Nakamura M, Rohlfing T, McCarley RW (2007). A hierarchical algorithm for MR brain image parcellation.. IEEE Trans Med Imaging.

[25] Van Essen DC, Dierker DL (2007). Surface-based and probabilistic atlases of primate cerebral cortex.. Neuron.

[26] Van Essen DC, Harwell J, Hanlon D, Dickson J, Koslow SH, Subramaniam S (2005). Surface-based atlases and a database of cortical structure and function.. Databasing the brain: From data to knowledge (Neuroinformatics).

[27] Bezgin G, Wanke E, Krumnack A, Kotter R (2008). Deducing logical relationships between spatially registered cortical parcellations under conditions of uncertainty.. Neural Netw.

[28] Muller HM, Kenny EE, Sternberg PW (2004). Textpresso: an ontology-based information retrieval and extraction system for biological literature.. PLoS Biol.

[29] Burns GA, Cheng WC (2006). Tools for knowledge acquisition within the NeuroScholar system and their application to anatomical tract-tracing data.. J Biomed Discov Collab.

[30] Muller HM, Rangarajan A, Teal TK, Sternberg PW (2008). Textpresso for Neuroscience: Searching the Full Text of Thousands of Neuroscience Research Papers.. Neuroinformatics.

[31] Stephan KE, Zilles K, Kotter R (2000). Coordinate-independent mapping of structural and functional data by objective relational transformation (ORT).. Philos Trans R Soc Lond B Biol Sci.

[32] Hubert L, Arabie P (1985). Comparing partitions.. Journal of Classification.

[33] Nielsen FA, Hansen LK (2002). Modeling of activation data in the BrainMap database: detection of outliers.. Hum Brain Mapp.

[34] Laird AR, Lancaster JL, Fox PT (2005). BrainMap: the social evolution of a human brain mapping database.. Neuroinformatics.

[35] Marcus DS, Wang TH, Parker J, Csernansky JG, Morris JC (2007). Open Access Series of Imaging Studies (OASIS): cross-sectional MRI data in young, middle aged, nondemented, and demented older adults.. J Cogn Neurosci.

[36] Paxinos G, Watson C (2007). The Rat Brain in Stereotaxic Coordinates..

[37] Swanson LW (2003). Brain Maps: Structure of the Rat Brain..

[38] Bota M, Dong HW, Swanson LW (2003). From gene networks to brain networks.. Nat Neurosci.

[39] Bezgin G, Reid AT, Schubert D, Kotter R (2009). Matching spatial with ontological brain regions using Java tools for visualization, database access, and integrated data analysis.. Neuroinformatics.

[40] Holmes CJ, Hoge R, Collins L, Woods R, Toga AW (1998). Enhancement of MR images using registration for signal averaging.. J Comput Assist Tomogr.

[41] Ashburner J, Friston KJ (2005). Unified segmentation.. Neuroimage.

[42] Brodmann K (1909). Vergleichende Lokalisationslehre der Grosshirnrinde in ihren Prinzipien dargestellt auf Grund des Zellenbaues..

[43] Carmack PS, Spence J, Gunst RF, Schucany WR, Woodward WA (2004). Improved agreement between Talairach and MNI coordinate spaces in deep brain regions.. NeuroImage.

[44] Chau W, McIntosh AR (2005). The Talairach coordinate of a point in the MNI space: how to interpret it.. NeuroImage.

[45] Lancaster JL, Tordesillas-Gutierrez D, Martinez M, Salinas F, Evans A (2007). Bias between MNI and Talairach coordinates analyzed using the ICBM-152 brain template.. Hum Brain Mapp.

[46] Schmahmann JD, Doyon J, McDonald D, Holmes C, Lavoie K (1999). Three-dimensional MRI atlas of the human cerebellum in proportional stereotaxic space.. Neuroimage.

[47] Tourville JA, Guenther FH (2003). A cortical parcellation scheme for speech studies.. Boston University Technical Report CAS/CNS-03-022.

[48] Fischl B, Salat DH, Busa E, Albert M, Dieterich M (2002). Whole Brain Segmentation Automated Labeling of Neuroanatomical Structures in the Human Brain.. Neuron.

[49] Eickhoff SB, Stephan KE, Mohlberg H, Grefkes C, Fink GR (2005). A new SPM toolbox for combining probabilistic cytoarchitectonic maps and functional imaging data.. Neuroimage.

[50] Shattuck DW, Mirza M, Adisetiyo V, Hojatkashani C, Salamon G (2008). Construction of a 3D probabilistic atlas of human cortical structures.. Neuroimage.

[51] Dice LR (1945). Measures of the Amount of Ecologic Association Between Species.. Ecology.

[52] Jaccard P (1901). Etude comparative de la distribution florale dans une portion des Alpes et des Jura.. Bull Soc Vaudoise Sci Nat.

[53] Salton G (1989). Automatic text processing: the transformation, analysis, and retrieval of information by computer. Boston, MA: Addison-Wesley Longman Publishing.

[54] Papadimitriou CH (1976). The NP-Completeness of the bandwidth minimization problem.. Computing.

[55] Kruskal J (1964). Nonmetric multidimensional scaling: A numerical method.. Psychometrika.

[56] Chung FRK (1997). Spectral graph theory..

[57] Fiedler M (1973). Algebraic connectivity of graphs.. Czechoslovak Mathematical Journal.

