# Supplementary figures and images for: The Brain Atlas Concordance Problem: Quantitative Comparison of Anatomical Parcellations
Source: PLoS One. 2009 Sep 29;4(9):e7200. doi: 10.1371/journal.pone.0007200 (PMC2748707; doi:10.1371/journal.pone.0007200)

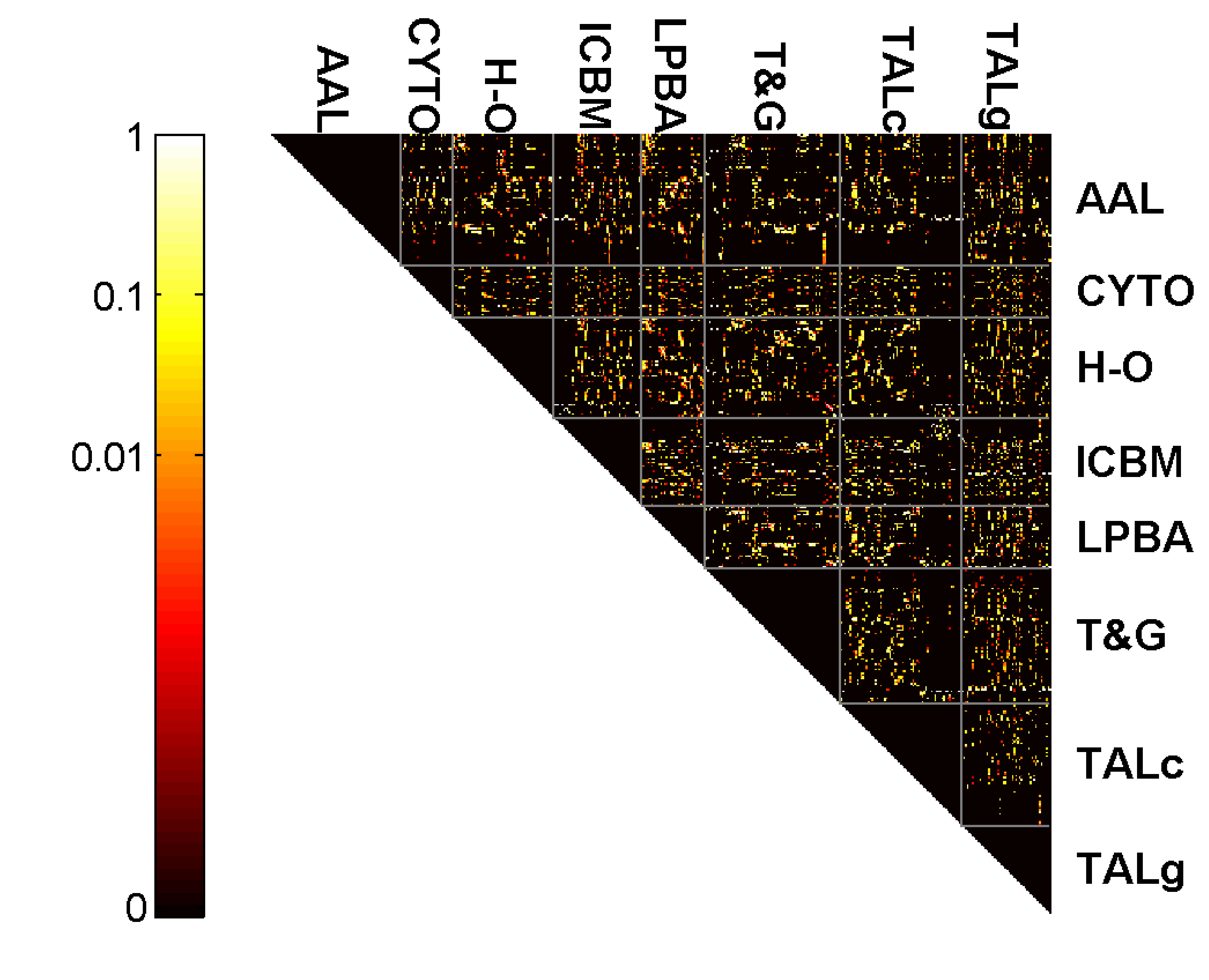

Supplement: Figure S1 — Symmetric concordance matrix. Region-level concordance results across eight parcellations using the symmetric measure Oij = sqrt(Pij*Pji). Each row and column corresponds to a particular anatomical region, and regions are grouped by parcellation method (separated by gray horizontal and vertical lines). Non-zero (non-black) entries indicate some degree of overlap between the region pair. Only the upper diagonal elements are shown because of symmetry. (0.45 MB TIF) [file pone.0007200.s001.tif]
