# Supplementary material for: The Brain Atlas Concordance Problem: Quantitative Comparison of Anatomical Parcellations
Source: PLoS One. 2009 Sep 29;4(9):e7200. doi: 10.1371/journal.pone.0007200 (PMC2748707; doi:10.1371/journal.pone.0007200)
Supplement: Text S1 — Bipartite graph comparisons of anatomical parcellations. Bipartite graph comparisons of anatomical parcellations. Each pair of parcellations is compared using the bipartite graph formulation described in our paper. The graphs are shown for theta = 0.10 and for theta = 0.25. (0.71 MB PDF) [file pone.0007200.s002.pdf]

The brain atlas concordance problem: quantitative comparison of anatomical parcellations
Supporting Text S1

Jason W. Bohland, Hemant Bokil, Cara B. Allen, and Partha P. Mitra

Contents

1 Bipartite graph comparisons of anatomical parcellations **2**
1.1 AAL - CYTO 2
1.2 AAL - H-O 3
1.3 AAL - ICBM 4
1.4 AAL - LPBA 5
1.5 AAL - T&G 6
1.6 AAL - TALc 7
1.7 AAL - TALg 8
1.8 CYTO - H-O 9
1.9 CYTO - ICBM 10
1.10 CYTO - LPBA 11
1.11 CYTO - T&G 12
1.12 CYTO - TALc 13
1.13 CYTO - TALg 14
1.14 H-O - ICBM 15
1.15 H-O - LPBA 16
1.16 H-O - T&G 17
1.17 H-O - TALc 18
1.18 H-O - TALg 19
1.19 ICBM - LPBA 20
1.20 ICBM - T&G 21
1.21 ICBM - TALc 22
1.22 ICBM - TALg 23
1.23 LPBA - T&G 24
1.24 LPBA - TALc 25
1.25 LPBA - TALg 26
1.26 T&G - TALc 27
1.27 T&G - TALg 28
1.28 TALc - TALg 29

1 Bipartite graph comparisons of anatomical parcellations

Each pair of parcellations is compared using the bipartite graph formulation described in our paper. The graphs are shown for $\theta = 0.10$ and for $\theta = 0.25$.

1.1 AAL - CYTO

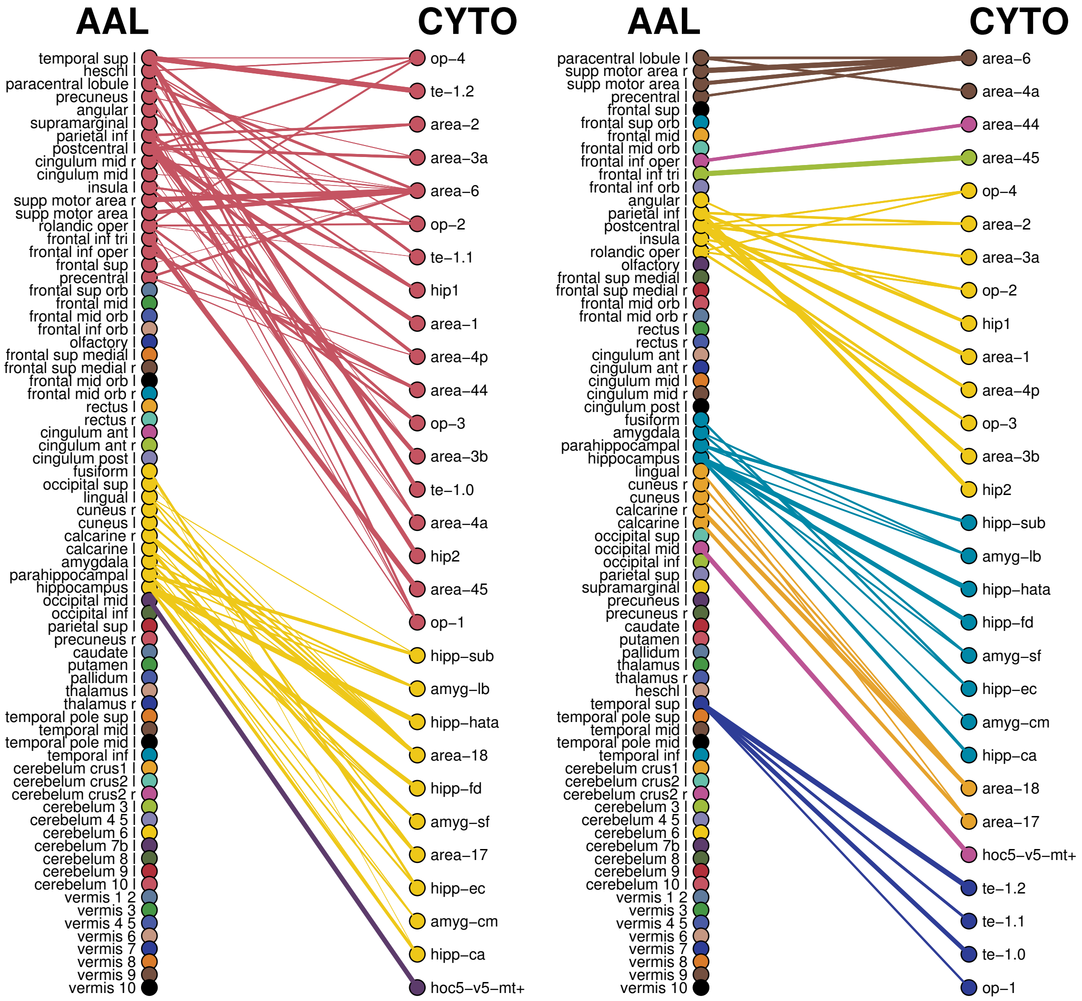

Figure 1: Left: Edges pruned up to $\theta = 0.10$; Right: Edges pruned up to $\theta = 0.25$.

1.2 AAL - H-O

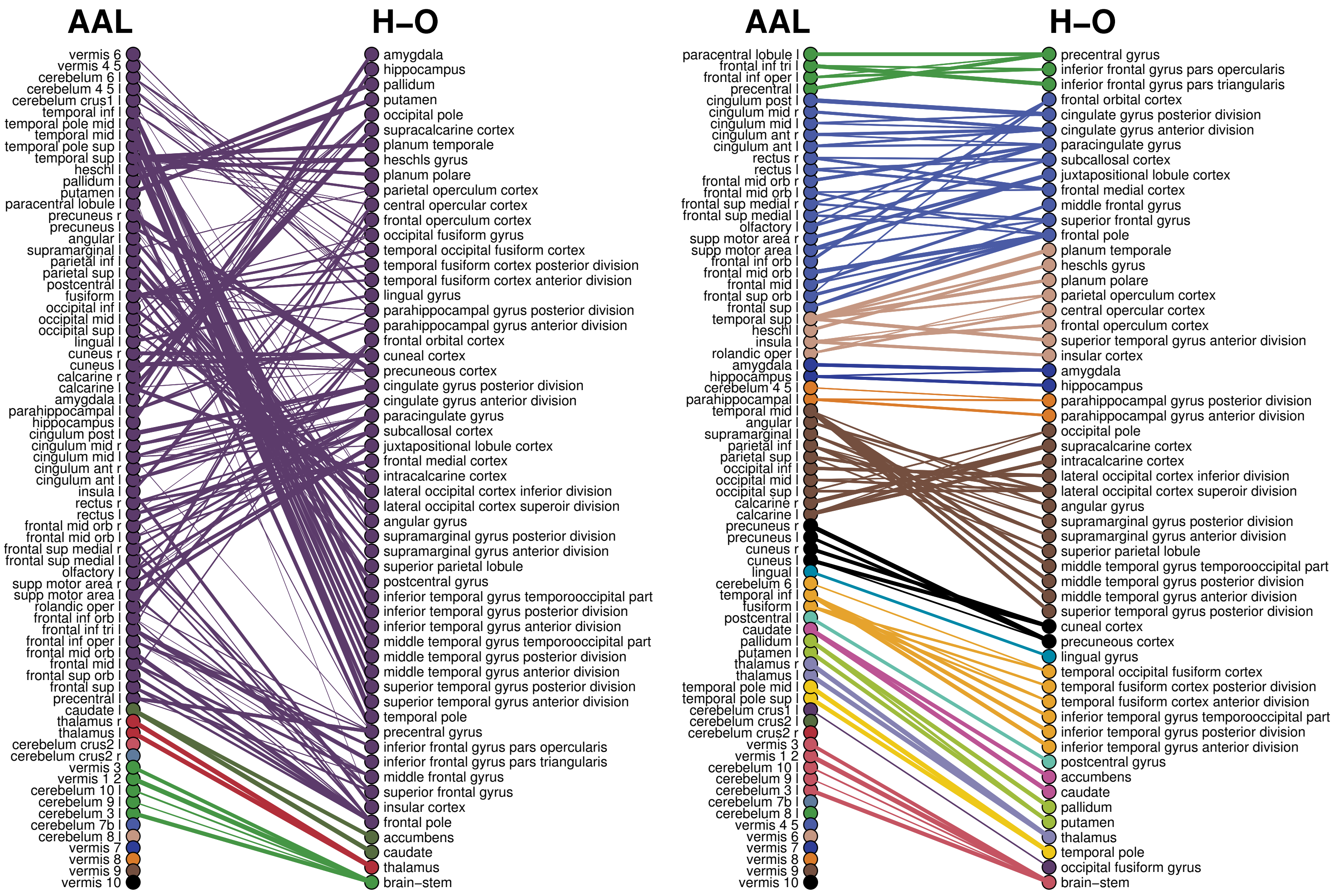

Figure 2: Left: Edges pruned up to $\theta = 0.10$; Right: Edges pruned up to $\theta = 0.25$.

1.3 AAL - ICBM

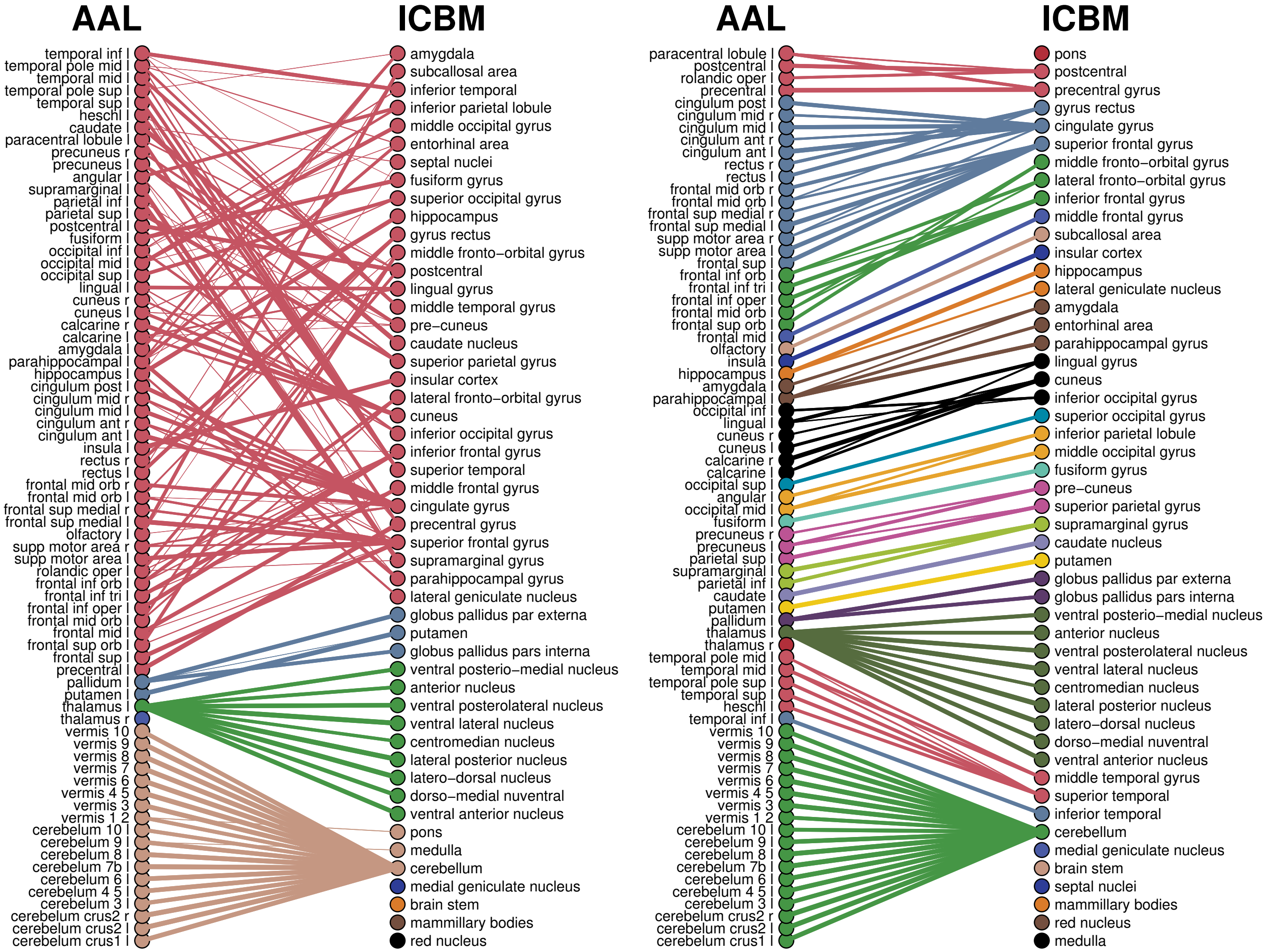

Figure 3: Left: Edges pruned up to $\theta = 0.10$; Right: Edges pruned up to $\theta = 0.25$.

1.4 AAL - LPBA

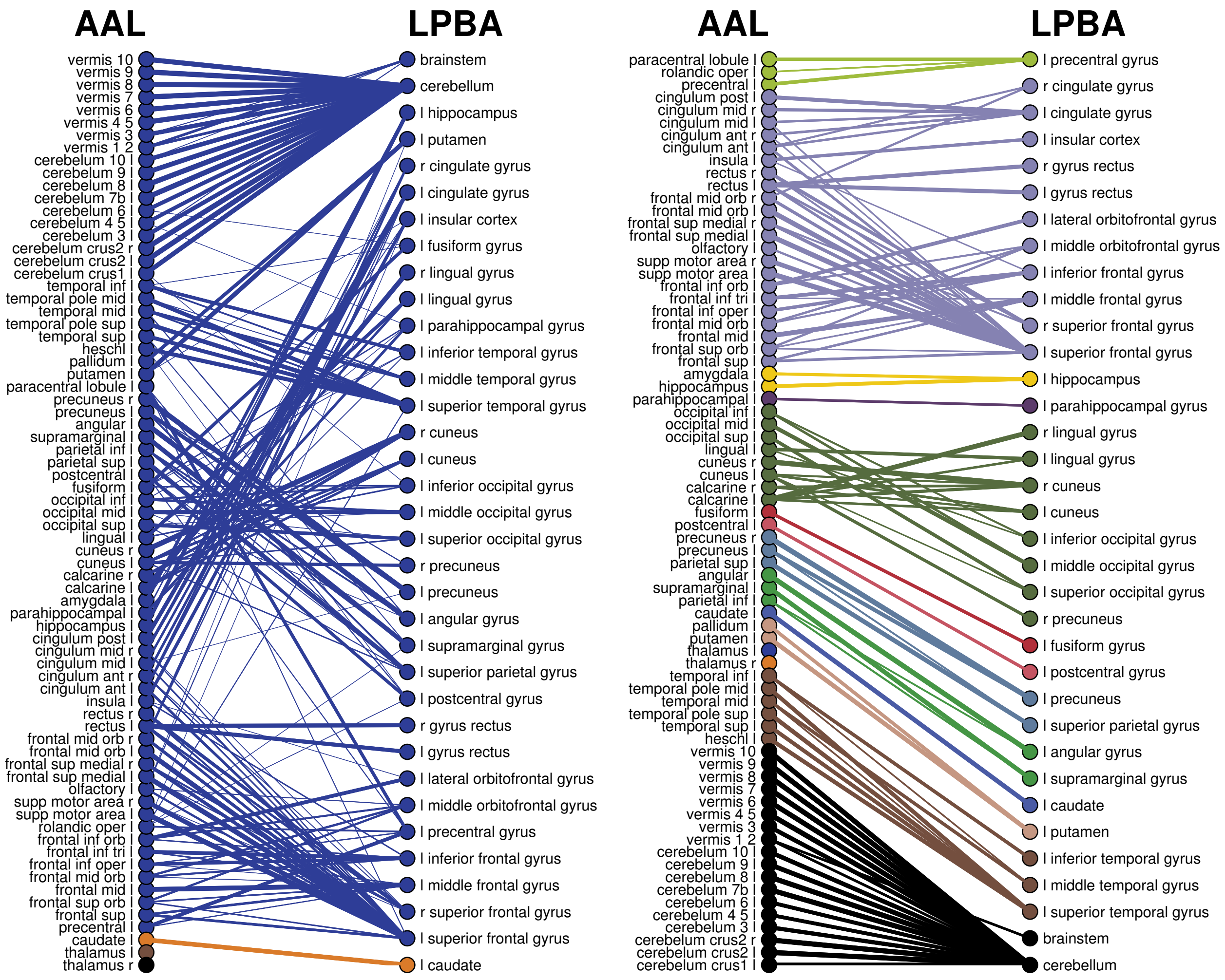

Figure 4: Left: Edges pruned up to $\theta = 0.10$; Right: Edges pruned up to $\theta = 0.25$.

1.5 AAL - T&G

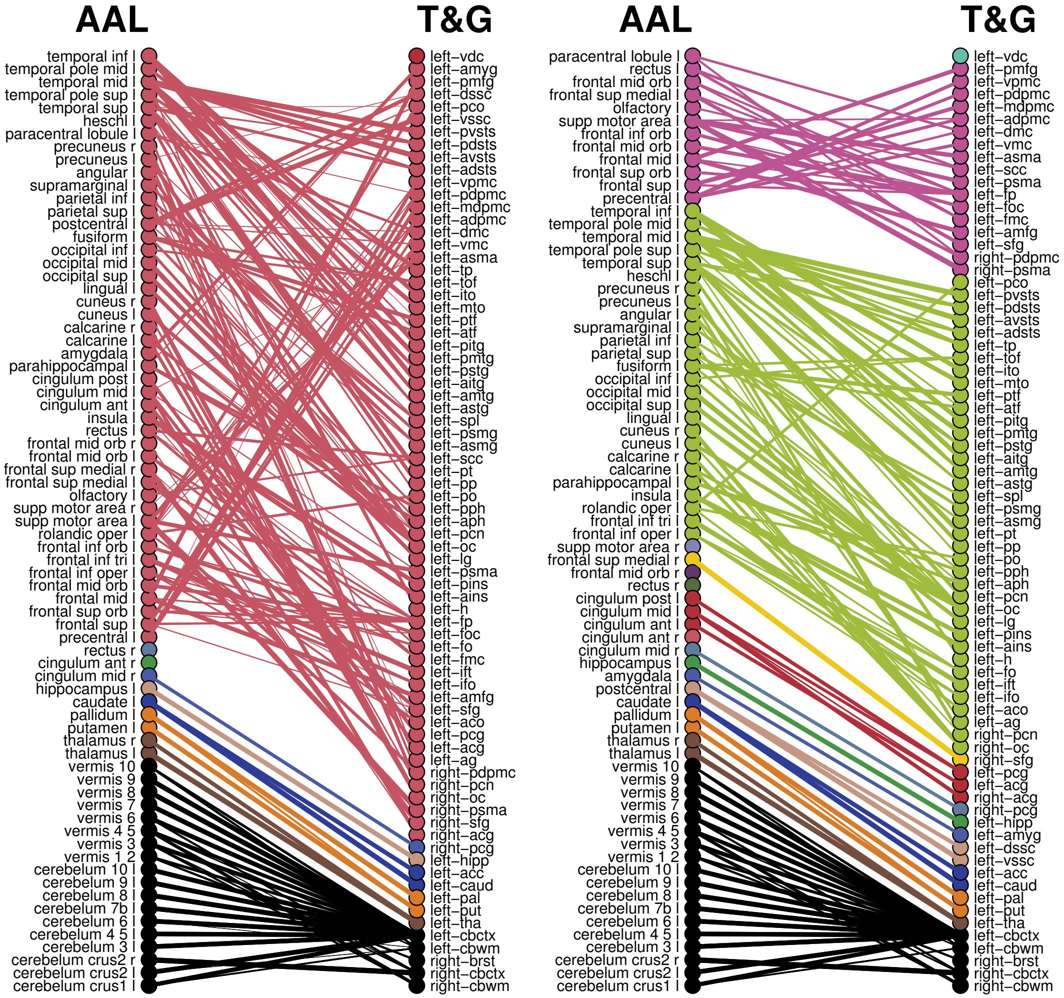

Figure 5: Left: Edges pruned up to $\theta = 0.10$; Right: Edges pruned up to $\theta = 0.25$.

1.6 AAL - TALc

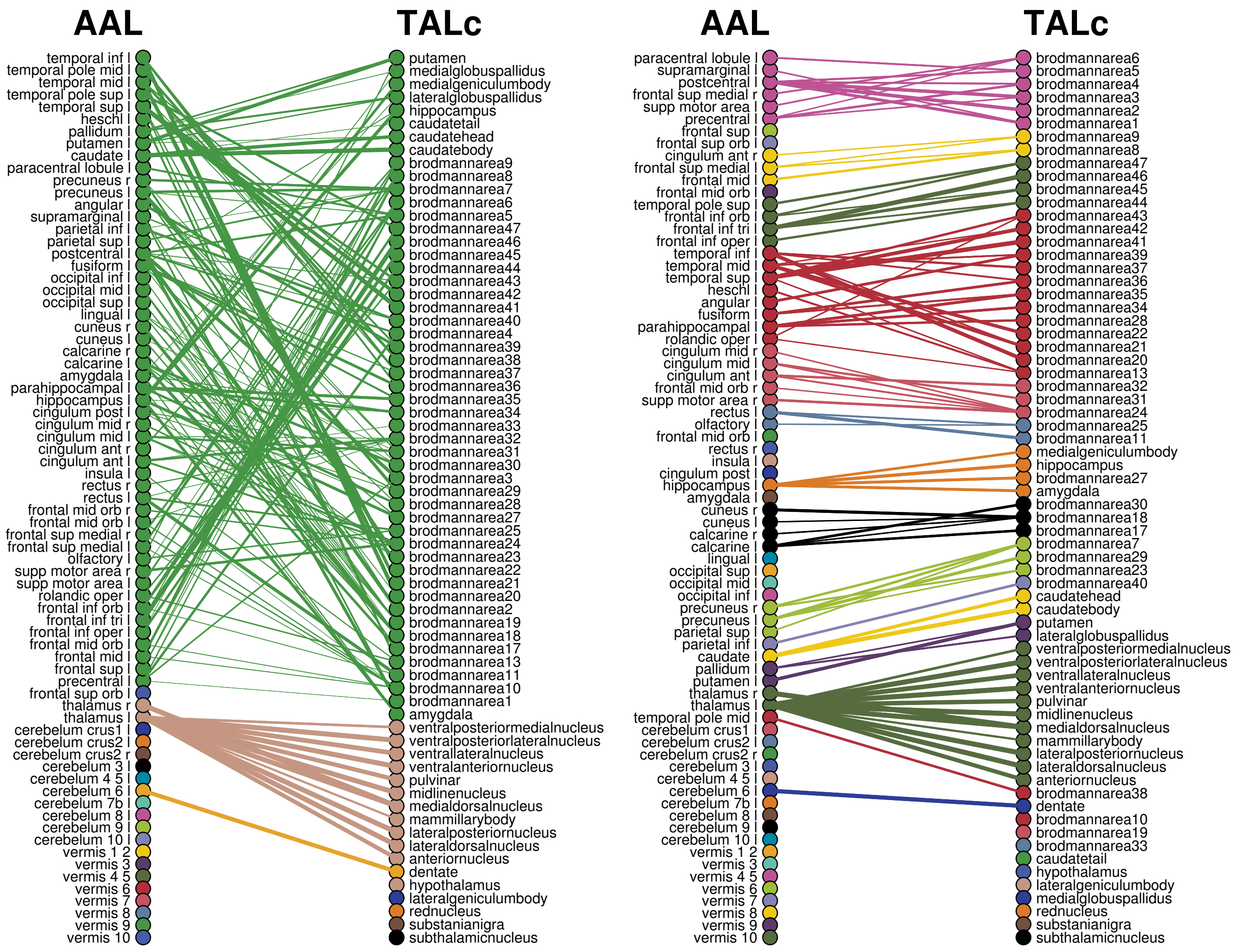

Figure 6: Left: Edges pruned up to $\theta = 0.10$; Right: Edges pruned up to $\theta = 0.25$.

1.7 AAL - TALg

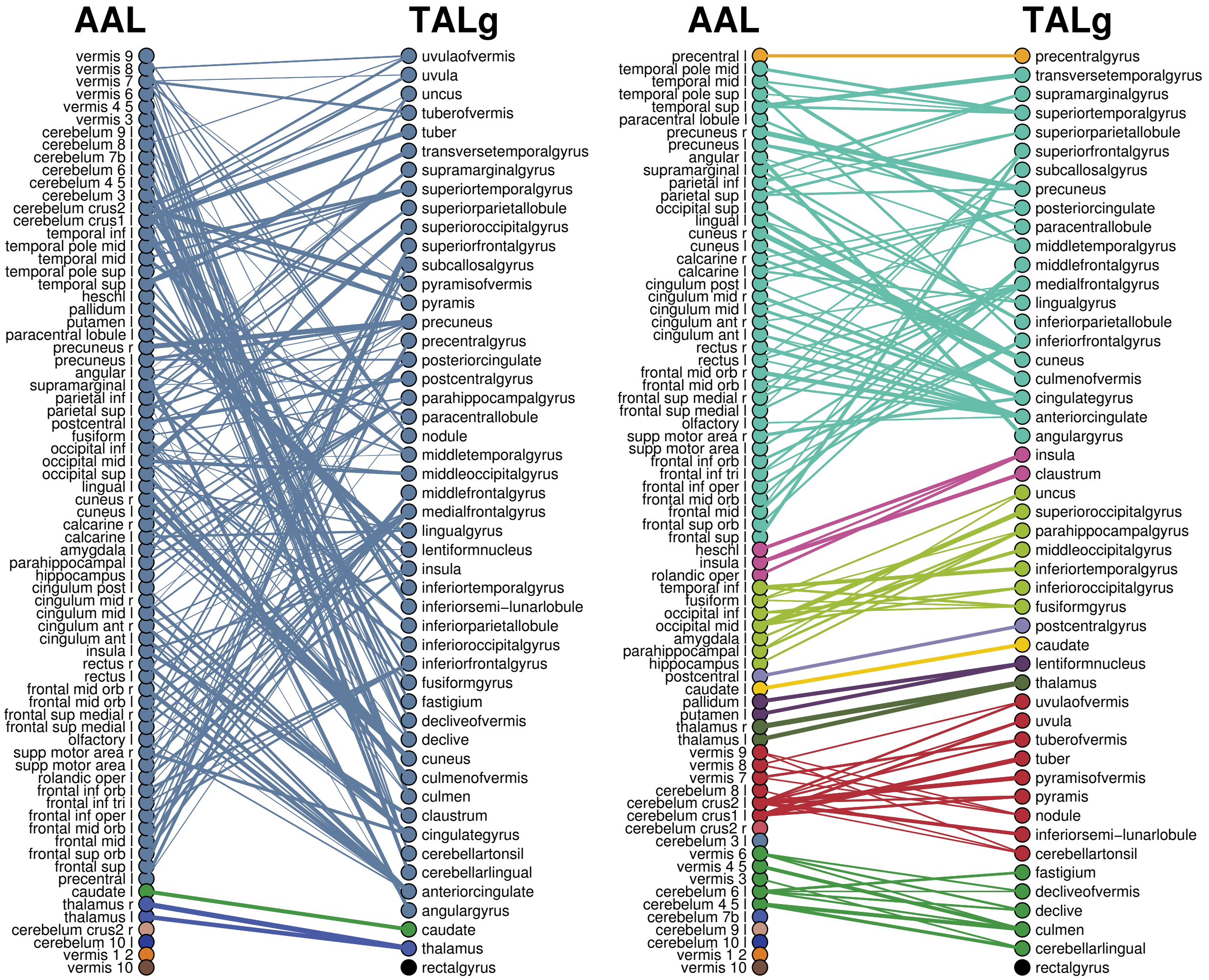

Figure 7: Left: Edges pruned up to $\theta = 0.10$; Right: Edges pruned up to $\theta = 0.25$.

1.8 CYTO - H-O

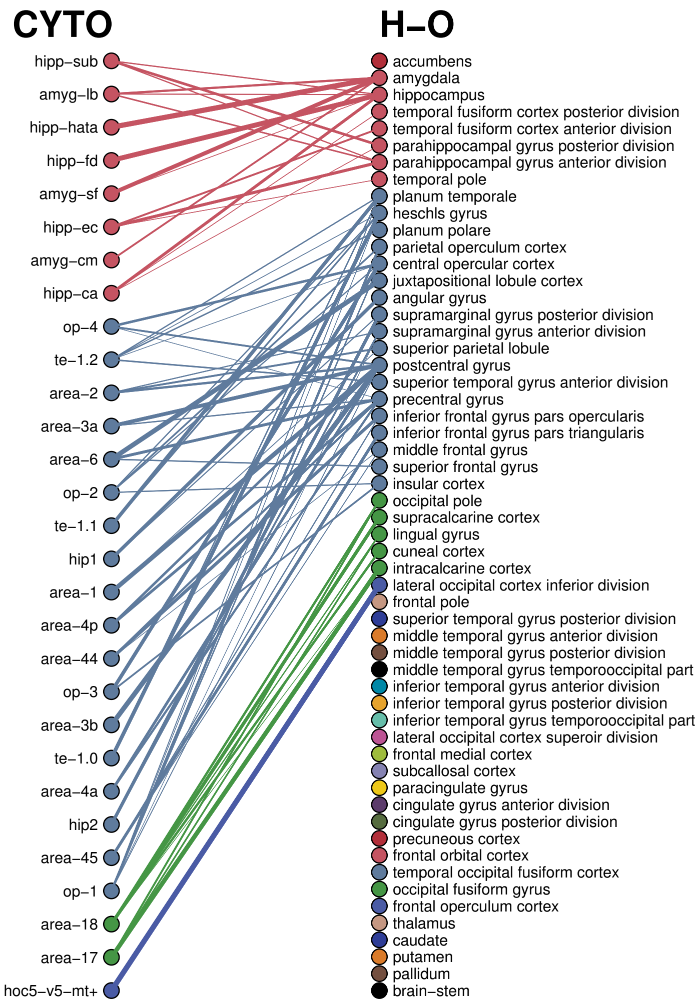

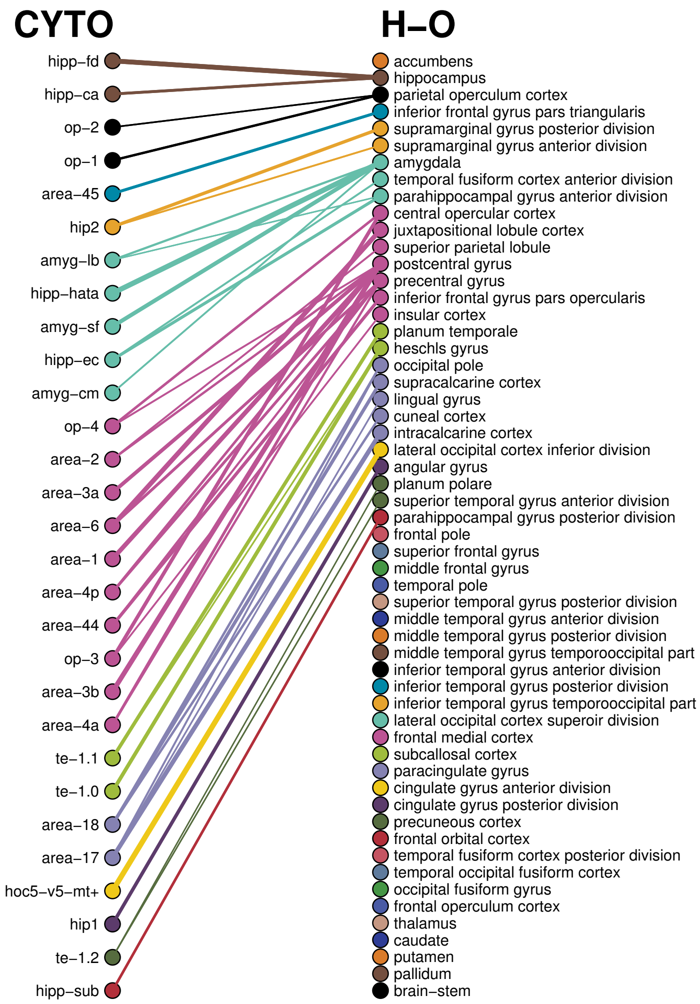

Figure 8: Left: Edges pruned up to $\theta = 0.10$; Right: Edges pruned up to $\theta = 0.25$.

1.9 CYTO - ICBM

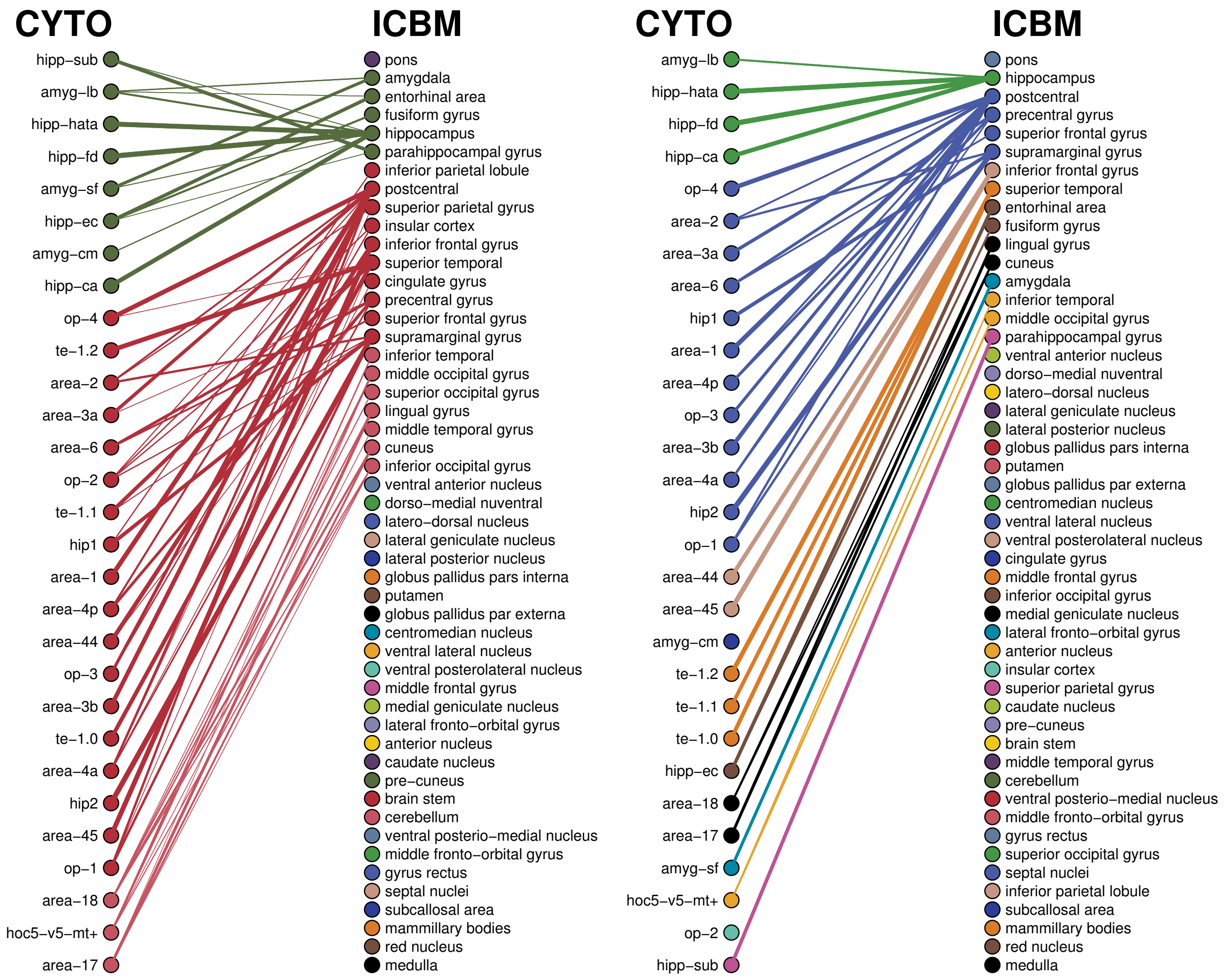

Figure 9: Left: Edges pruned up to $\theta = 0.10$; Right: Edges pruned up to $\theta = 0.25$.

1.10 CYTO - LPBA

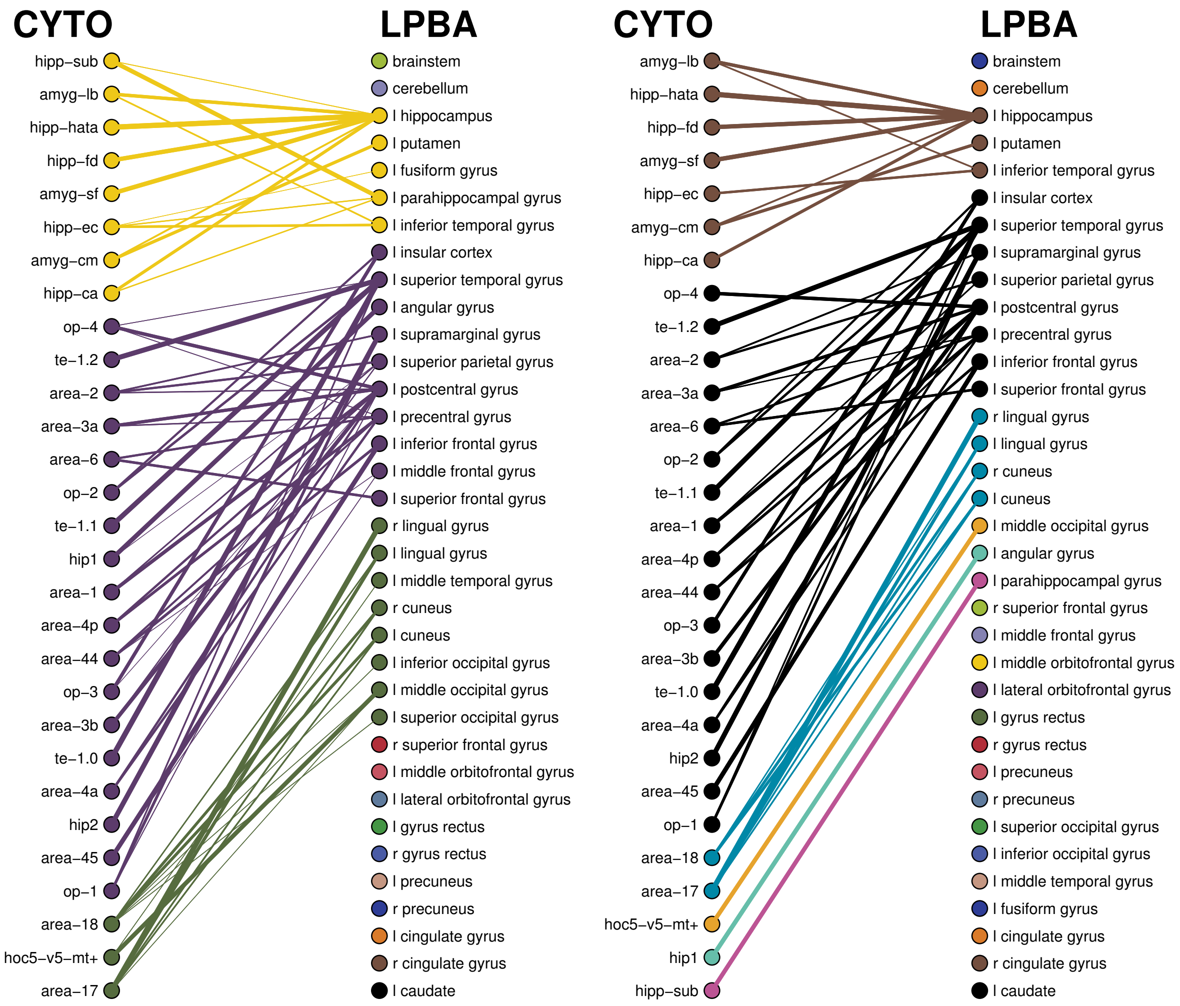

Figure 10: Left: Edges pruned up to $\theta = 0.10$; Right: Edges pruned up to $\theta = 0.25$.

1.11 CYTO - T&G

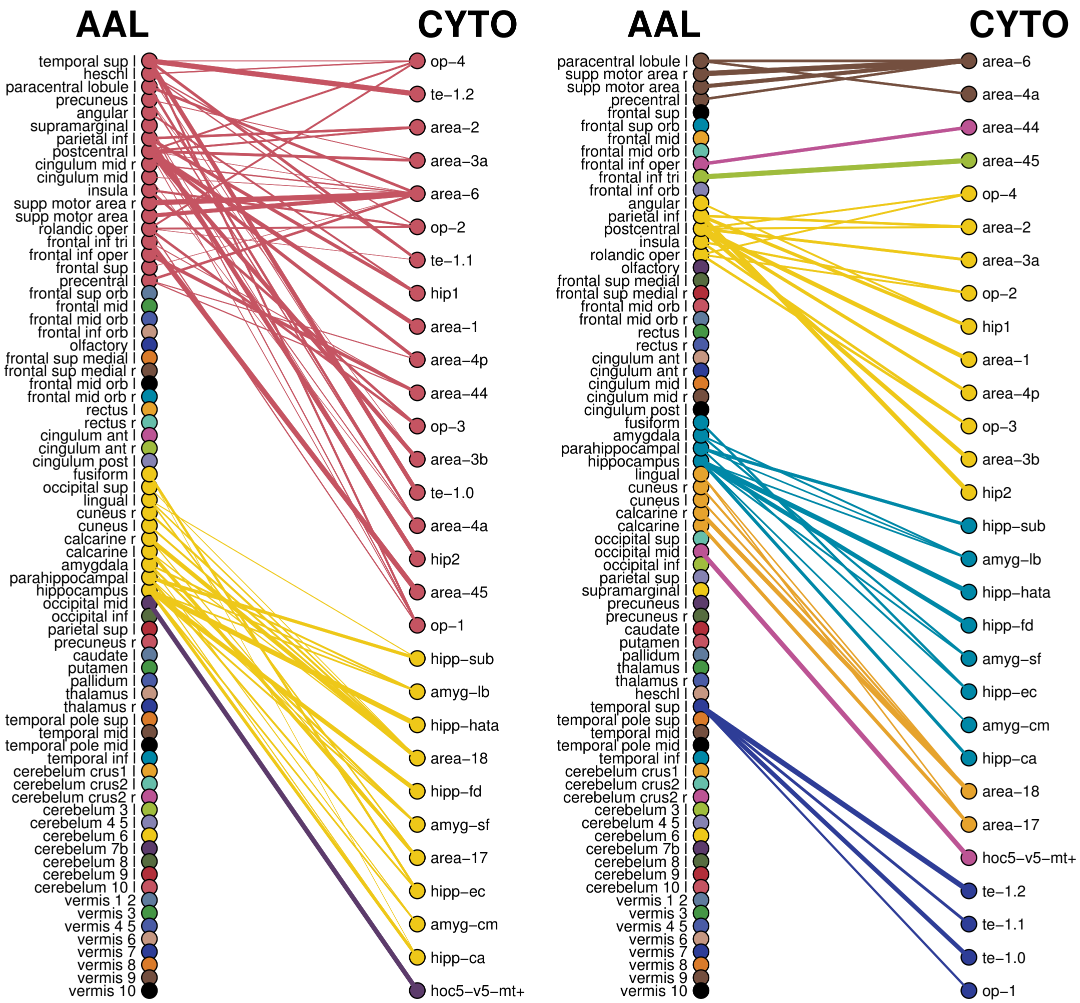

Figure 11: Left: Edges pruned up to $\theta = 0.10$; Right: Edges pruned up to $\theta = 0.25$.

1.12 CYTO - TALc

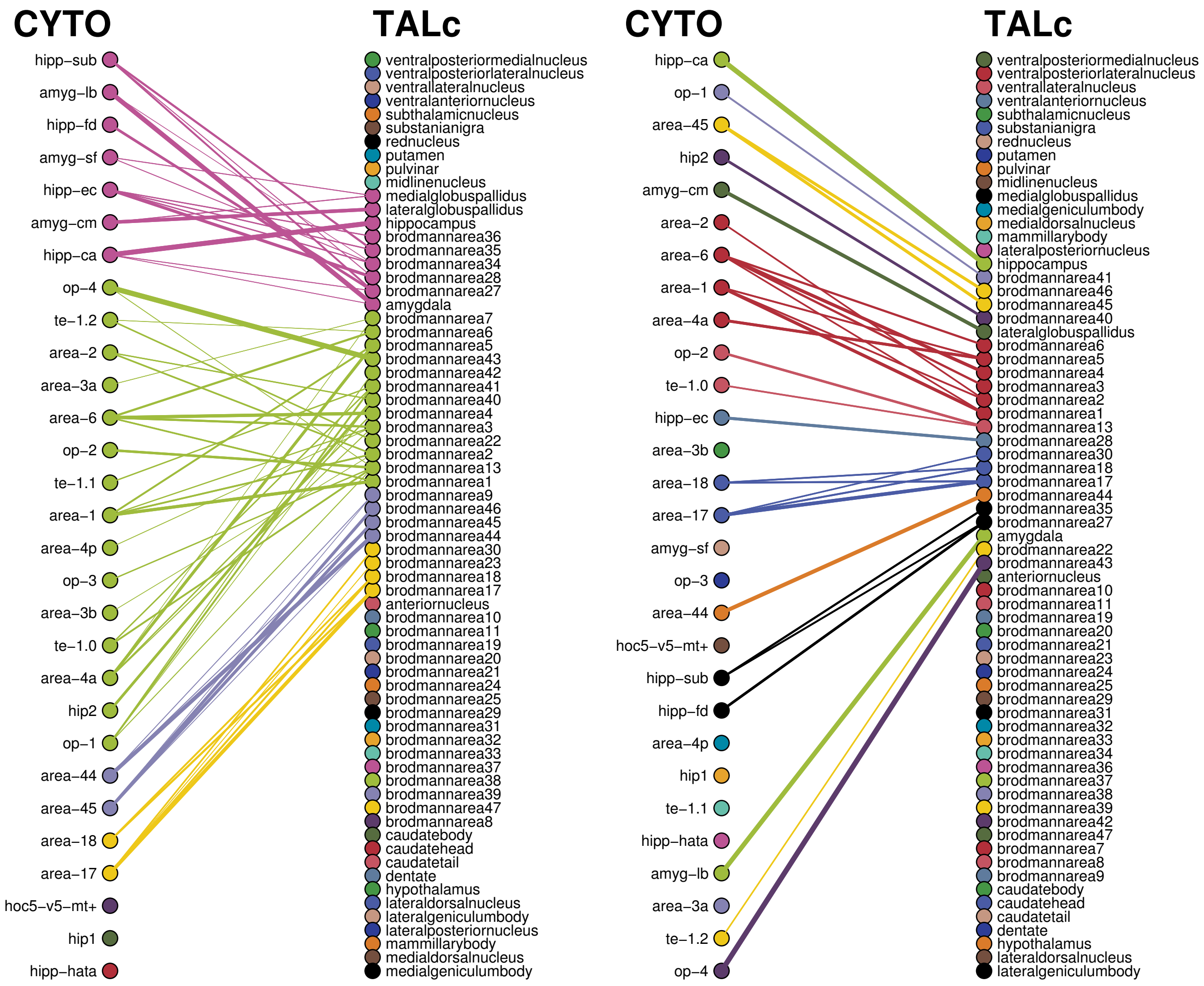

Figure 12: Left: Edges pruned up to $\theta = 0.10$; Right: Edges pruned up to $\theta = 0.25$.

1.13 CYTO - TALg

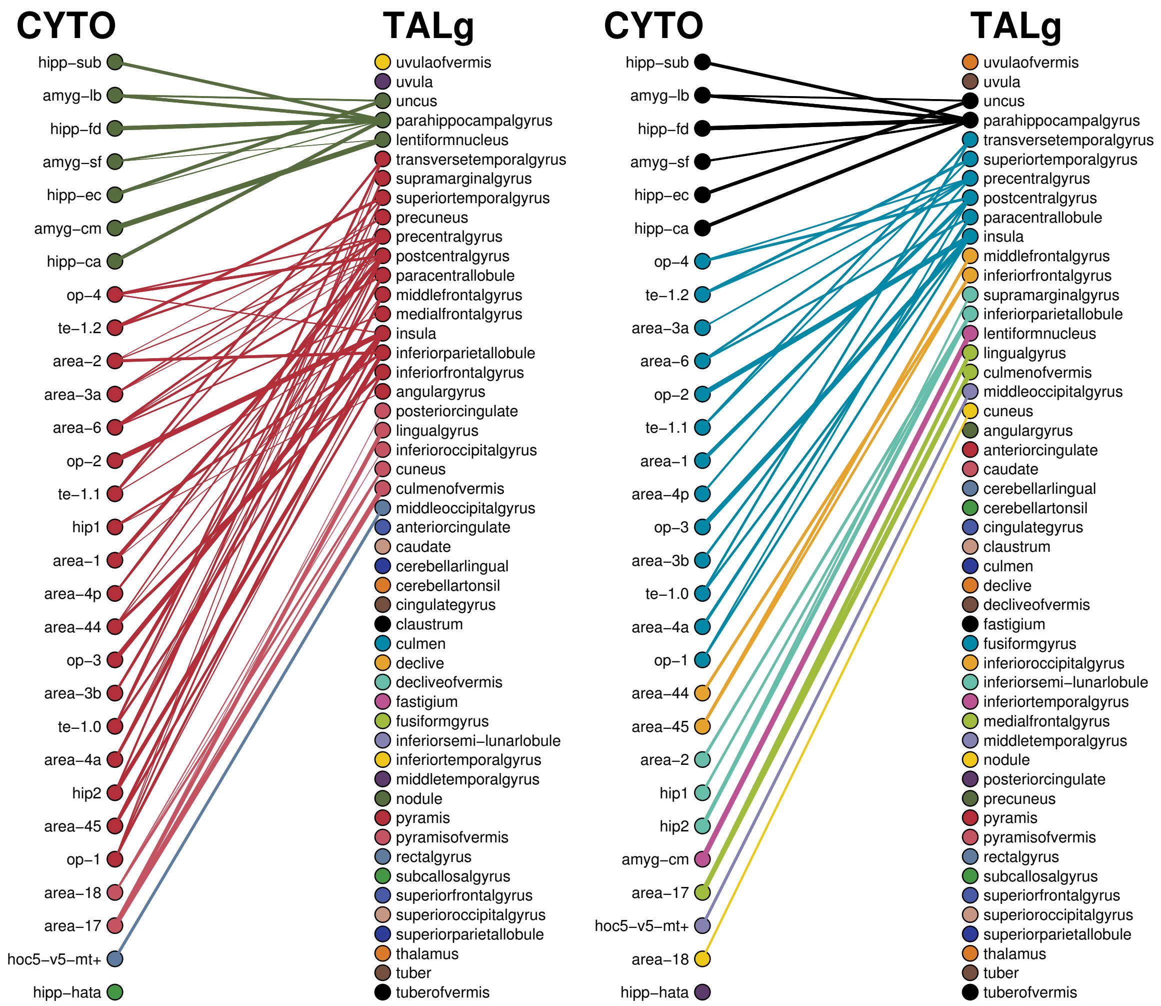

Figure 13: Left: Edges pruned up to $\theta = 0.10$; Right: Edges pruned up to $\theta = 0.25$.

1.14 H-O - ICBM

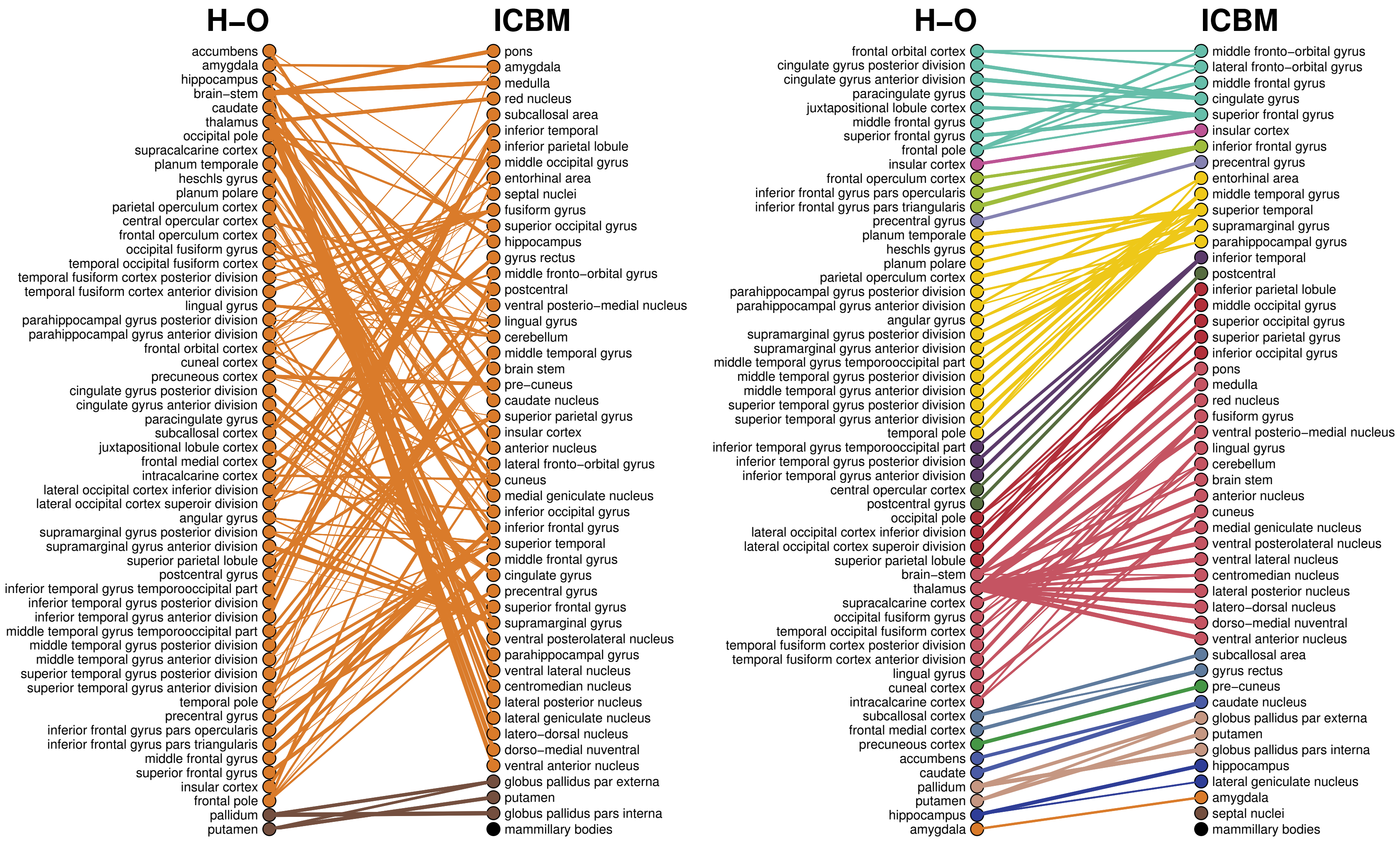

Figure 14: Left: Edges pruned up to $\theta = 0.10$; Right: Edges pruned up to $\theta = 0.25$.

1.15 H-O - LPBA

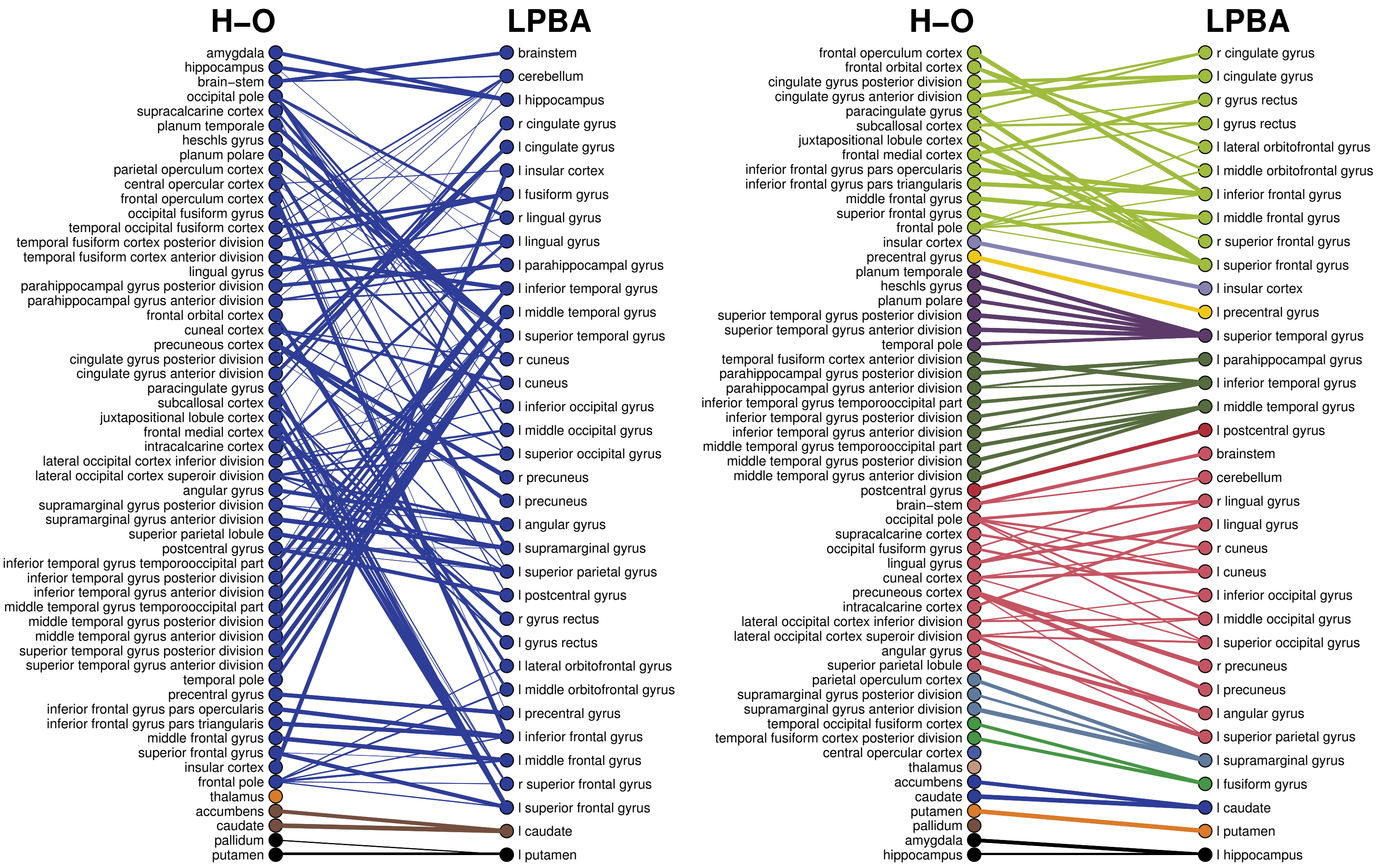

Figure 15: Left: Edges pruned up to $\theta = 0.10$; Right: Edges pruned up to $\theta = 0.25$.

1.16 H-O - T&G

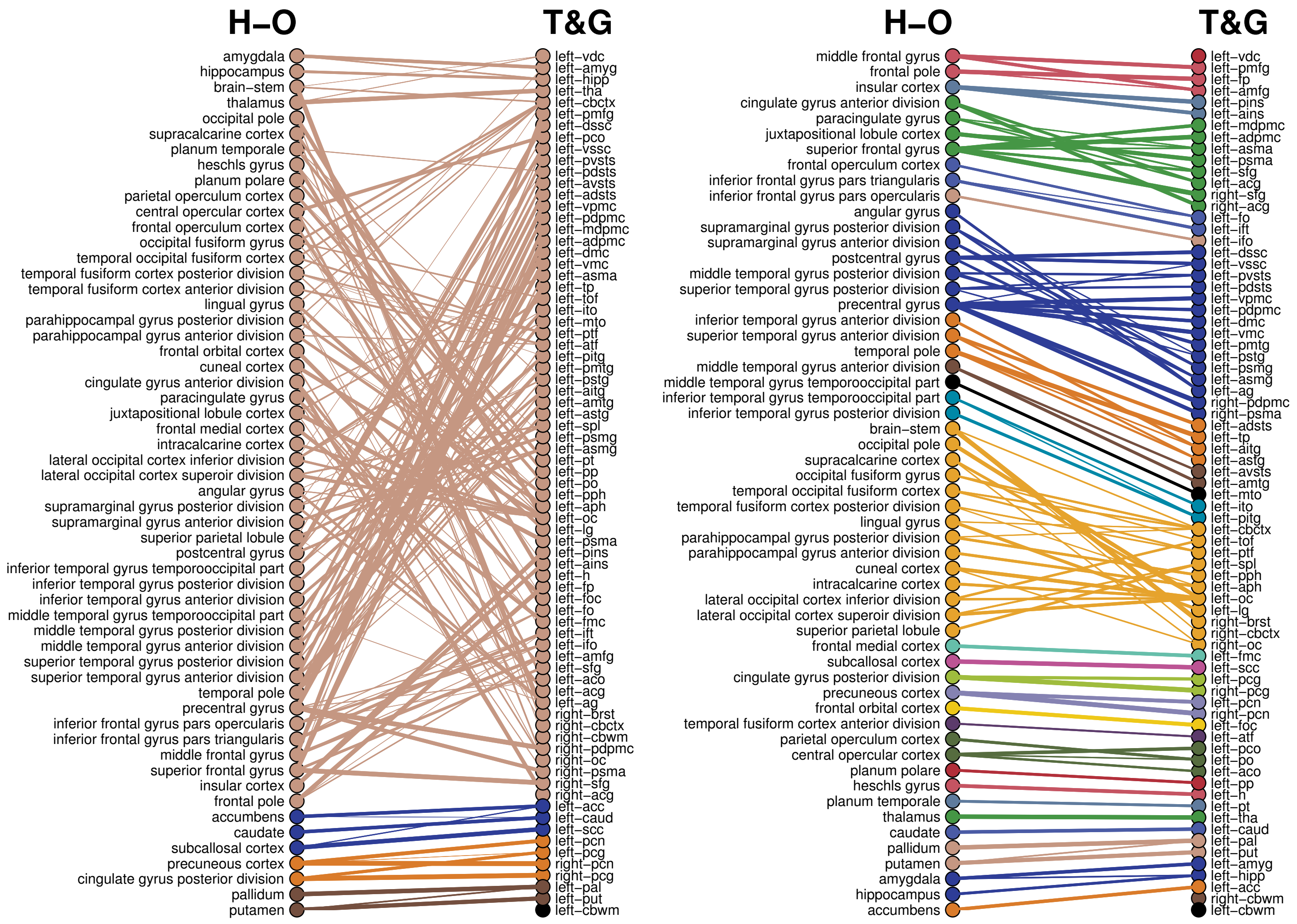

Figure 16: Left: Edges pruned up to $\theta = 0.10$; Right: Edges pruned up to $\theta = 0.25$.

1.17 H-O - TALc

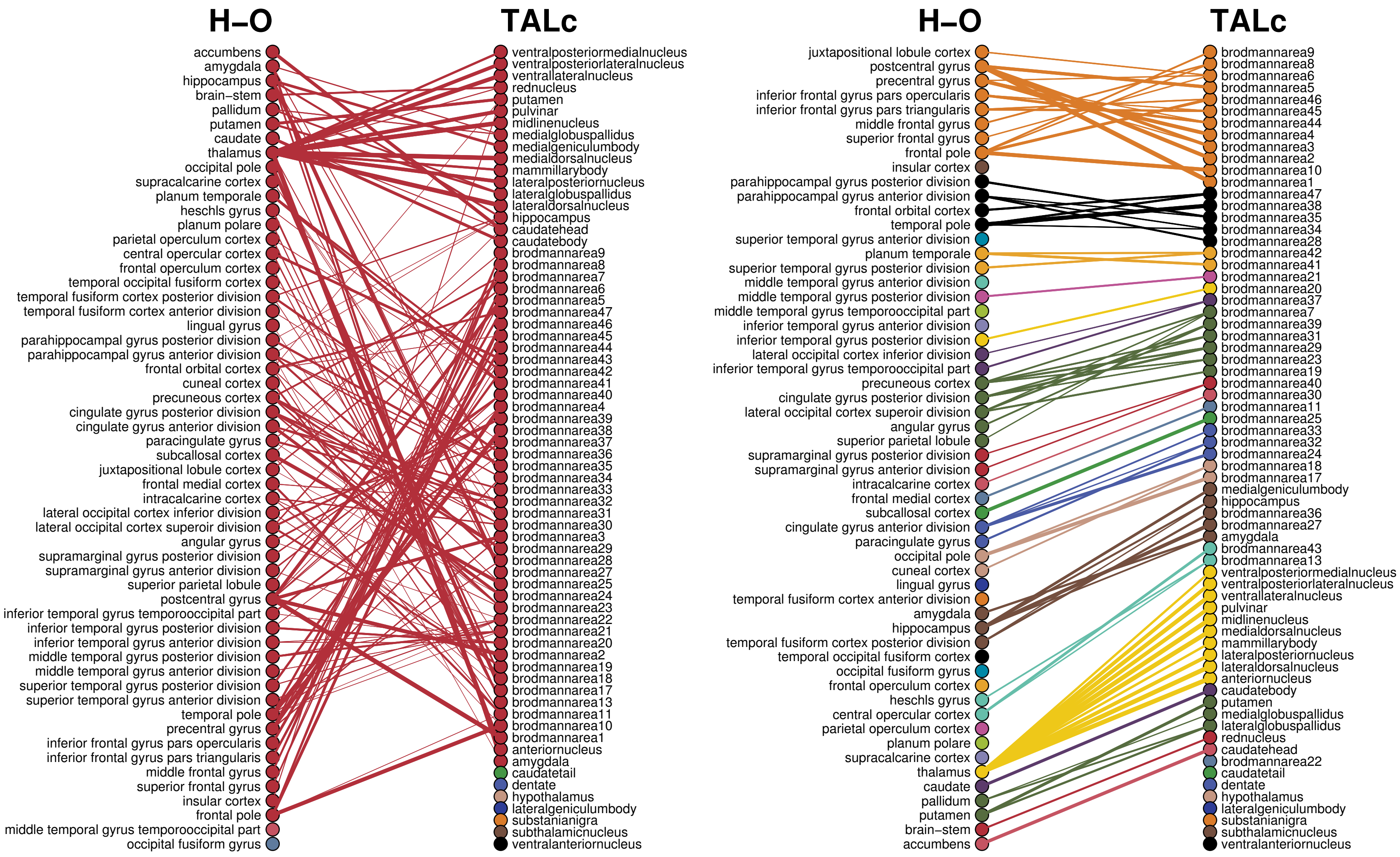

Figure 17: Left: Edges pruned up to $\theta = 0.10$; Right: Edges pruned up to $\theta = 0.25$.

1.18 H-O - TALg

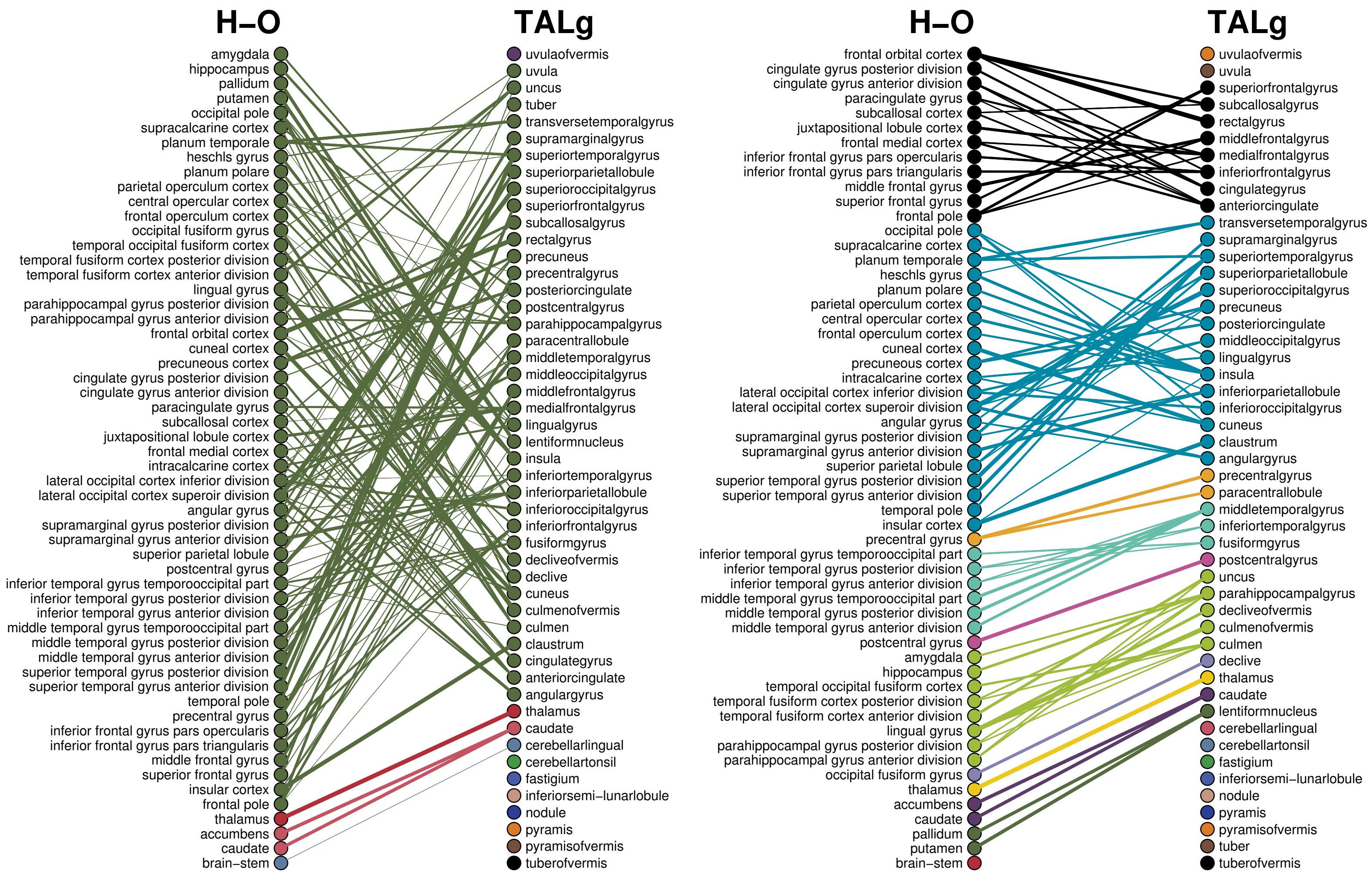

Figure 18: Left: Edges pruned up to $\theta = 0.10$; Right: Edges pruned up to $\theta = 0.25$.

1.19 ICBM - LPBA

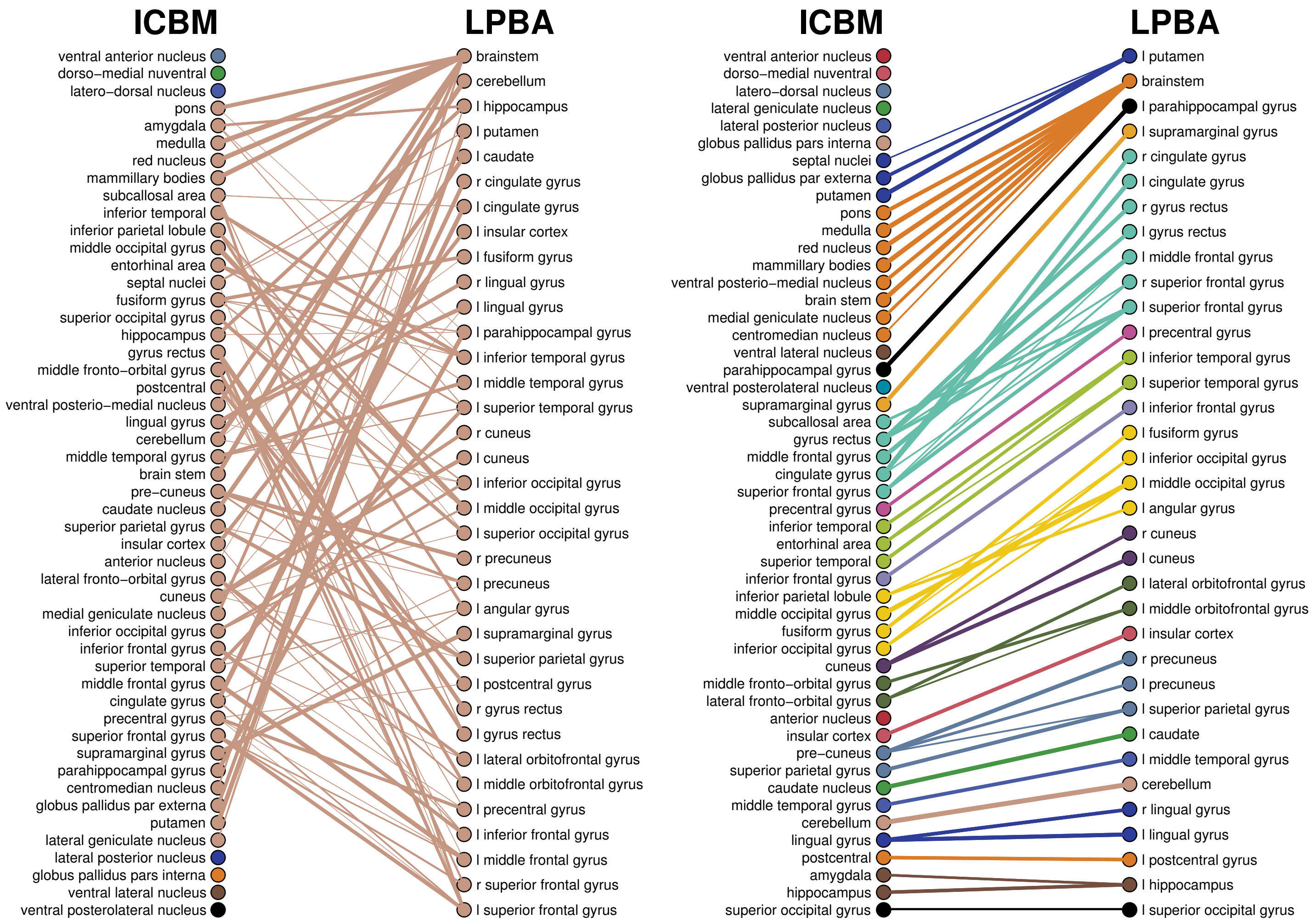

Figure 19: Left: Edges pruned up to $\theta = 0.10$; Right: Edges pruned up to $\theta = 0.25$.

1.20 ICBM - T&G

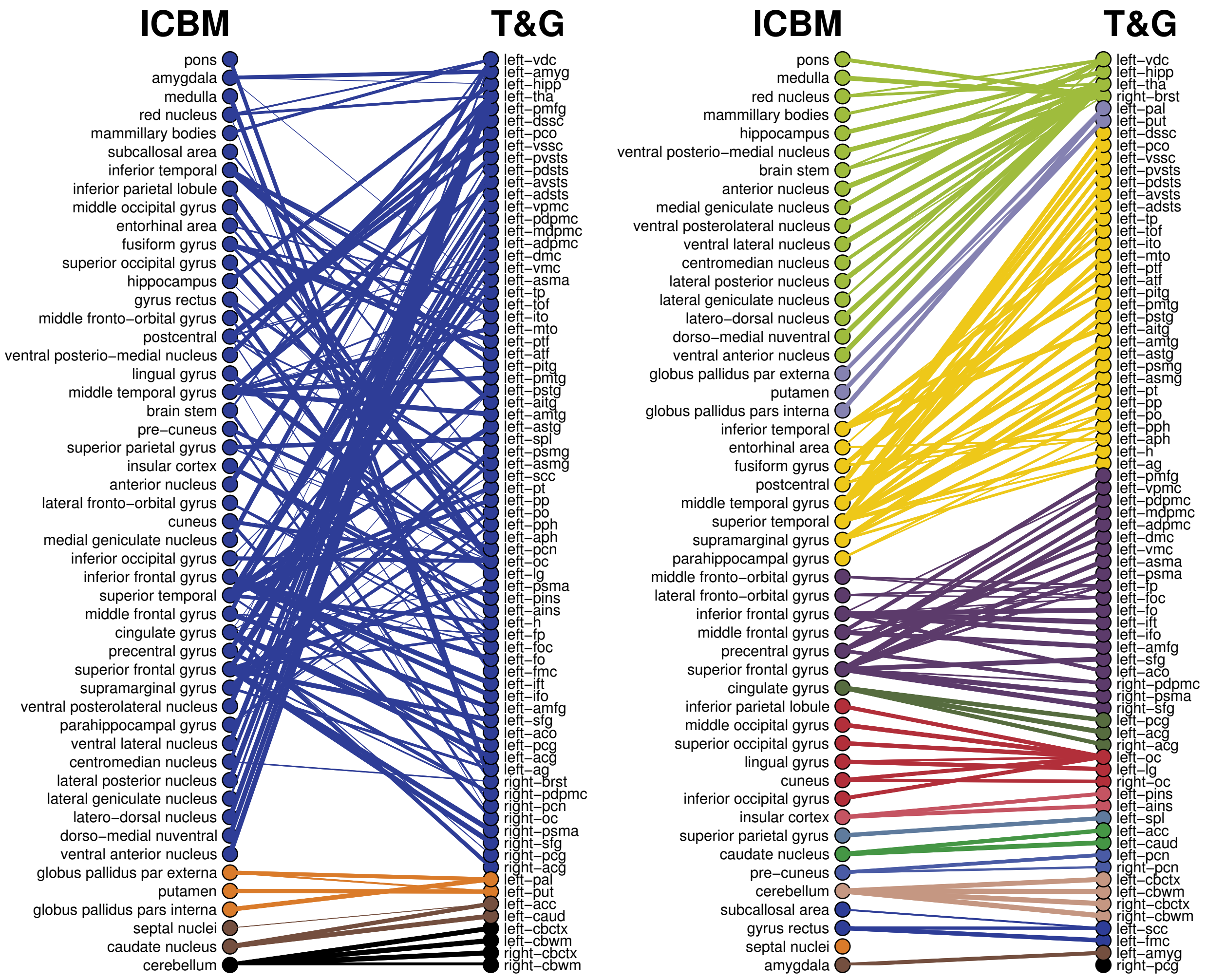

Figure 20: Left: Edges pruned up to $\theta = 0.10$; Right: Edges pruned up to $\theta = 0.25$.

1.21 ICBM - TALc

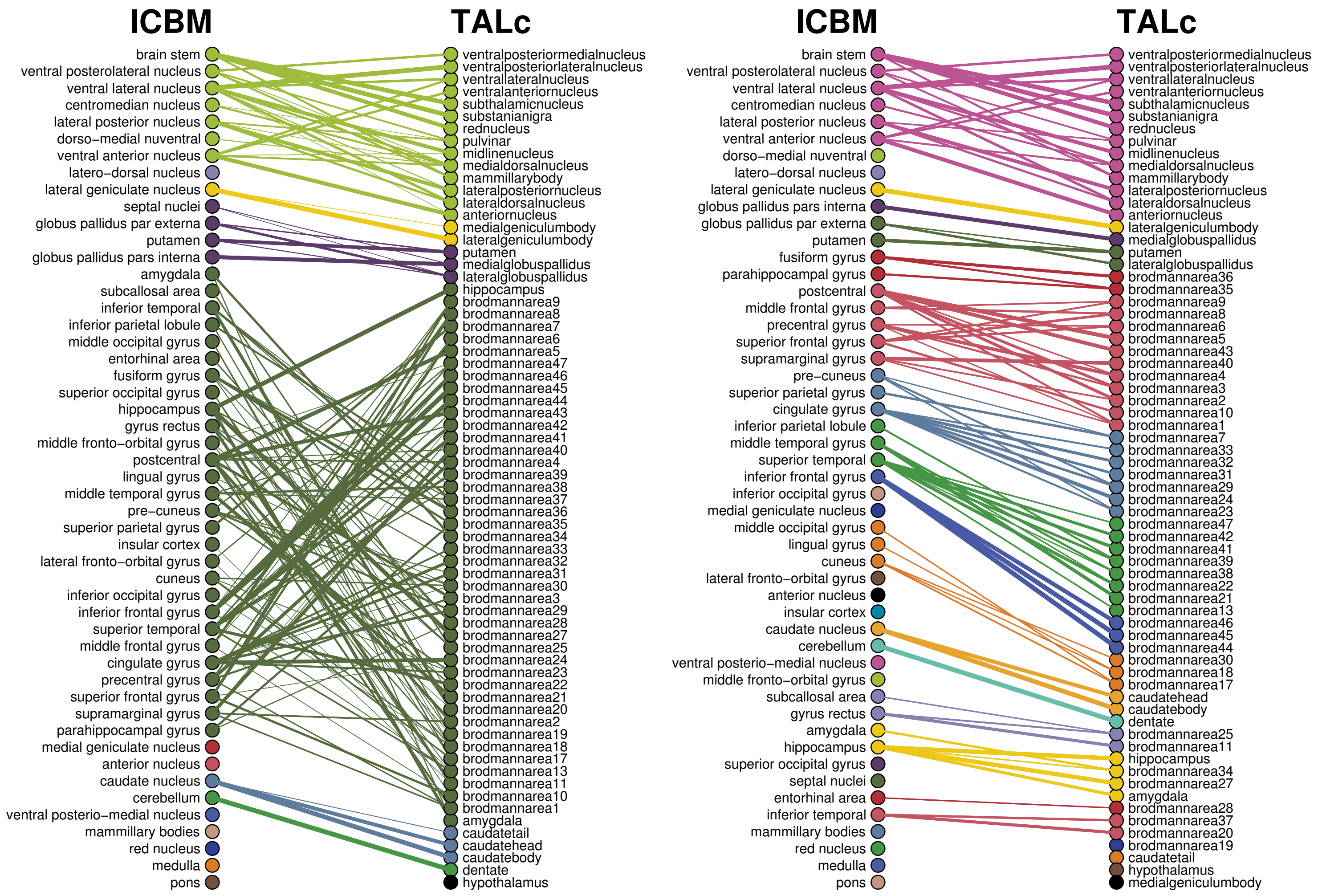

Figure 21: Left: Edges pruned up to $\theta = 0.10$; Right: Edges pruned up to $\theta = 0.25$.

1.22 ICBM - TALg

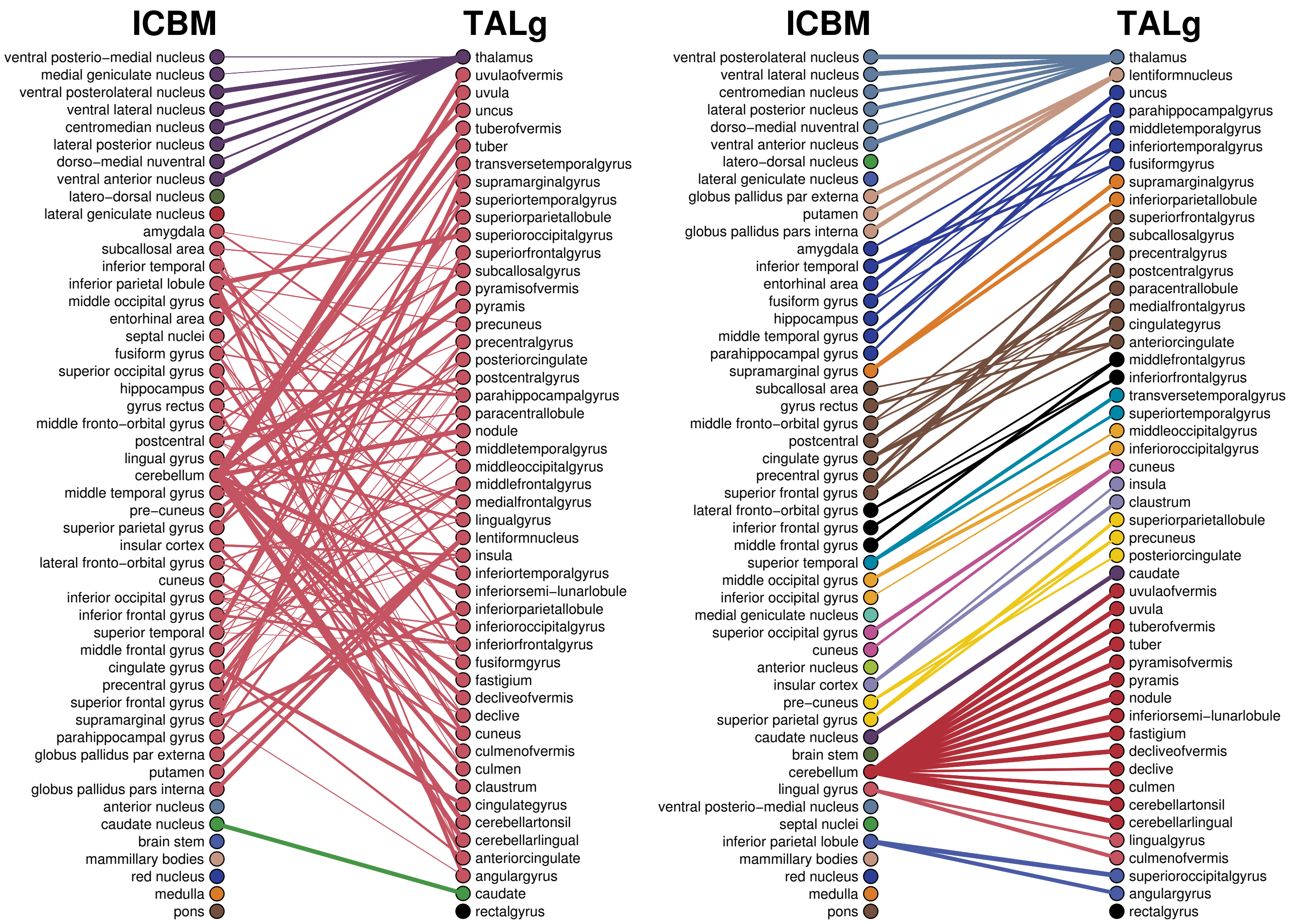

Figure 22: Left: Edges pruned up to $\theta = 0.10$; Right: Edges pruned up to $\theta = 0.25$.

1.23 LPBA - T&G

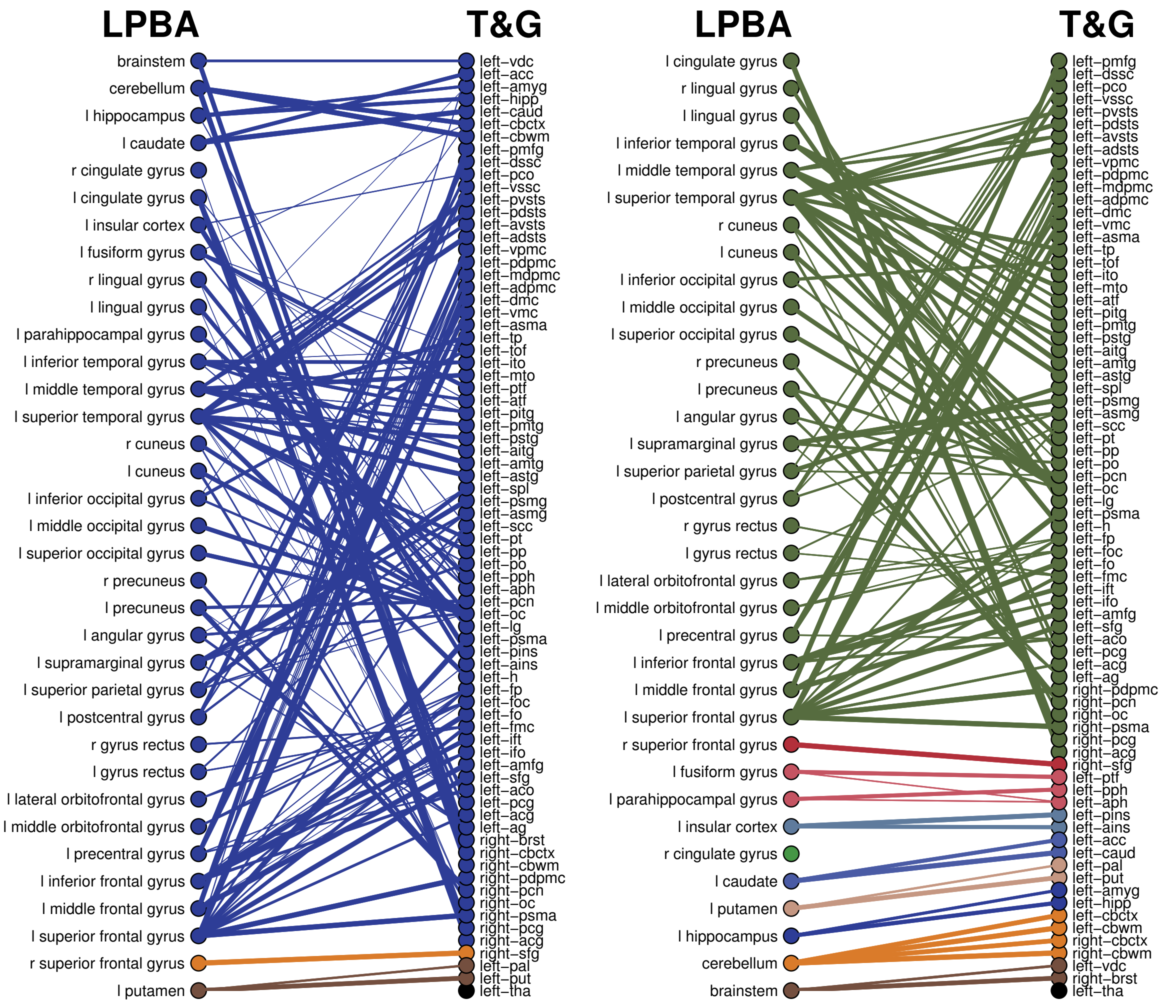

Figure 23: Left: Edges pruned up to $\theta = 0.10$; Right: Edges pruned up to $\theta = 0.25$.

1.24 LPBA - TALc

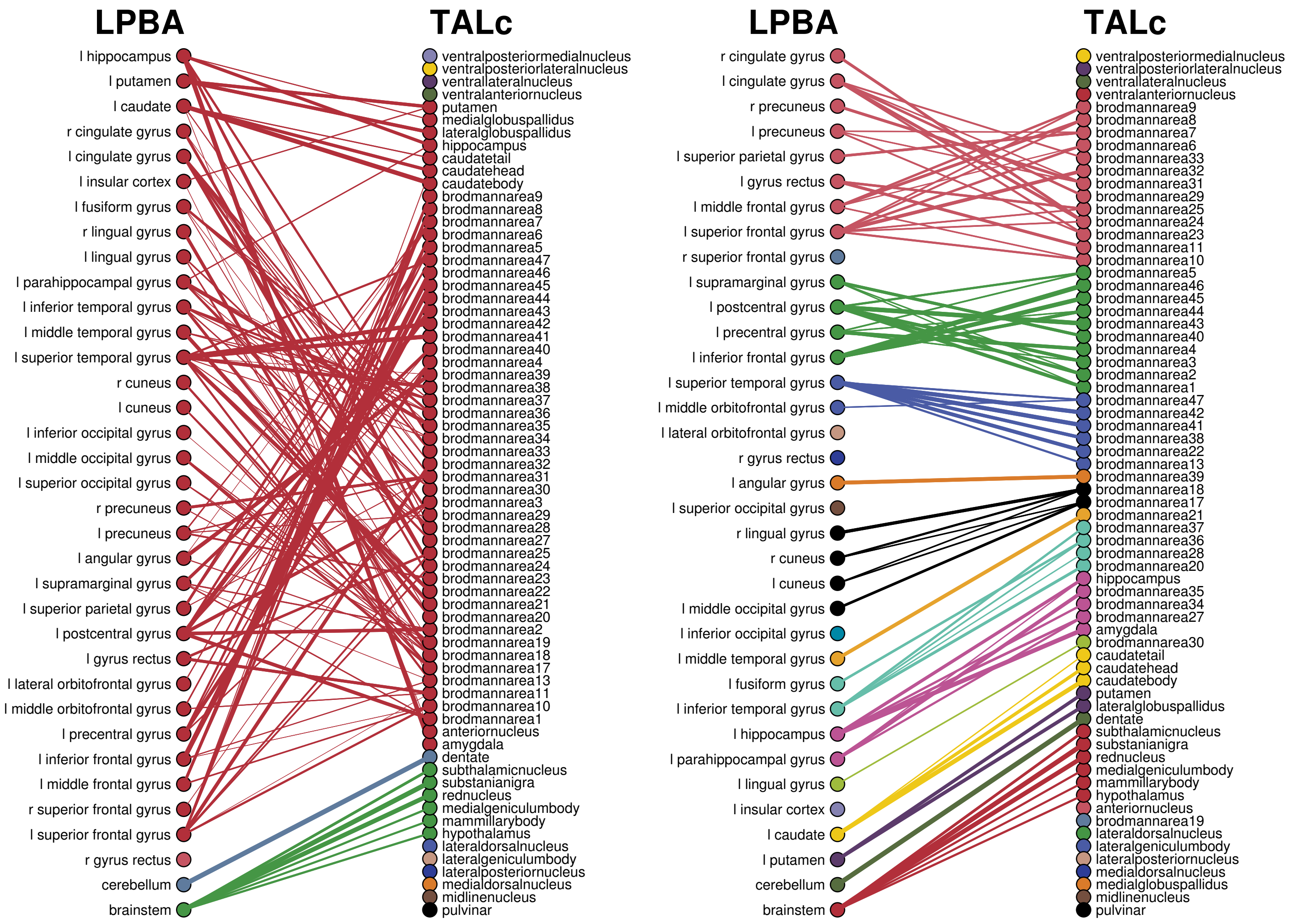

Figure 24: Left: Edges pruned up to $\theta = 0.10$; Right: Edges pruned up to $\theta = 0.25$.

1.25 LPBA - TALg

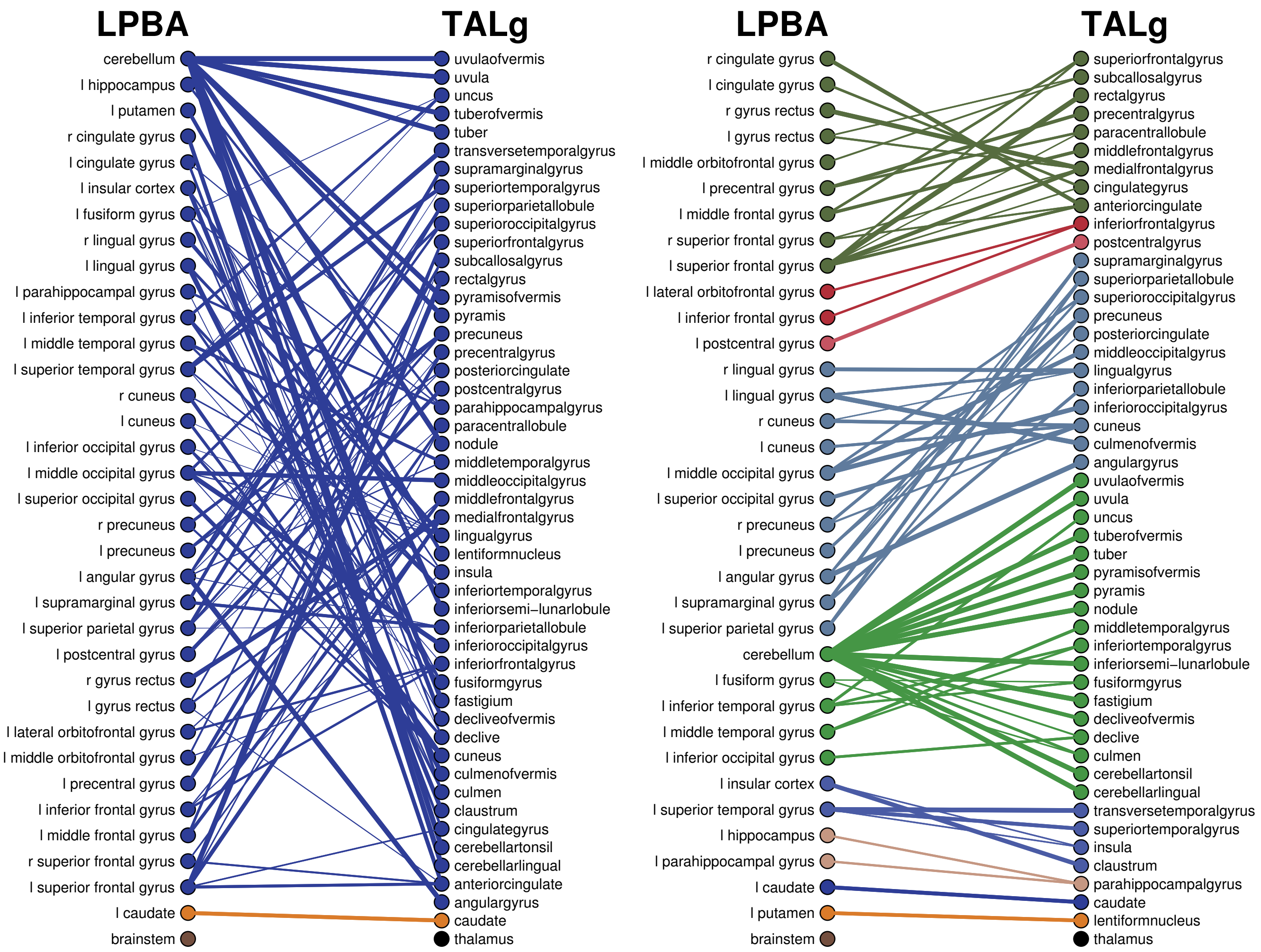

Figure 25: Left: Edges pruned up to $\theta = 0.10$; Right: Edges pruned up to $\theta = 0.25$.

1.26 T&G - TALc

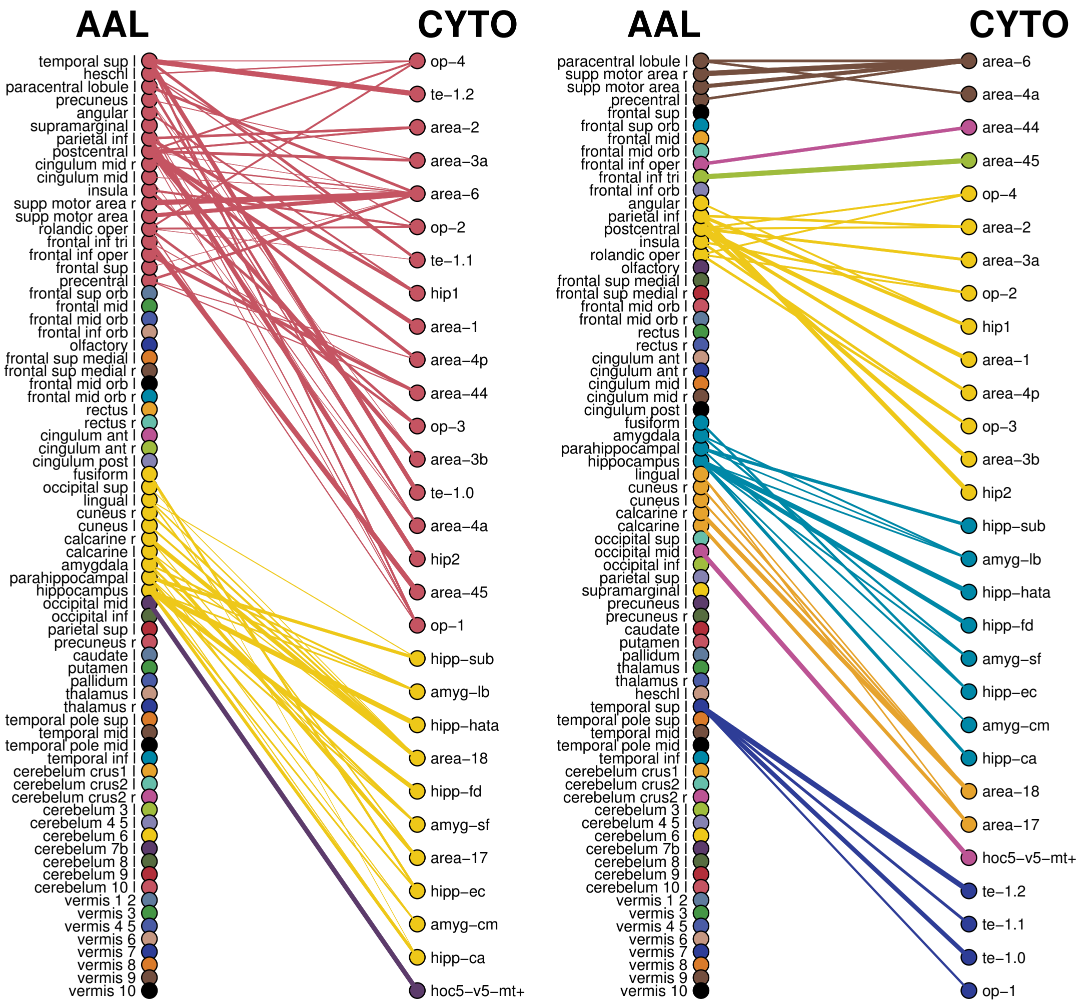

Figure 26: Left: Edges pruned up to $\theta = 0.10$; Right: Edges pruned up to $\theta = 0.25$.

1.27 T&G - TALg

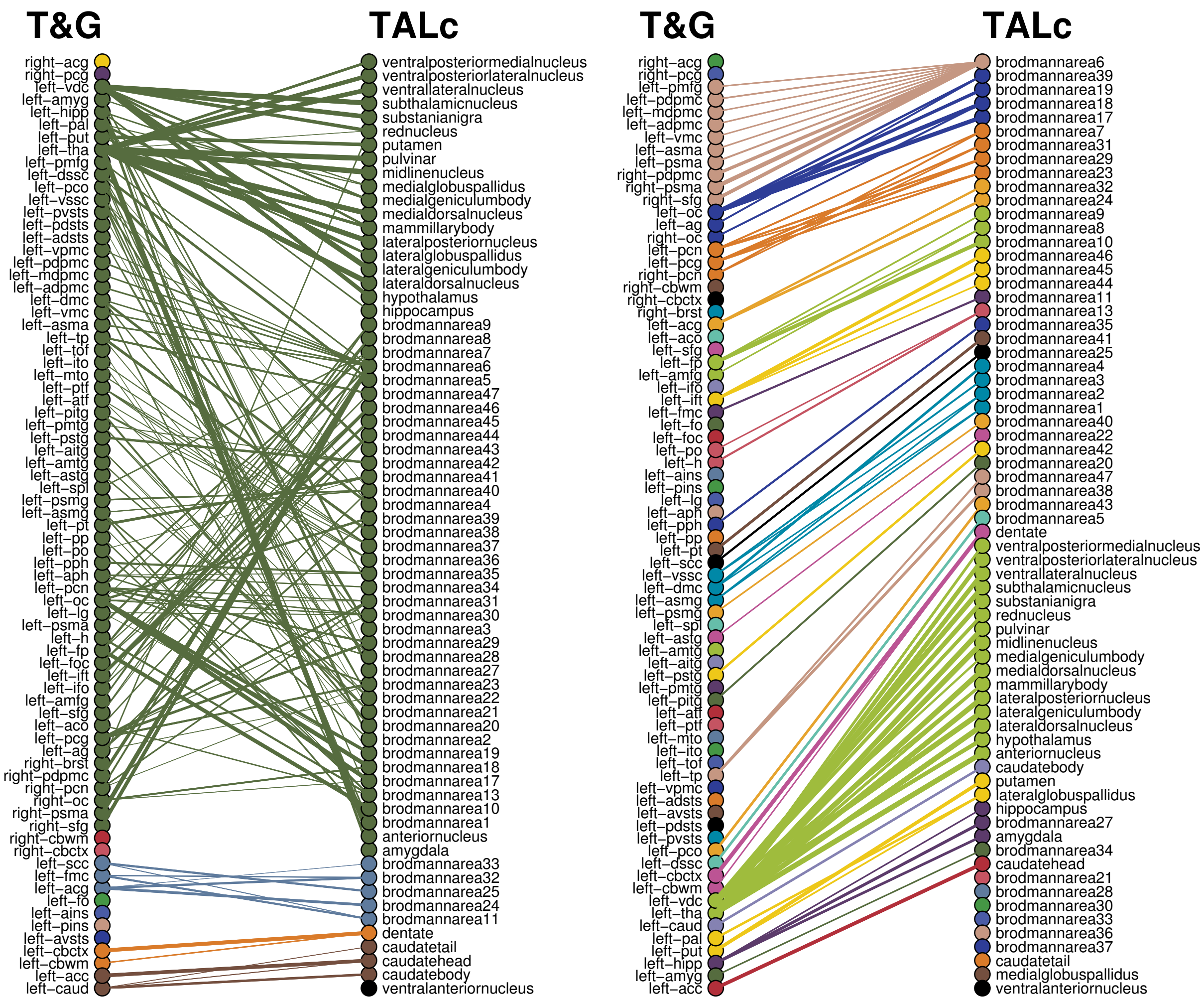

Figure 27: Left: Edges pruned up to $\theta = 0.10$; Right: Edges pruned up to $\theta = 0.25$.

1.28 TALc - TALg

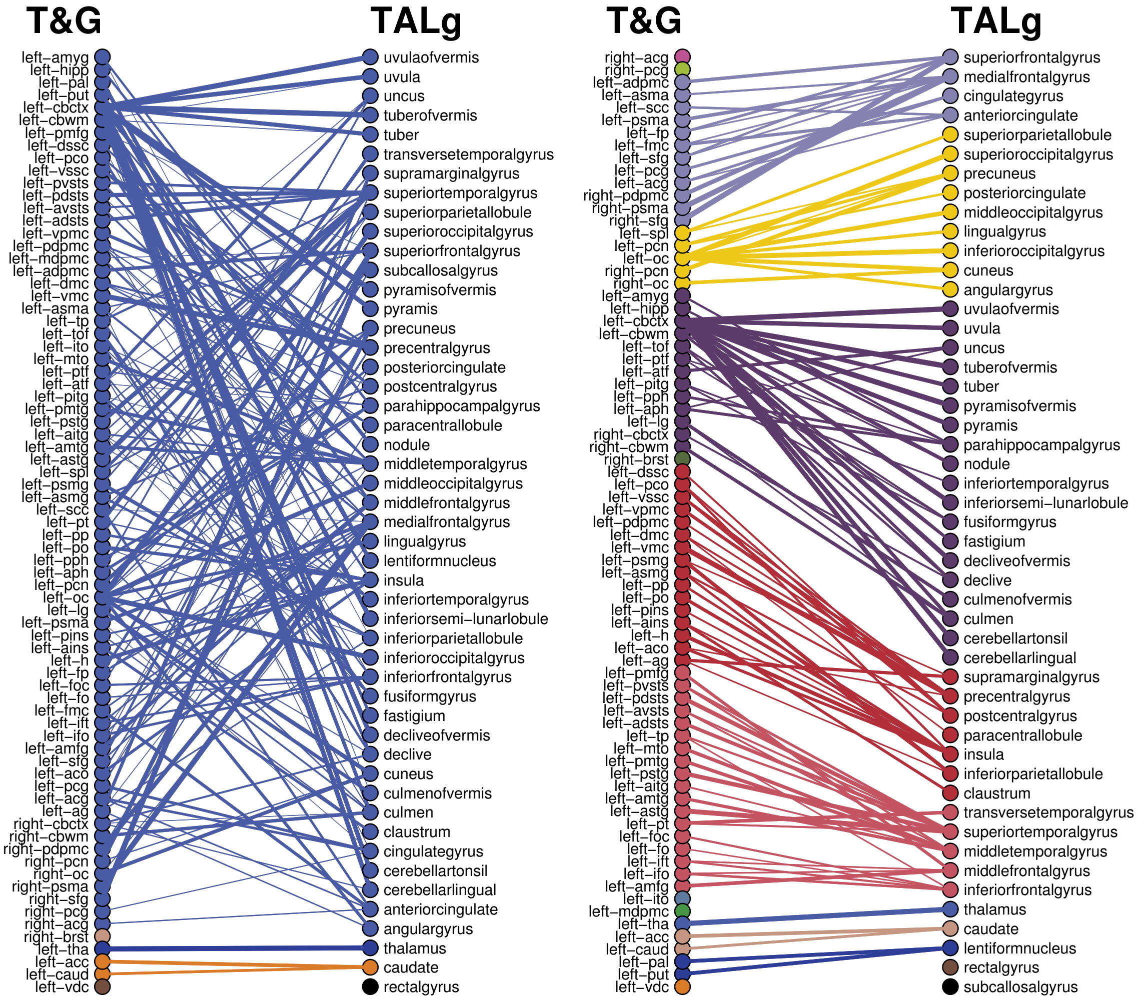

Figure 28: Left: Edges pruned up to $\theta = 0.10$; Right: Edges pruned up to $\theta = 0.25$.
