# Supplementary material for: The Brain Atlas Concordance Problem: Quantitative Comparison of Anatomical Parcellations
Source: PLoS One. 2009 Sep 29;4(9):e7200. doi: 10.1371/journal.pone.0007200 (PMC2748707; doi:10.1371/journal.pone.0007200)
Supplement: Text S2 — Supplemental analysis based on TALcNH parcellation. (0.29 MB PDF) [file pone.0007200.s003.pdf]

The brain atlas concordance problem: quantitative comparison of anatomical parcellations
Supporting Text S2

Jason W. Bohland, Hemant Bokil, Cara B. Allen, and Partha P. Mitra

Contents

1 Supplemental analysis based on the TALc^{NH} parcellation **2**

2 Bipartite graph comparisons for TALc^{NH} **2**

2.1 AAL - TALc^{NH} 3
2.2 CYTO - TALcNH 4
2.3 H-O - TALcNH 5
2.4 ICBM - TALc^{NH} 6
2.5 LPBA40 - TALc^{NH} 7
2.6 T G - TALc^{NH} 8
2.7 TALc^{NH} - TALc 9
2.8 TALc^{NH} - TALg 10

1 Supplemental analysis based on the TALc^{NH} parcellation

The *TALc*^{NH} parcellation was created by modifyinig the *TALc* parcellation described in the main text. Specifically, for each unlabled voxel in *TALc* belonging to the set of voxels determined to be grey matter in the test brain, we found the nearest non-trivially (non-zero) labeled voxel. If that voxel was within a 5 mm radius, then its label was assigned to the target voxel. This resulted in 78.8% of voxels in the calculated GM of the test brain being assigned a label (compared to 26.5% in *TALc*).

Figure 1 shows sections through the two versions of the *TALc* parcellation, overlaid on the single subject test brain.

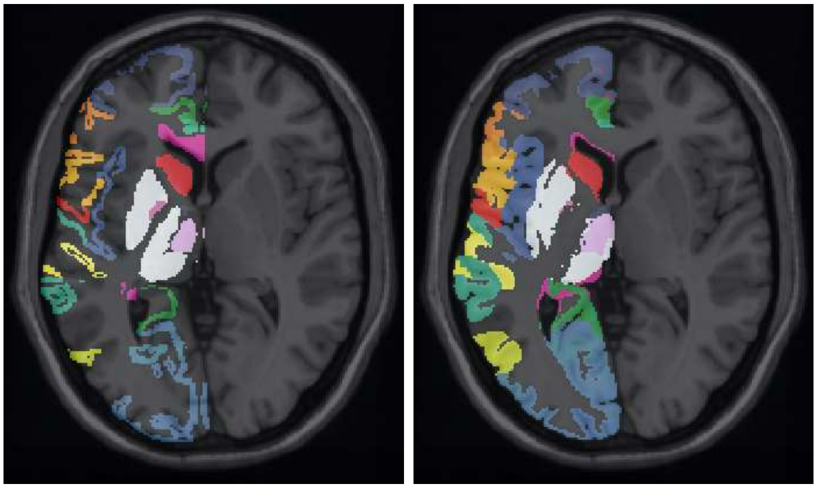

Figure 1: Sample sections depicting the differences between the *TALc* and *TALc*^{NH} parcellations, overlaid on the test brain. It is clear that the "neighborhood" labeling at least partially addresses misregistration issues inherent in the *TALc* labeling method, and the overall concordance of this parcellation to the other parcellations is increased significantly.

	AAL	H-O	ICBM	LPBA40	T&G	TALc	TALg
ARI	0.1563	0.3164	0.3724	0.3665	0.2535	0.0777	0.1732
S-Index	0.6509	0.6422	0.6575	0.6313	0.5921	0.4582	0.5873

Table 1: Global concordance indices for *TALc*^{NH} vs. the other parcellations. ARI = Adjusted Rand Index. See main article text for descriptions.

2 Bipartite graph comparisons for TALc^{NH}

Each pair of parcellations is compared using the bipartite graph formulation described in our paper. The graphs are shown for $\theta = 0.10$ and for $\theta = 0.25$. Note that, for comparison of *TALc* with *TALc*^{NH}, all areas are connected singly to their counterpart. This is because the regions in the neighborhood parcellation are, by definition, supersets of the same areas in *TALc*.

These graphs should be used to augment the understanding of how Talairach Atlas labels found in the literature relate to other anatomical labels, because a similar "neighborhood" method is often employed when using the Talairach Daemon.

2.1 AAL - TALc^{NH}

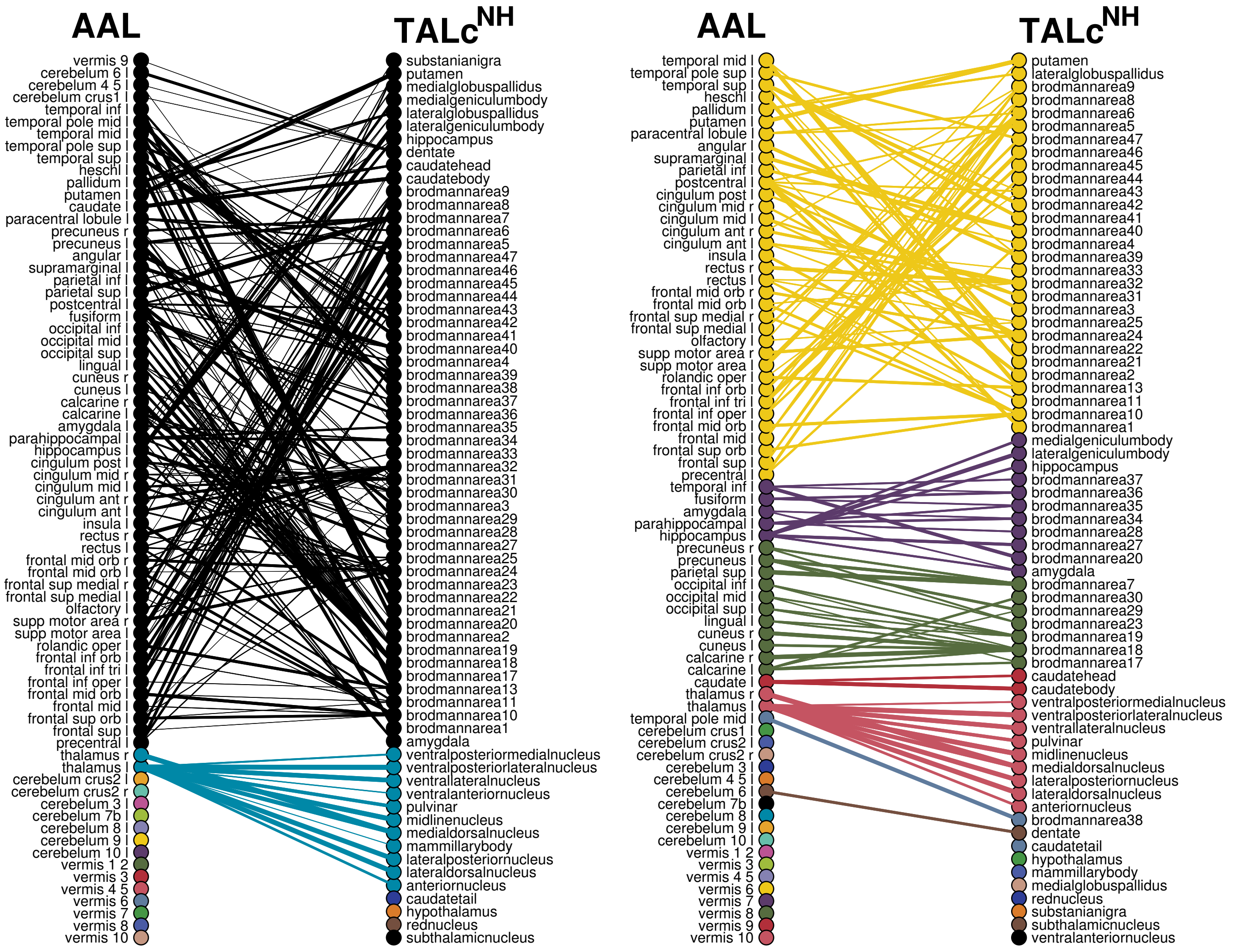

Figure 2: Left: Edges pruned up to $\theta = 0.10$; Right: Edges pruned up to $\theta = 0.25$.

2.2 CYTO - TALcNH

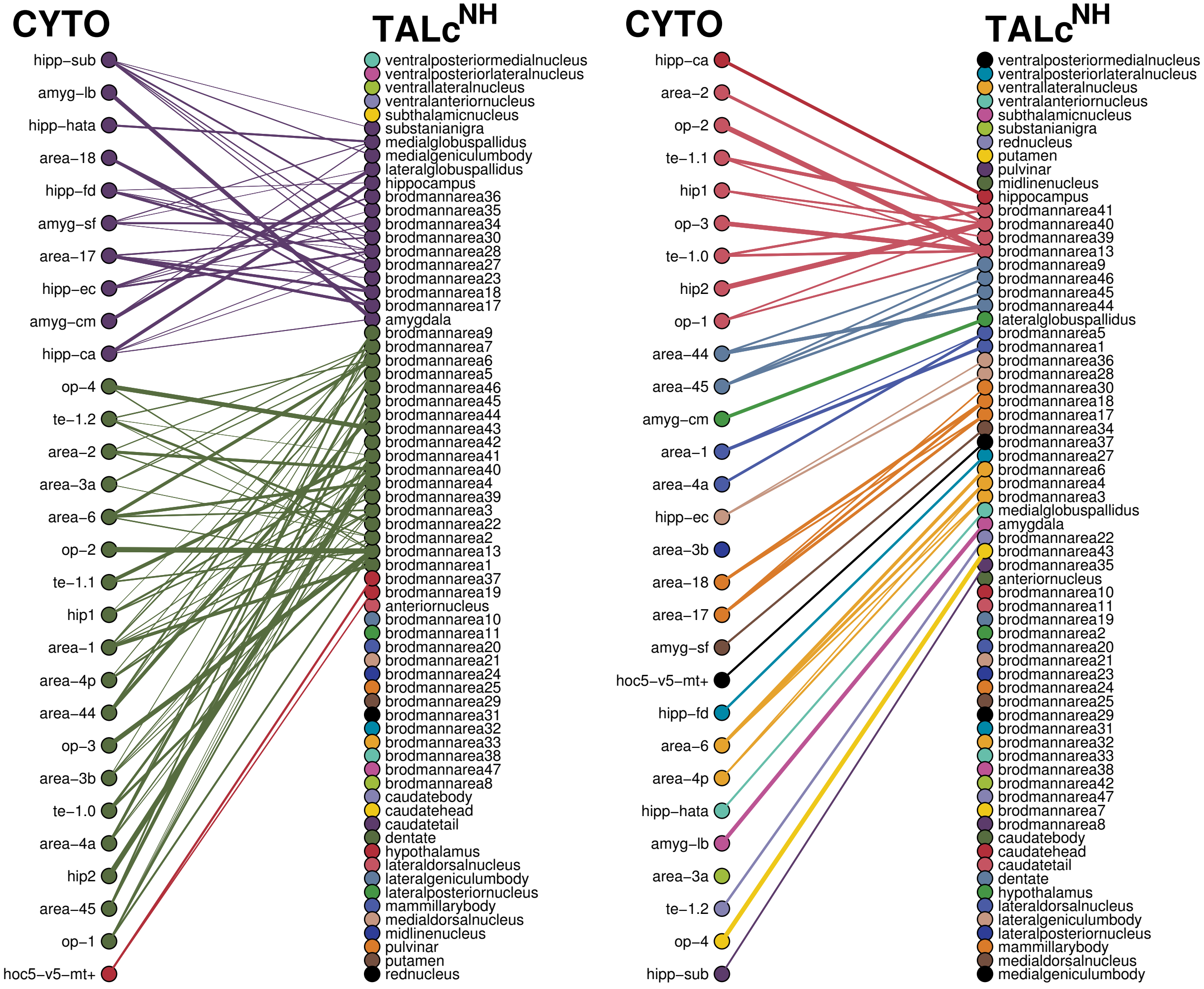

Figure 3: Left: Edges pruned up to $\theta = 0.10$; Right: Edges pruned up to $\theta = 0.25$.

2.3 H-O - TALcNH

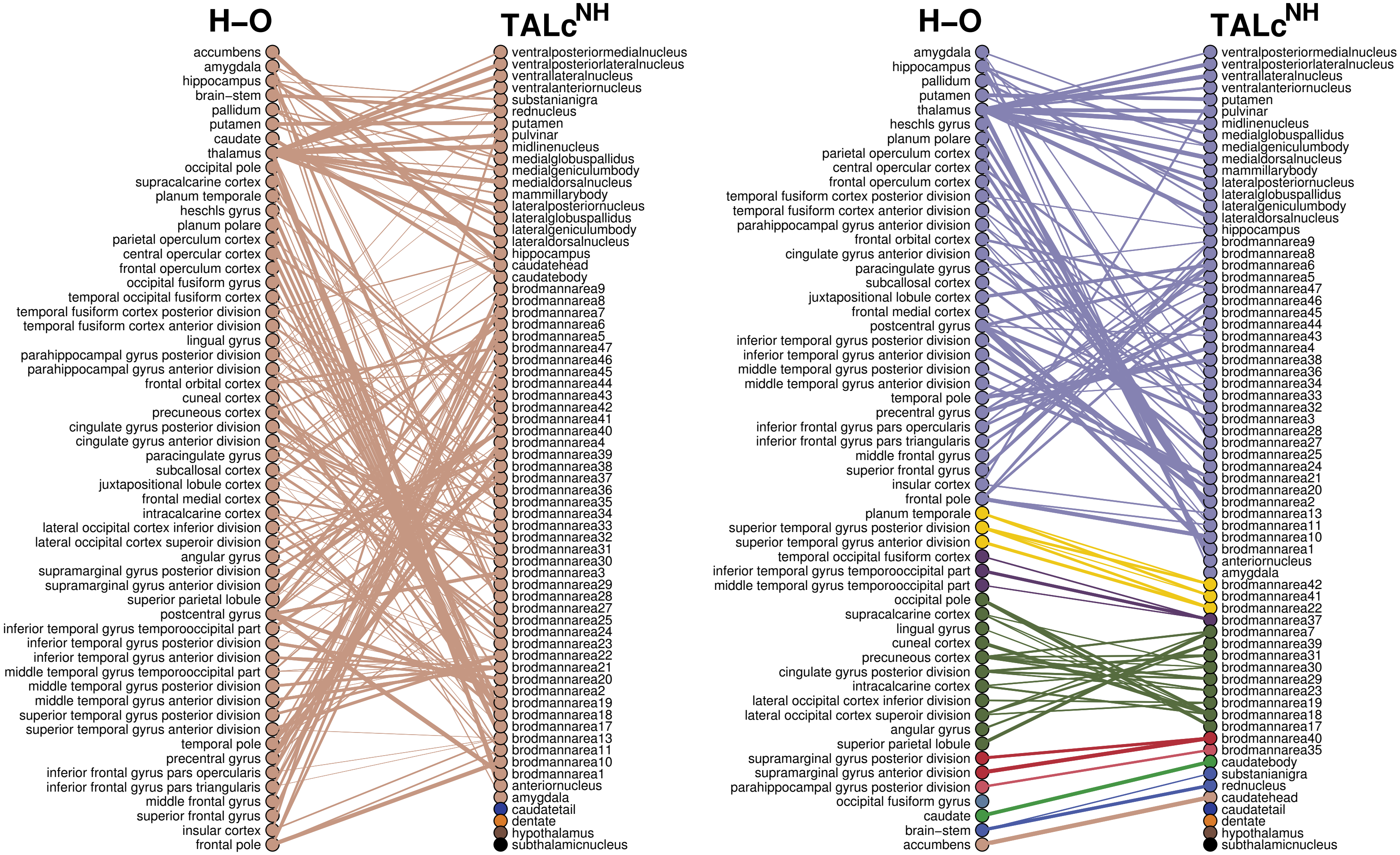

Figure 4: Left: Edges pruned up to $\theta = 0.10$; Right: Edges pruned up to $\theta = 0.25$.

2.4 ICBM - TALc^{NH}

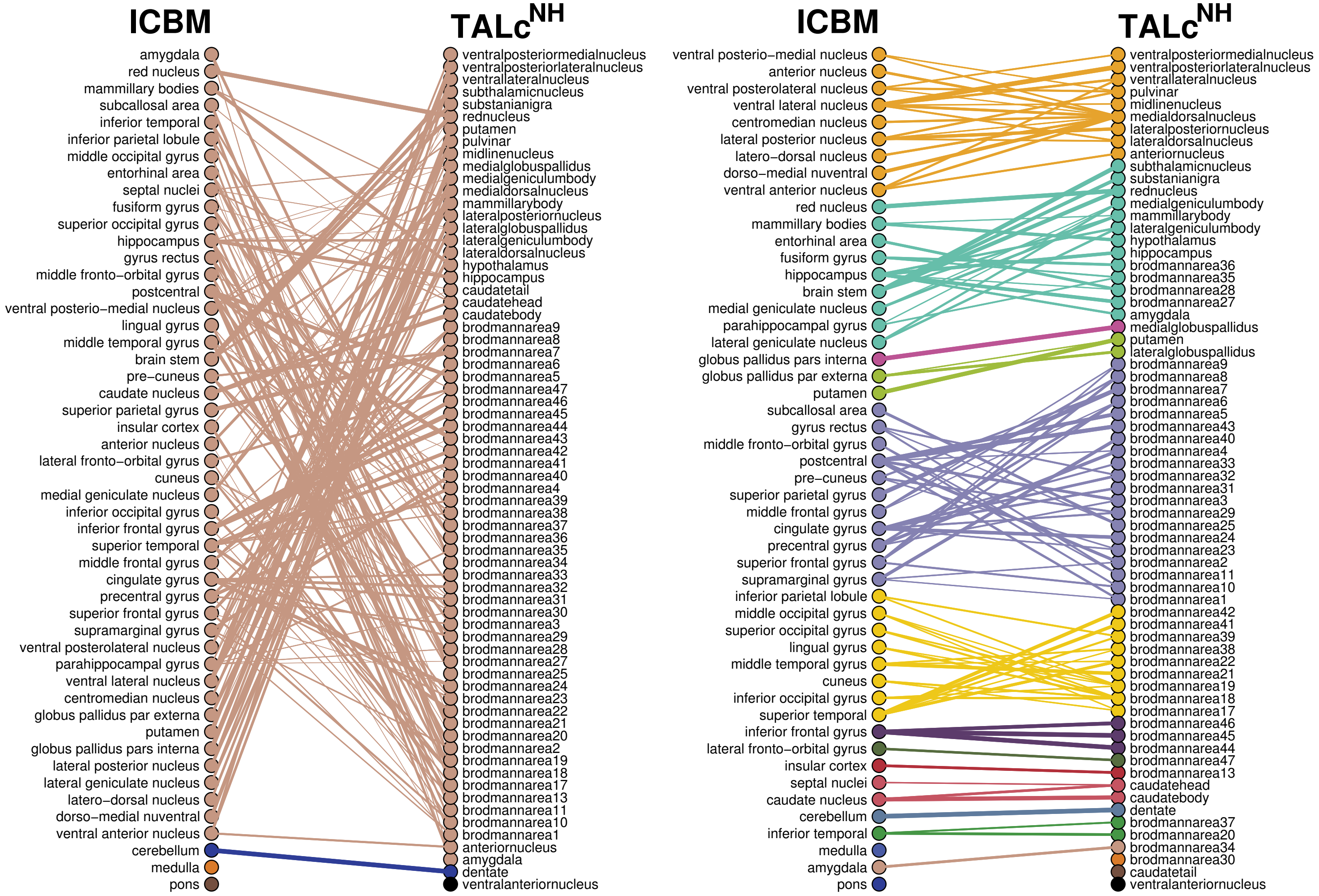

Figure 5: Left: Edges pruned up to $\theta = 0.10$; Right: Edges pruned up to $\theta = 0.25$.

2.5 LPBA40 - TALc^{NH}

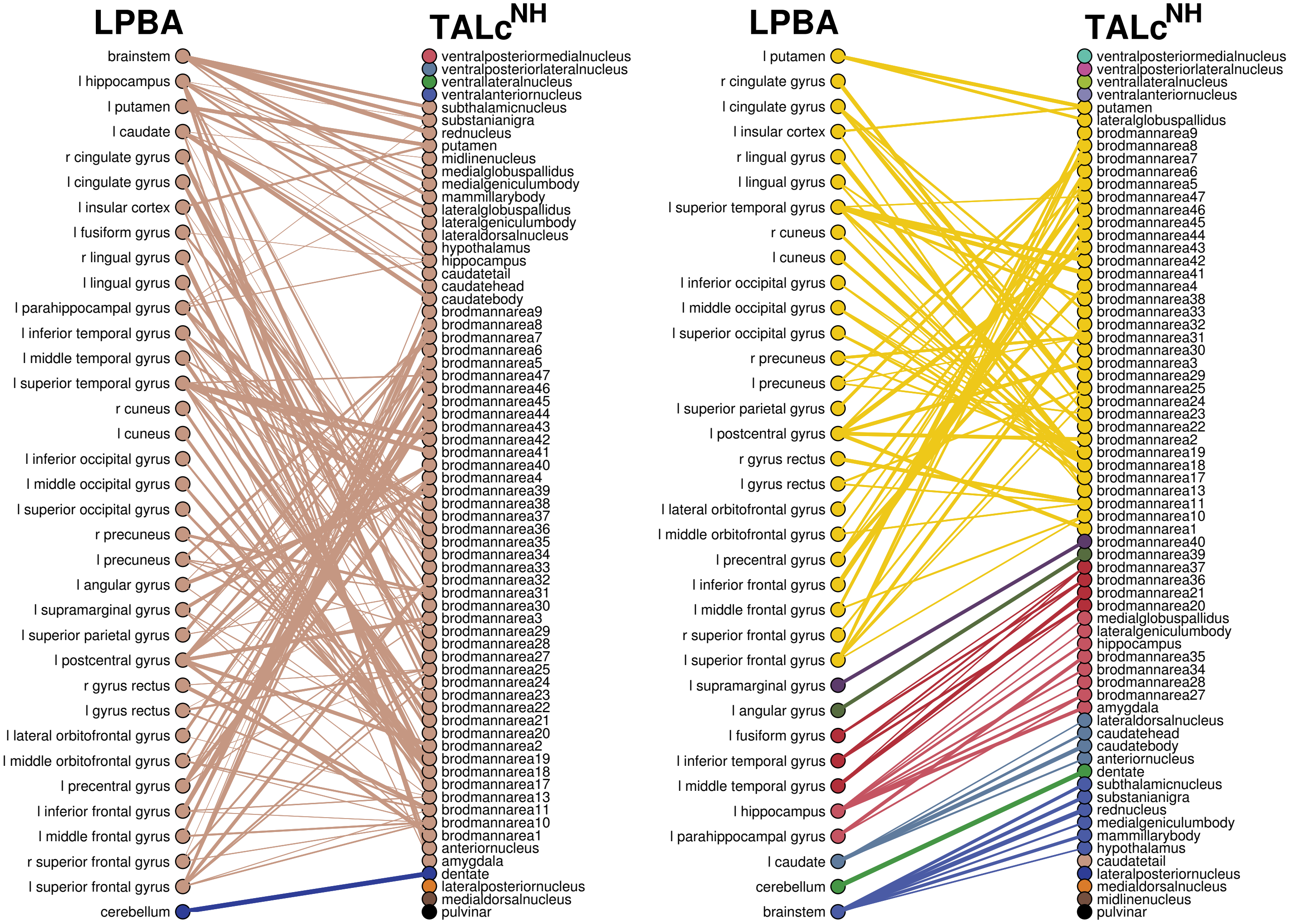

Figure 6: Left: Edges pruned up to $\theta = 0.10$; Right: Edges pruned up to $\theta = 0.25$.

2.6 T G - TALc^{NH}

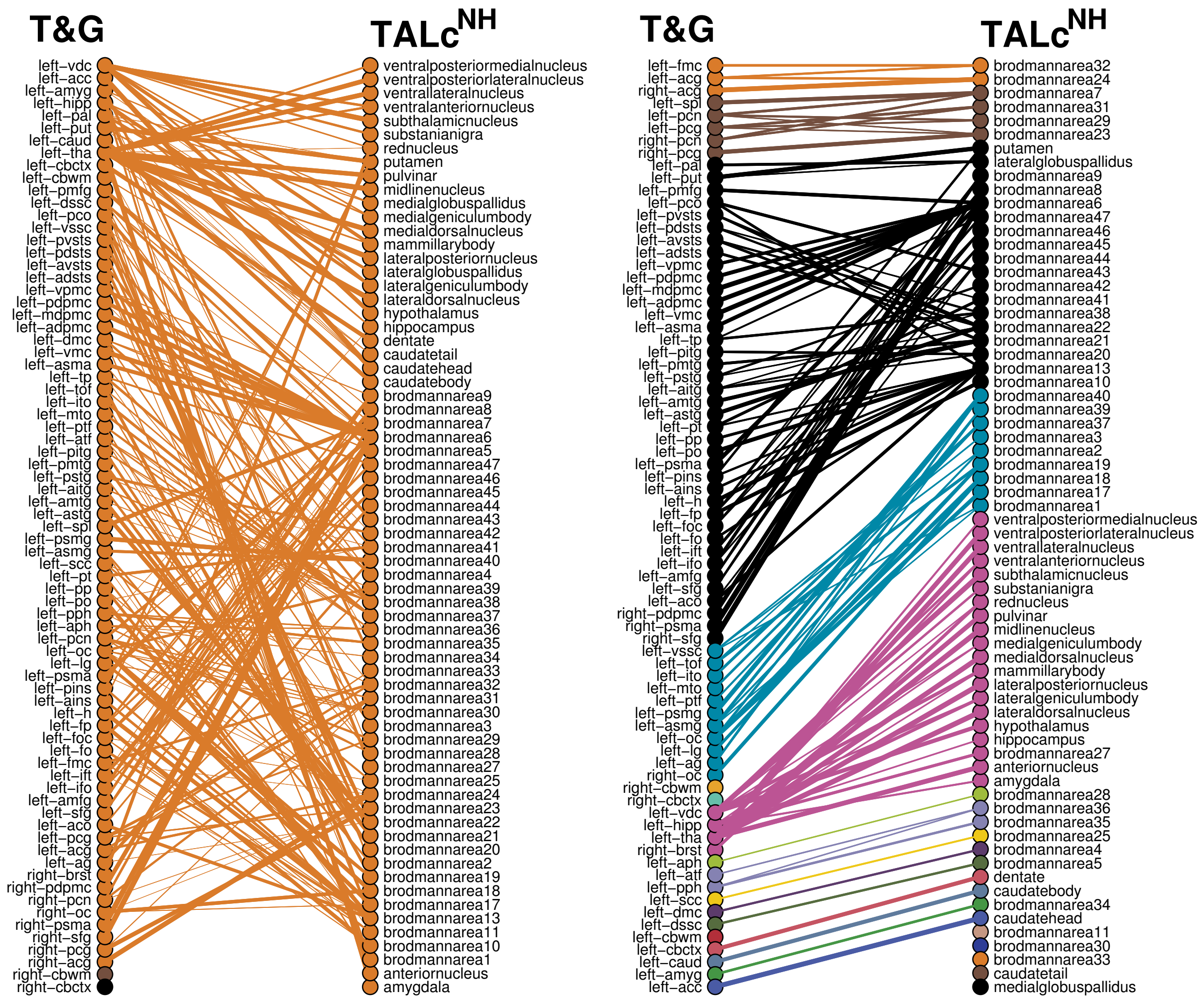

Figure 7: Left: Edges pruned up to $\theta = 0.10$; Right: Edges pruned up to $\theta = 0.25$.

2.7 TALc^{NH} - TALc

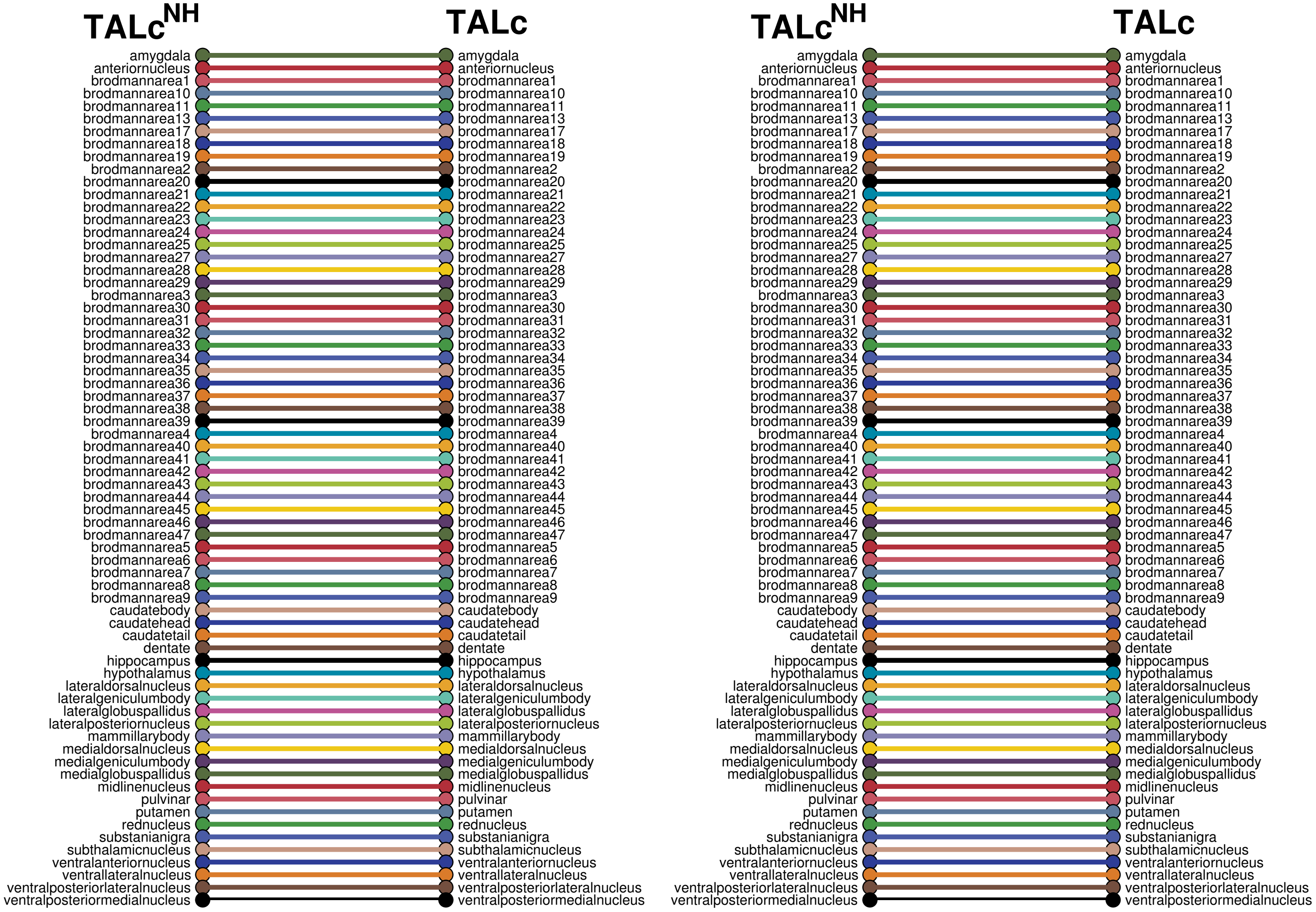

Figure 8: Left: Edges pruned up to $\theta = 0.10$; Right: Edges pruned up to $\theta = 0.25$.

2.8 TALc^{NH} - TALg

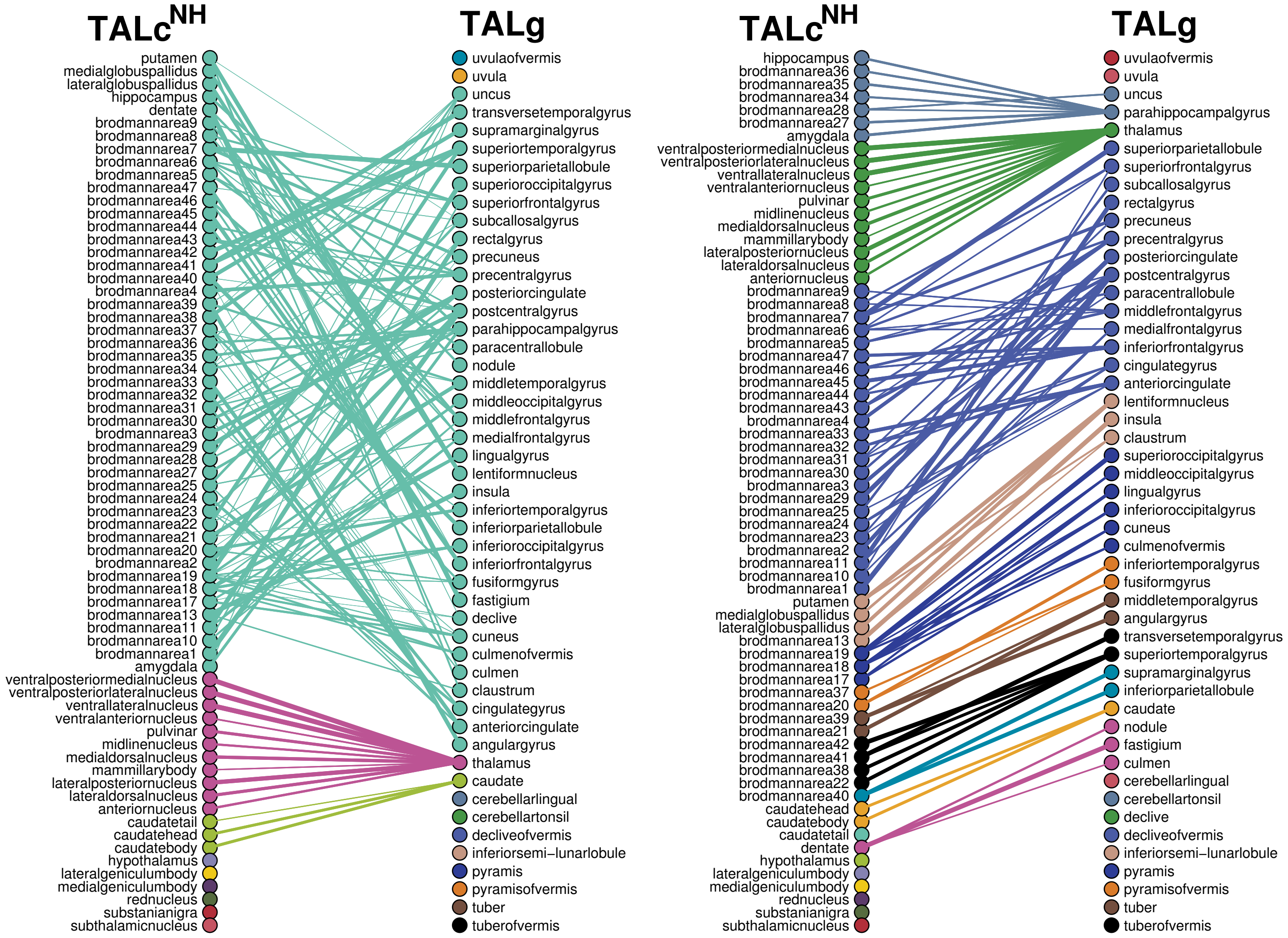

Figure 9: Left: Edges pruned up to $\theta = 0.10$; Right: Edges pruned up to $\theta = 0.25$.
