# Supplementary material for: The Brain Atlas Concordance Problem: Quantitative Comparison of Anatomical Parcellations
Source: PLoS One. 2009 Sep 29;4(9):e7200. doi: 10.1371/journal.pone.0007200 (PMC2748707; doi:10.1371/journal.pone.0007200)
Supplement: Table S1 — Global concordance measures for the comparison of parcellations computed using voxels in i) a cerebral cortex only mask , and ii) a subcortical only mask. (0.03 MB PDF) [file pone.0007200.s004.pdf]

	AAL	H-O	ICBM	LPBA40	T&G	TALc	TALg
AAL		0.6247	0.7315	0.6727	0.6020	0.5342	0.5894
		0.5336	0.7382	0.7666	0.6652	0.6564	0.4733
H-O	0.2860		0.6493	0.6930	0.5910	0.5033	0.5543
	0.1142		0.5662	0.6659	0.5639	0.6127	0.4461
ICBM	0.3989	0.3062		0.6847	0.6436	0.4930	0.6102
	0.1374	0.5102		0.7612	0.6547	0.6797	0.5867
LPBA40	0.3328	0.3726	0.4268		0.5423	0.4351	0.5603
	0.1358	0.5339	0.8774		0.7418	0.7238	0.5705
T&G	0.1329	0.2127	0.1467	0.1242		0.5100	0.5139
	0.1414	0.4102	0.7205	0.7181		0.6145	0.4716
TALc	0.0055	0.0048	0.0023	0.0015	0.0035		0.4674
	0.0339	0.1989	0.3317	0.3498	0.2495		0.8317
TALg	0.1769	0.1710	0.1946	0.2177	0.0691	0.0609	
	0.1361	0.0656	0.0045	0.0199	0.0177	0.1163	

Table S1. S-index (above diagonal entries) and Adjusted Rand Index (below diagonal) measures for the comparison of parcellations computed using voxels in i) a cerebral cortex only mask (white cells, black typeface), and ii) a subcortical only mask (gray cells, blue typeface). The union of the two masks was equivalent to the full left hemisphere GM mask used elsewhere in the paper.
